# Turnover Rates of Hepatic Collagen and Circulating Collagen-Associated Proteins in Humans with Chronic Liver Disease

**DOI:** 10.1371/journal.pone.0123311

**Published:** 2015-04-24

**Authors:** Martin L. Decaris, Claire L. Emson, Kelvin Li, Michelle Gatmaitan, Flora Luo, Jerome Cattin, Corelle Nakamura, William E. Holmes, Thomas E. Angel, Marion G. Peters, Scott M. Turner, Marc K. Hellerstein

**Affiliations:** 1 Department of Fibrosis, KineMed Inc., Emeryville, California, United States of America; 2 Department of Nutritional Science and Toxicology, University of California, Berkeley, California, United States of America; 3 Department of Medicine, Division of Gastroenterology, University of California San Francisco, San Francisco, California, United States of America; University of Navarra School of Medicine and Center for Applied Medical Research (CIMA), SPAIN

## Abstract

Accumulation and degradation of scar tissue in fibrotic liver disease occur slowly, typically over many years. Direct measurement of fibrogenesis, the rate of scar tissue deposition, may provide valuable therapeutic and prognostic information. We describe here results from a pilot study utilizing *in vivo* metabolic labeling to measure the turnover rate of hepatic collagen and collagen-associated proteins in plasma for the first time in human subjects. Eight subjects with chronic liver disease were labeled with daily oral doses of ^2^H_2_O for up to 8 weeks prior to diagnostic liver biopsy and plasma collection. Tandem mass spectrometry was used to measure the abundance and fractional synthesis rate (FSR) of proteins in liver and blood. Relative protein abundance and FSR data in liver revealed marked differences among subjects. FSRs of hepatic type I and III collagen ranged from 0.2–0.6% per day (half-lives of 4 months to a year) and correlated significantly with worsening histologic fibrosis. Analysis of plasma protein turnover revealed two collagen-associated proteins, lumican and transforming growth factor beta-induced-protein (TGFBI), exhibiting FSRs that correlated significantly with FSRs of hepatic collagen. In summary, this is the first direct measurement of liver collagen turnover *in vivo* in humans and suggests a high rate of collagen remodeling in advanced fibrosis. In addition, the FSRs of collagen-associated proteins in plasma are measurable and may provide a novel strategy for monitoring hepatic fibrogenesis rates.

## Introduction

Hepatic fibrosis is characterized by the slow accumulation of collagen-rich scar tissue, typically over many years, reflecting an imbalance in the synthesis and degradation of extracellular matrix (ECM) proteins [1]. Recent studies after curative anti-viral treatment of hepatitis C virus (HCV)-induced liver fibrosis have demonstrated the capacity of fibrotic scar tissue to resolve over time following removal of the fibrotic insult [2], suggesting, at least in the liver, a dynamic state of hepatic scar even in advanced fibrotic disease. Rates of progression of liver fibrosis have also been shown to be highly variable in HCV-infected patients, with slow, intermediate and rapid progressing populations [3]. Percutaneous liver biopsy represents the current standard for assessing fibrosis in liver disease, but this technique provides no insight into the rate of disease activity at any moment (i.e., the rate of ECM deposition or degradation), and is therefore insensitive for early detection of treatment efficacy, and not informative for predicting the likely trajectory of disease progression in an individual. Biomarkers that revealed the ongoing rate of tissue fibrogenesis (i.e., rate of ECM deposition) would in principle have considerable value for determining efficacy of anti-fibrotic therapies, selecting patients at risk for progression, as well as for clinical management of fibrotic liver disease [4].

Liver biopsies have intrinsic limitations for monitoring disease progression or resolution over time [2,5,6]. In addition to morbidity due to the invasive procedure, reliability of biopsy results has been limited by pathologist dependent semi-quantitative scoring and regional variability within the liver [7,8]. A number of less invasive serological and imaging approaches for staging liver fibrosis have therefore been developed over the past decade (e.g. Fibrotest, Fibroscan, Enhanced Liver Fibrosis score) [7], however these tests are often only indirectly linked to the fibrogenic process and have demonstrated variable accuracy when compared to biopsy, while still requiring multiple sampling points to track disease progression [7,9].

In principle, a rate-based measurement of fibrotic disease activity might provide superior insight regarding treatment effects and prognosis. Stable isotope tracers such as ^2^H_2_O, combined with mass spectrometric analysis, allow for the measurement of synthesis and degradation rates of biomolecules in the human body [10–13]. Using this approach, we have developed a proteome dynamics platform that quantifies the turnover rates of large numbers of proteins from tissue or body fluid samples and can be safely and routinely used in humans [14,15]. Following consumption of daily doses of ^2^H_2_O (e.g. for a few days to a few weeks), incorporation of deuterium (^2^H) into newly-synthesized proteins results in isotopic perturbations in newly synthesized molecules that are detectable via liquid chromatography/tandem mass spectrometry (LC-MS/MS). The fraction of each protein that was synthesized during the period of exposure to heavy water can then be calculated, revealing the FSR, (or fraction of the protein that is newly made per unit time), a metric of the synthesis and degradation (turnover) rates of each protein of interest [12,14–16].

In this pilot study, we describe the first direct measurements of the rate of hepatic collagen remodeling in human subjects with fibrotic liver disease. The FSRs of collagen and other liver proteins were compared to standard histopathologic scoring, and reveal evidence for ongoing remodeling in advanced fibrotic liver disease. We also identified two collagen-associated proteins in circulation, lumican and TGFBI, exhibiting FSRs that correlated significantly with hepatic collagen FSR.

## Materials and Methods

### Subject Recruitment, Tissue Collection, and Histology

A total of 11 subjects scheduled to undergo a diagnostic liver biopsy were recruited from the UCSF Hepatology clinic. There were 9 males and 2 females, mean age 49. Seven subjects had long standing chronic hepatitis C virus infection with inflammation and viremia; one subject had received a liver transplant (009) and had recurrent hepatitis C virus; three subjects were co-infected with human immunodeficiency virus, and one subject had autoimmune hepatitis (newly diagnosed) (Table 1). All HCV patients had HCV genotype 1 (37.5% genotype 1a and 62.5% genotype 1b) and all were HCV viremic. Subjects drank 50 ml of 70% ^2^H_2_O twice a day from the time of enrollment until the liver biopsy date, a period of between 3 to 8 weeks. Subjects returned for a liver biopsy, at which time blood, urine and an 18 gauge liver biopsy were collected. Eight subjects completed the study, with no adverse effects of heavy water intake reported. Histopathologic assessment of inflammation and fibrosis (Batts-Ludwig) [17] was performed on all eight subjects, with sufficient tissue remaining for hepatic proteomic analysis of six subjects. Only one subject had cirrhosis (Fibrosis score 4; Table 1). Plasma protein kinetic analyses were performed in all eight subjects. All procedures utilized in this study were approved by the University of California San Francisco Committee on Human Research. Subjects provided written informed consent and Declaration of Helsinki protocols were followed.

**Table 1:** Subject Demographics.

Subject ID #	003	004	005	007	008	009	010	011
Diagnosis	HCV, HIV	HCV, HIV	AIH	HCV	HCV, HIV	HCV,OLT	HCV	HCV
**Age at biopsy**	**44**	**63**	**43**	**59**	**39**	**45**	**67**	**59**
**Sex**	**M**	**M**	**M**	**M**	**M**	**M**	**F**	**M**
**D**_**2**_ **O Labeling Duration (days)**	**33**	**22**	**20**	**56**	**47**	**22**	**29**	**21**
**Liver biopsy** * **Fibrosis Score**	**3**	**0**	**2**	**1**	**2**	**4**	**1**	**0**
**Liver biopsy** * **Inflammation Score**	**3**	**0**	**1**	**1**	**2**	**2**	**2**	**2**
**Proteomic Tissue Analysis**	**Yes**	**Yes**	**Yes**	**Yes**	**No**	**Yes**	**No**	**Yes**
**Proteomic Plasma Analysis**	**Yes**	**Yes**	**Yes**	**Yes**	**Yes**	**Yes**	**Yes**	**Yes**

*both scores 0 to 4 (Batts Ludwig, 1995)

Details regarding diagnosis, heavy water labeling duration, pathology scoring and tissue samples analyzed from each clinical subject. Abbreviations: autoimmune hepatitis (AIH); hepatitis C virus (HCV); human immunodeficiency virus (HIV); orthotopic liver transplantation (OLT).

### Liver Tissue Preparation

Biopsied liver tissue (3–13 mg) was homogenized in water using a Fast Prep-24 bead mill (MP Biomedical, Burlingame CA), followed by hepatic protein quantification using the BCA Protein Assay Kit (Thermo, Rockford IL). Subsequent to trypsin digestion, proteins were precipitated with acetone at a 5:1 acetone:homogenate ratio by incubation at -20 C for 20 min followed by centrifugation at 2000 x *g* for 5 min at 4 C. Hepatic proteins were digested using both in-solution and in-gel approaches prior to LC-MS/MS analysis as described (S1 File).

### Plasma Preparation

Due to the wide dynamic range of protein concentration in plasma, analysis of plasma protein FSR was carried out following either the depletion of highly abundant proteins (broad screening of plasma protein kinetics) using a multi-affinity removal system spin cartridge (MARS Hu14; Agilent, Santa Clara CA), or immunoprecipitation (targeted enrichment of specific plasma proteins) using mass spectrometric immunoassay disposable automation research tips (MSIA; Thermo Fisher, Waltham MA). Detailed methods regarding sample preparation and trypsin digestion are provided in supplementary materials (S1 File).

### Plasma ^2^H_2_O Measurement

^2^H_2_O enrichment of body water for each subject was quantified using 75 μL of plasma as previously described [16]. Briefly, body water was evaporated from plasma via overnight incubation at 80 C. Samples were mixed in 10 M NaOH and acetone and underwent a second overnight incubation. This material was extracted in hexane and dried with Na_2_SO_4_ prior to gas chromatographic/mass spectrometric analysis alongside a standard curve of samples prepared at known ^2^H_2_O concentrations.

### LC-MS/MS Peptide Analysis

Trypsin-digested liver and plasma proteins were analyzed on Agilent 6550 quadrupole time-of-flight mass spectrometers fitted with a 1260 Chip Cube nano ESI source (Agilent Technologies, Santa Clara CA). Peptides were separated chromatographically using a Polaris HR chip (Agilent #G4240-62030) consisting of a 360 nL enrichment column and a 0.075 x 150 mm analytical column, each packed with Polaris C18-A stationary phase with 3 μm particle size. Mobile phases were (A) 5% v/v acetonitrile and 0.1% formic acid in deionized water and (B) 95% acetonitrile and 0.1% formic acid in deionized water. Peptides were eluted at a flow rate of 350 nL/min with a 27 min LC gradient. Each sample was analyzed twice, once for protein/peptide identification in data-dependent MS/MS mode and once for peptide quantitative isotope abundance analysis in MS mode. In MS/MS mode, acquisition rates were 6 Hz for MS and 4 Hz for MS/MS with up to 20 precursors per cycle. Acquisition rate in MS mode was 0.6 Hz.

### Determination of Protein FSR and Relative Abundances

Protein identification, FSR calculations and data filtering criteria are presented in detail (S1 File), similar to that previously described [14,16]. Briefly, software developed at KineMed, Inc. was used to calculate peptide elemental composition and curve-fit parameters for determining peptide isotope enrichment in newly synthesized proteins during the period of heavy water exposure, based on precursor body water enrichment and the number of amino acid C–H positions per peptide actively incorporating hydrogen and deuterium from body water. FSRs were calculated as rate constants for proteins with fractional synthesis values (f) measured as less than 0.90 (90% newly synthesized) by fitting to a mono-exponential rise-to-plateau curve [FSR = -ln(1-F)/t]. FSRs for proteins exhibiting fractional synthesis greater than 90% new are reported for the purpose of heat map visualization, but were omitted from quantitative correlation analyses. Kinetic data from liver protein digests were acquired from 3 replicate injections, and data were filtered to exclude proteins with fewer than two peptide isotope measurements unless otherwise noted.

To account for variability in subject labeling time, kinetic results were normalized for the duration of label exposure. Fitting to a mono-exponential rise-to-plateau curve [12,15] takes into account the specific length of time the subject was labeled. The assumption of this calculation is that each protein rises to a fully replaced state at plateau, with 100% newly synthesized proteins present. In a case where this assumption is incorrect, FSR calculation measures the actively turning over pool for a molecule, which is the parameter of interest in a kinetic study.

Label free protein quantification was performed following extraction of the total ion current of each isotope cluster from MS datasets using Agilent Mass Hunter software, for peptides identified in tandem MS/MS analyses [18]. Extracted ion abundances for all datasets were mean centered to one another to remove technical bias and protein abundance was inferred from the median peptide abundance considering the top 33% most abundant peptides attributed to each protein, with the software package Inferno (https://code.google.com/p/inferno4proteomics/) derived from Dante [19].

### Statistical Analyses

Microsoft Excel (Microsoft, Redmond WA) and GraphPad Prism (GraphPad Software Inc., La Jolla CA) were used to test statistical significance of linear regression analyses comparing FSR of liver collagen to histopathologic fibrosis score, FSRs of liver collagen and plasma proteins, and relative hepatic protein abundance to histological fibrosis score. Heat maps were generated using Inferno.

## Results

### Study Design and Subject Demographics

**Fig 1:**
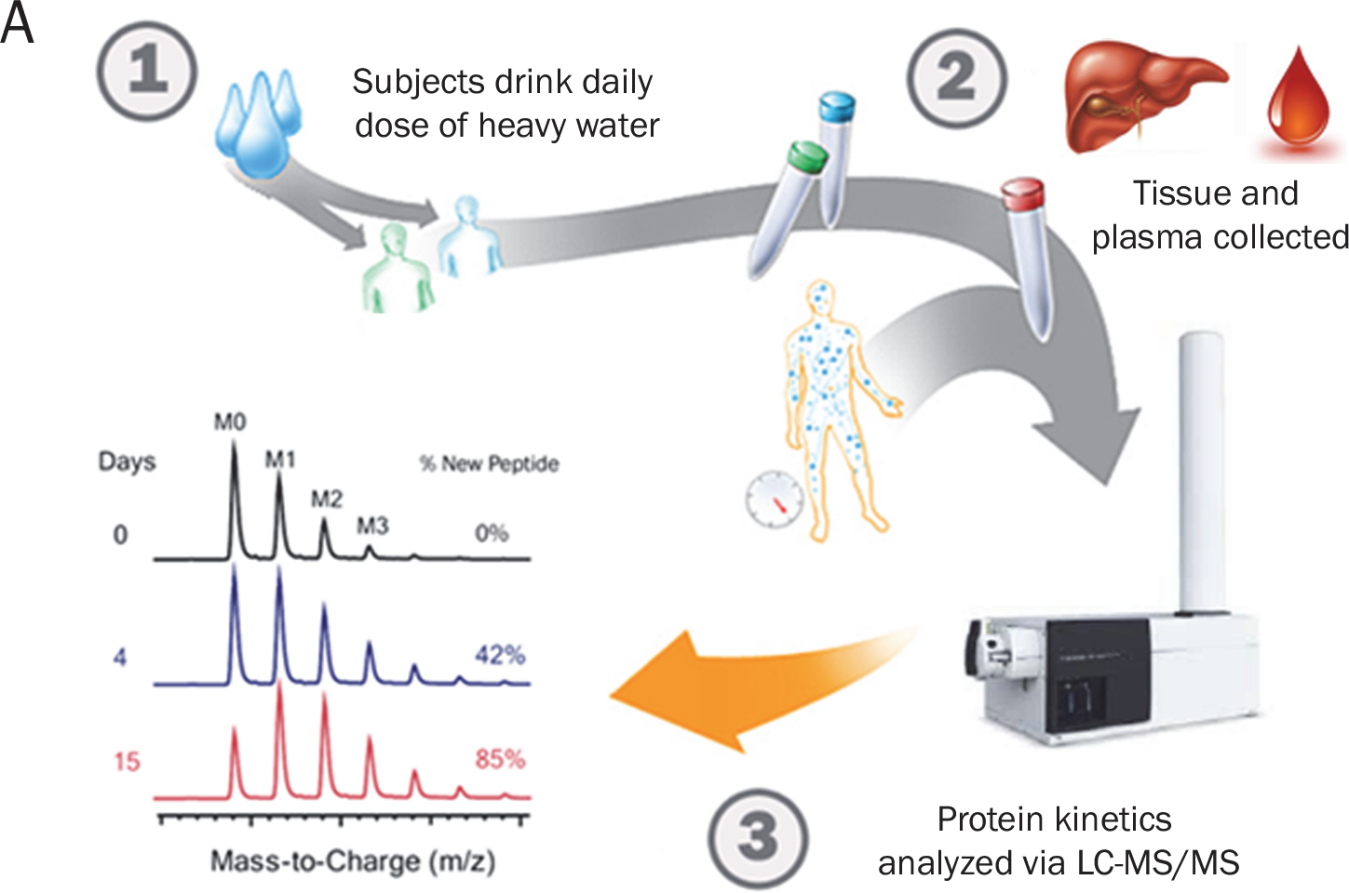
Overview of Study Design. Overview of the stable-isotope labeling strategy employed to measure hepatic and plasma protein turnover in human subjects with chronic liver disease.

An overview of the experimental study design is shown (Fig 1). Eight subjects consumed heavy water for periods ranging from 20 to 56 days prior to diagnostic liver biopsy and plasma collection. Pathology scoring of liver biopsies resulted in a range of fibrosis scores (F0-F4) documented within the patient population (Table 1). Proteomic data from plasma for all 8 subjects and from liver tissue in 6 of 8 subjects were analyzed. Histological results were blinded to investigators until data for non-targeted proteomic screening and kinetic analyses of liver and plasma proteins were finalized. All proteomic data was obtained through in-solution digestion of proteins unless otherwise noted.

### Comparison of Hepatic Protein FSRs and Abundances

A total of 356 liver proteins met pre-defined filtering criteria for the calculation of relative abundance between subjects (S1 Table). A subset of those proteins, 102 in total, also met the more stringent filtering criteria for accurate calculation of fractional synthesis rates (S2 Table). Individual proteins with relative abundances most positively correlated with histologic fibrosis score included multiple collagen subtypes (e.g. type I, III, and VI), collagen-associated ECM proteins (e.g. lumican and TGFBI), cytoskeletal proteins (e.g. desmin and talin-1) and histones (Table 2). Individual liver proteins with FSRs that most positively correlated with histologic fibrosis score also included collagen subtypes (e.g. type I and VI), as well as several mitochondrial proteins (Table 3).

**Table 2:** List of 20 hepatic proteins with relative abundances observed to most significantly positively correlate with histological fibrosis score.

Liver Protein	UniProt Accession #	Correlation to Fibrosis Score (r^2^)
**Histone H1.2**	**P16403**	**0.89**
**Gamma-glutamyltransferase 5**	**P36269**	**0.88**
**Peroxiredoxin-1**	**Q06830**	**0.88**
**Collagen alpha-1(III) chain**	**P02461**	**0.81**
**17-beta-hydroxysteroid dehydrogenase type 6**	**O14756**	**0.76**
**Profilin-1**	**P07737**	**0.76**
**Collagen alpha-2(I) chain**	**P08123**	**0.73**
**Serum albumin**	**P02768**	**0.73**
**Talin-1**	**Q9Y490**	**0.75**
**Histone H2B type 1-B**	**P33778**	**0.70**
**Collagen alpha-3(VI) chain**	**P12111**	**0.69**
**Collagen alpha-1(VI) chain**	**P12109**	**0.67**
**Desmin**	**P17661**	**0.66**
**Lumican**	**P51884**	**0.64**
**40S ribosomal protein S8**	**P62241**	**0.64**
**Annexin A2**	**P07355**	**0.63**
**TGF-beta-induced protein ig-h3**	**Q15582**	**0.60**
**Ubiquitin-60S ribosomal protein L40**	**P62987**	**0.60**
**Heat shock protein beta-1**	**P04792**	**0.59**
**Aminoacylase-1**	**Q03154**	**0.58**

**Table 3:** List of 10 hepatic proteins with fractional synthesis rates (FSRs) that most significantly positively correlate with histological fibrosis score.

Liver Protein	UniProt Accession #	Correlation to Fibrosis Score (r^2^)
**Collagen alpha-1(I) chain**	**P02452**	**0.79**
**Hemoglobin subunit alpha**	**P69905**	**0.72**
**Malate dehydrogenase, mitochondrial**	**P40926**	**0.71**
**Electron transfer flavoprotein subunit alpha, mitochondrial**	**P13804**	**0.71**
**Carbamoyl-phosphate synthase [ammonia], mitochondrial**	**P31327**	**0.70**
**Aspartate aminotransferase, mitochondrial**	**P00505**	**0.70**
**Hemoglobin subunit delta**	**P02042**	**0.70**
**3-ketoacyl-CoA thiolase, mitochondrial**	**P42765**	**0.69**
**Enoyl-CoA hydratase, mitochondrial**	**P30084**	**0.67**
**Collagen alpha-1(VI) chain**	**P12109**	**0.66**

**Fig 2:**
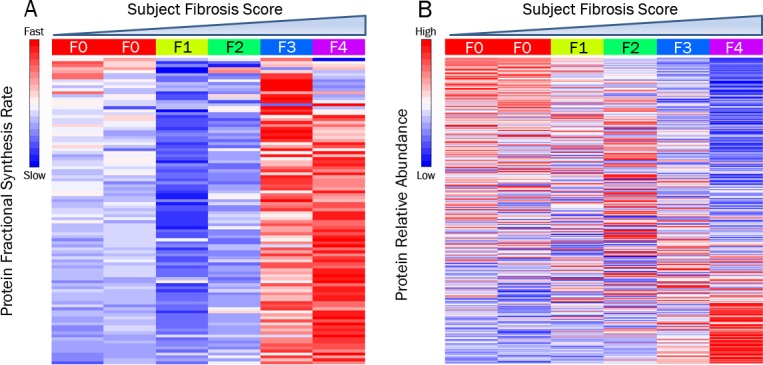
Hepatic Protein Turnover Rate vs Abundance. Heat maps displaying hepatic protein FSR (A) and relative abundance (B) for 6 human subjects with chronic liver disease. Protein data are row-scaled, with the columns representing each subject sorted by increasing histological fibrosis score. Abbreviation: fractional synthesis rate (FSR).

Comparison of overall protein FSRs with histological fibrosis score revealed that the majority of proteins observed (>90%) had higher FSRs in association with severity of fibrosis (Fig 2A). These data indicate a nearly global increase in FSR of liver proteins, i.e., a high-turnover state, associated with more advanced fibrotic liver disease. In contrast, a comparison of protein abundances to histological fibrosis score revealed a roughly even distribution of proteins with increased or reduced relative abundances across the proteome, reflecting shifts in overall liver protein composition in individual subjects (Fig 2B).

### Hepatic Collagen FSRs

**Fig 3:**
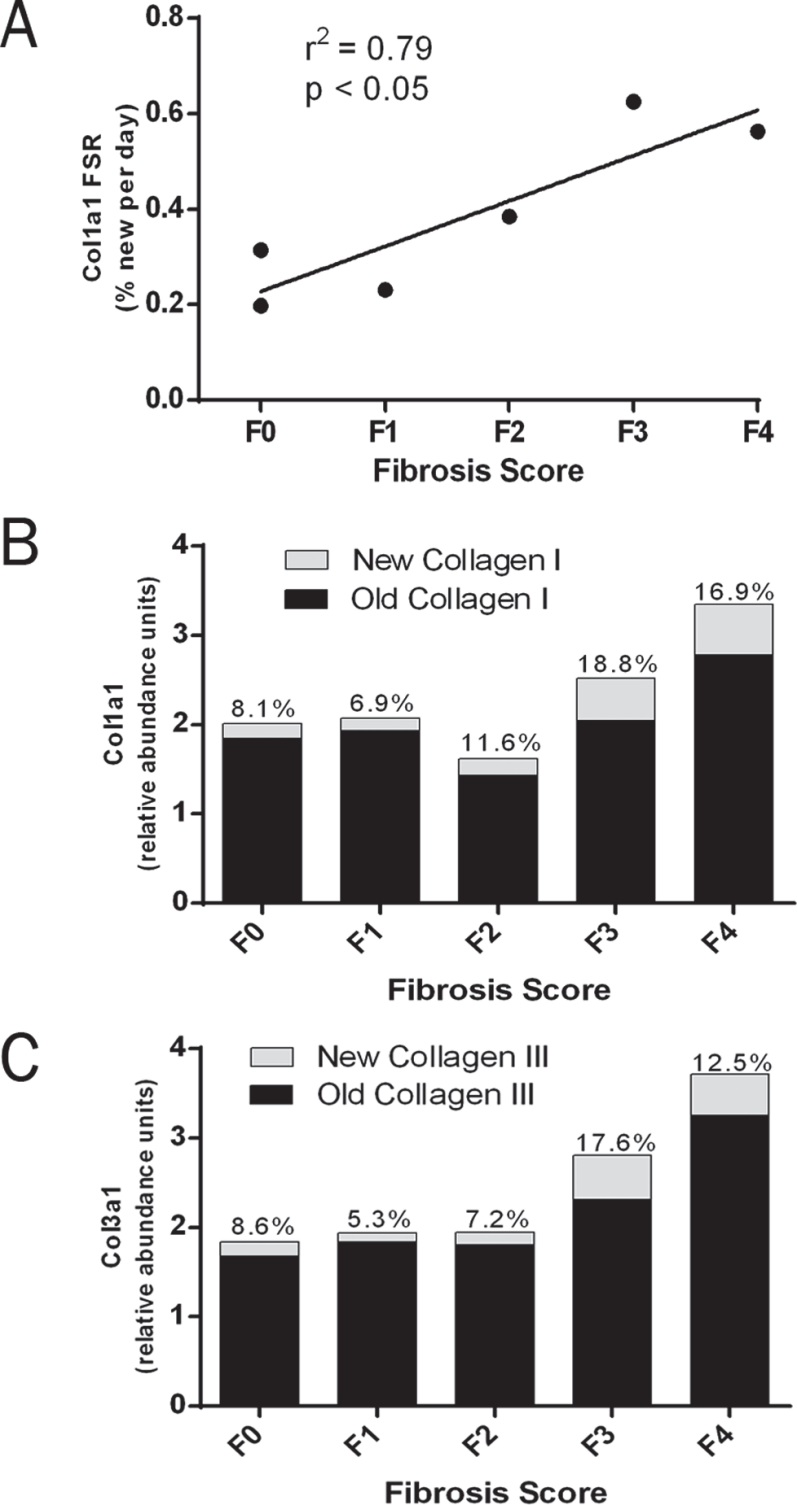
Comparison of Hepatic Collagen Kinetics and Histologic Fibrosis Score. (A) Linear regression of hepatic type I collagen FSR (% new per day) and histopathologic fibrosis score in human subjects with chonic liver disease. (B,C) Bar graphs depicting the relative abundance of unlabeled (old) and labeled (new) hepatic type I collagen (B) and type III collagen (C) per unit mass of liver protein, normalized to 30 days of labeling *in vivo*. Collagen FSR values (% new per 30 days) are displayed above each bar. Values shown represent the subject or the mean of subjects with each histopathology score. Abbreviation: fractional synthesis rate (FSR).

Type I and III collagens represent the major components of fibrous scar tissue in the liver. Calculation of hepatic protein FSRs revealed a significant positive correlation (p< 0.05) between type I collagen FSR and the severity of liver fibrosis as assessed by histological scoring (Fig 3A). FSRs for collagen types I and III ranged from 0.2–0.6% per day, representing half-lives ranging from 120–350 days. Expression of collagen FSR as a monthly rate (% newly synthesized per 30 days) was combined with label-free quantitation of relative collagen abundance to calculate absolute synthesis rates. These are represented visually as the fraction of old (unlabeled) and new (labeled) type I and III collagen present after 30 days in patients assigned different fibrotic scores (Fig 3B and 3C). The quantity of newly synthesized type I and III collagen was roughly 3-fold higher in those patients with more severe fibrosis scores on histopathology (F3-F4) compared to those assigned lesser scores (F0-F2), in this pilot data set.

### Comparison of FSRs of Hepatic Collagen and Plasma Proteins

**Fig 4:**
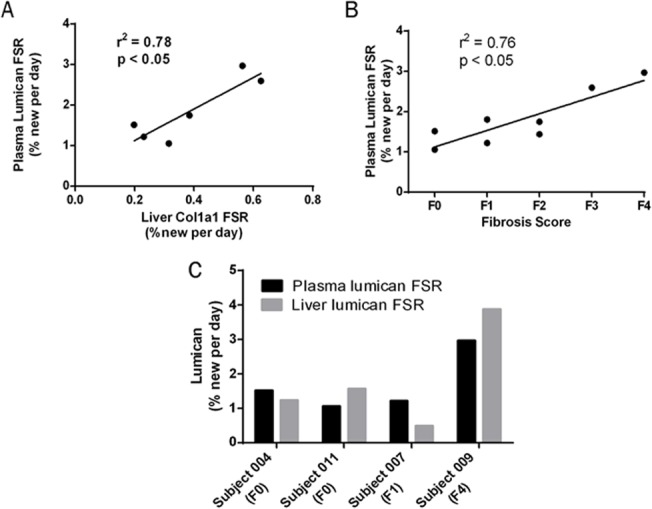
Comparison of Plasma Lumican FSR to Hepatic Collagen Kinetics and Histopathologic Fibrosis Score. (A) Linear regression of FSR of plasma lumican vs FSR of hepatic collagen in human subjects with chronic liver disease. (B) Linear regression of plasma lumican FSR vs histopathologic fibrosis score in human subjects with chronic liver disease. (C) Comparison of FSRs of liver and plasma lumican in individual subjects in whom liver values were measurable. Abbreviation: fractional synthesis rate (FSR).

Plasma protein FSRs from each subject were measured using samples collected at time of biopsy (S3 Table). Due to the extended labeling durations that were used here to quantify the relatively slow turnover of hepatic collagen, nearly all plasma proteins were found to have fractional synthesis values that approached 100%, which prevents accurate calculation of FSR. Lumican, a small leucine-rich proteoglycan known to play a role in collagen fibrillogenesis and the development of hepatic fibrosis [20–22], was an exception, with FSRs in plasma ranging from 1–3% per day. FSR of plasma lumican correlated significantly with the FSR of type I collagen in the liver (p < 0.05; Fig 4A), as well as with histopathologic fibrosis score (p < 0.05; Fig 4B). Although intrahepatic lumican FSR was not measurable in each subject, the FSR of lumican was calculable from one or more peptides in 4 of 6 tissue samples. Hepatic lumican FSRs were similar to those values measured in plasma (Fig 4C).

**Fig 5:**
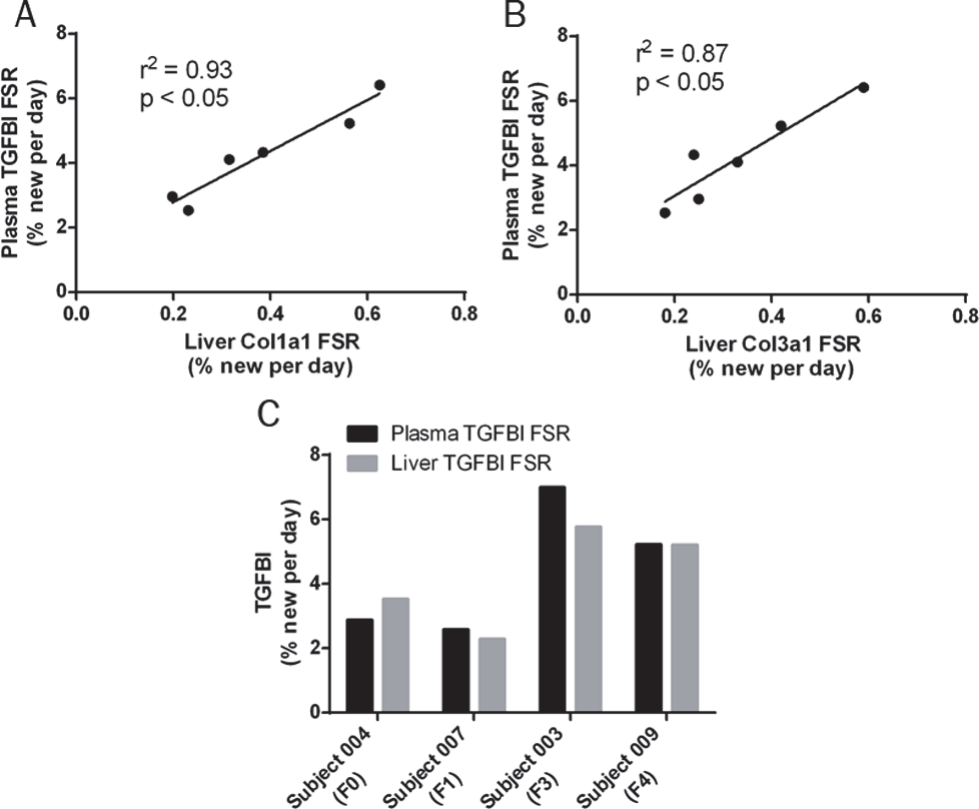
Comparison of Plasma TGFBI FSR to Hepatic Collagen Kinetics. (A) Linear regression of FSR of plasma TGFBI vs FSR of hepatic type I collagen in human subjects with chronic liver disease. (B) Linear regression of FSR of plasma TGFBI vs FSR of hepatic type III collagen in human subjects with chronic liver disease. (C) Comparison of FSRs of liver and plasma TGFBI in individual subjects in whom liver values were measurable. Abbreviations: fractional synthesis rate (FSR); transforming growth factor beta-induced protein (TGFBI).

We then evaluated the FSR of another collagen-associated protein, TGFBI, in plasma. TGFBI is a collagen-binding protein secreted in response to transforming growth factor beta activation. Although not abundant enough in plasma to be observed in our initial shotgun screening of plasma proteins, TGFBI was detectable following enrichment by immunoprecipitation and was found to have a relatively slow FSR in the plasma, ranging between 2%–7% per day across the 8 subjects. Comparison of FSR of plasma TGFBI with FSRs of hepatic collagens revealed significant correlations to both type I collagen and type III collagen (p < 0.05; Fig 5A and 5B). Gel-based fractionation of liver proteins was required to measure FSRs of one or more hepatic TGFBI peptides. In four subjects, FSR of plasma TGFBI matched closely with FSR of hepatic TGFBI (Fig 5C).

## Discussion

We measured, for what we believe to be the first time, *in vivo* turnover of collagen and collagen-related proteins in liver tissue and plasma in humans with chronic liver disease. Several potentially important results emerged, including the range of hepatic collagen turnover rates in human subjects with chronic liver disease, the fact that active collagen remodeling is observed in human subjects with advanced fibrotic disease, and the finding that FSRs of circulating collagen-associated proteins could be measured in humans and significantly correlated with FSRs of hepatic collagen.

Hepatic type I and III collagen turnover rates ranged from 0.2–0.6% per day, representing half-lives of approximately 4 months to 1 year. Importantly, liver collagen remodeling (turnover) rates were high in patients with advanced fibrotic disease. This preliminary observation of high FSR of hepatic collagen in patients with high histopathologic fibrosis scores suggests that not only hepatic collagen accumulation, but also hepatic collagen remodeling remains active during fibrotic disease progression in the liver. If confirmed in larger clinical populations stratified by distinct liver disease etiologies (here the majority of patients having viral hepatitis), this finding would have important therapeutic implications, as anti-fibrogenic agents might remain effective in resolving scar tissue in late-stage fibrotic disease. Persistence of active fibrogenesis in advanced fibrotic liver disease might, for example, explain clinical observations such as the recent report that the clinical response to obeticholic acid (OCA), a farnesoid-X-receptor agonist, in non-alcoholic steatohepatitis (NASH) patients was more pronounced in advanced fibrotic disease than in early disease [23].

The heavy water labeling method described here provides a technique for addressing this important question—does fibrogenesis rate accelerate with progression of fibrotic disease—in larger populations of patients with different etiologies of liver disease. While here we utilized linear regression analyses to compare FSR (a continuous variable) to histopathology score (a categorical variable), future studies may benefit from continuous readouts of fibrotic disease severity, such as transient elastography or histomorphometry, that are capable of differentiating subjects with the same histopathology scores.

Another potentially important observation was that the FSRs of several mitochondrial proteins were elevated in subjects with more severe liver fibrosis. In contrast to ECM proteins, however, there was a relative decrease in mitochondrial protein abundance observed by label-free analysis. The combination of low abundance and high FSR of mitochondrial proteins is consistent with a high rate of mitochondrial protein degradation in advanced liver disease rather than impaired mitochondrial biogenesis. These findings provide a possible kinetic basis for the previously described link between mitochondrial dysfunction and development of fibrosis in chronic liver disease [24,25], i.e. the proteomic data shown here suggests that mitophagy rather than mitochondrial biogenesis may be dysregulated. The observation that hemoglobin FSR correlates with histologic liver fibrosis score, reflecting shortened red cell life-span, will also be interesting to confirm in larger studies and may provide a functional metric of splenomegaly, portal hypertension and other factors contributing to intravascular hemolysis [26].

The primary objective of our study was to measure tissue collagen synthesis, a process that we presumed to be slow and in fact demonstrate here is slow (half-lives of 4 months to a year). As such, our study design was not optimized for the analysis of faster turnover proteins, including the majority of the more abundant plasma proteins, as we have shown previously in humans [14]. Nearly all plasma proteins that were detected following in-solution digest were close to fully labeled (i.e. > 90% newly synthesized) after 3 to 8 weeks of heavy water intake. A basic principle of tracer kinetics is that fitting to an exponential may not allow accurate estimates of FSR as an analyte becomes close to fully labeled [12]. It is one of our general data filtering rules to omit proteins that have FSR values exceeding 90%, as we have done here. We were still able to identify, however, two collagen-associated ECM proteins (lumican and TGFBI) in the plasma with FSRs that were measurable and could be correlated with FSR of hepatic collagen. Lumican and TGFBI have previously been reported as tissue markers of liver fibrosis [21,22,27–29]. We observed in this pilot study that despite potentially deriving from many tissues in the body, the FSR of lumican and TGFBI in plasma correlated with the FSR of liver collagen. Although these results must be confirmed in larger patient populations, the agreement that we observed between liver and blood metrics of fibrogenesis rate suggests that the contribution to circulating ECM-related proteins from a large organ like the liver, when fibrogenic, may dominate over inputs from other tissues.

It is worth noting some important distinctions between measuring protein FSR in circulation compared to measuring protein concentrations as potential biomarkers of tissue-related disease. Plasma concentration of a protein synthesized in the liver is determined by the release or secretion rate of the protein into circulation, as well as the clearance rate of the protein from the bloodstream. Biochemical recovery and analytic sensitivity also influence measured concentrations of a protein in the circulation. None of these rates or analytic factors, however, has a fundamental relationship to the metric of interest, protein synthesis rate within the tissue. In contrast, the FSR of a protein measured in blood that was derived from a tissue is not affected by or related to its concentration in the blood. The FSR reflects the internal ratio of labeled/unlabeled species in the molecules detected, which is an intrinsic feature of the circulating molecules determined by their synthesis rate, independent of release rate, clearance rate, biochemical recovery, or analytical sensitivity. Measurements of a protein’s FSR in blood thereby have fundamental technical advantages over concentration-based measurements for detecting changes in tissue protein synthesis associated with disease.

This study has some important limitations. Because of the small sample size and the clinical heterogeneity, the results presented here should not be over-interpreted quantitatively. Additional experiments in larger, well-stratified clinical populations are necessary to confirm the data presented here. Collection of multiple blood draws to analyze ^2^H_2_0 body water enrichment and circulating protein kinetics at various labeling durations prior to biopsy would be beneficial to future studies, widening the breadth and accuracy of protein kinetic data.

## Conclusions

There exists a strong need for biomarkers of hepatic fibrogenesis which reflect rate of disease activity, predict disease progression and rapidly detect response to anti-fibrotic therapies. Stable isotope labeling with heavy water, combined with a modified LC-MS/MS proteomics approach, offers a simple and safe means of directly measuring the rate of ECM remodeling in hepatic tissue, as well as the FSRs of ECM-related proteins in circulation. In this pilot study several potentially important observations are reported, including a wide range of hepatic collagen turnover rates in human subjects with chronic liver disease (half-lives of 4 months to a year), the fact that active collagen remodeling occurs in human subjects with advanced fibrotic disease, and the significant correlation between the FSR of hepatic collagen with that of circulating collagen-associated proteins. Although these preliminary data did not test the hypothesis that fibrogenesis rates better predict disease progression or early response to therapy in comparison to histopathology, identification of circulating proteins with FSRs that correlate with hepatic collagen FSR allows future studies addressing such questions to be performed in a minimally invasive manner.

## Supporting Information

S1 File Supplementary methods. (DOCX)

S1 Table Hepatic protein relative abundance data. (PDF)

S2 Table Hepatic protein fractional synthesis data. (PDF)

S3 Table Plasma protein fractional synthesis data. (PDF)
