# Supplementary material for: Turnover Rates of Hepatic Collagen and Circulating Collagen-Associated Proteins in Humans with Chronic Liver Disease
Source: PLoS One. 2015 Apr 24;10(4):e0123311. doi: 10.1371/journal.pone.0123311 (PMC4409311; doi:10.1371/journal.pone.0123311)
Supplement: S1 File — (DOCX) [file pone.0123311.s001.docx]

**Supplementary Methods**

*Liver Tissue Preparation*

For in-solution digests, 80 μg of liver protein was denatured using ProteasMax™ surfactant (0.1%; Promega, Madison WI) and urea (4M) in 25 mM ammonium bicarbonate (pH=8). Samples were reduced with 5 mM *tris*(2-carboxyethyl)phosphine (TCEP) for 20 minutes at room temperature with mixing, followed by incubation with iodoacetamide (10 mM) in the dark for 20 minutes to chemically modify reduced cysteines. Following sample dilution, proteins were then digested with trypsin (Promega, Madison WI) at 37 C overnight. The following day formic acid was added to a total volume of 5%, and peptides were concentrated and desalted prior to LC-MS/MS using a C18 spec tip (Varian, Palo Alto CA).

For in-gel protein digests, samples were prepared similar to that previously described [[1](#_ENREF_1)]. 100 μg total protein was denatured with XT sample loading buffer (Bio-Rad) and reduced with TCEP (11.4 mM). Samples were heated to 95 C for 5 min, cooled to RT and incubated with iodoacetamide (30 mM) in the dark for 1 hr. Samples were then loaded and run on 4-20% polyacrylamide gels (Bio-Rad), stained with coomassie brilliant blue and scanned for densitometry. Gel bands for specific molecular weight ranges were cut, minced and rinsed with 25 mM NH_4_HCO_3_ in 50% Acetonitrile to destain. Gel pieces were then incubated overnight in 12.5 ng/μL trypsin in 25 mM NH_4_HCO_3_ to digest proteins. The following day, 5% formic acid/50% acetonitrile was added to gel pieces prior to bath sonication for 5 min. Supernatant was collected and the process repeated 1X. Combined supernatants were centrifuged, vacuum dried and reconstituted in 0.1% formic acid/3% acetonitrile prior to LC-MS/MS analysis.

*Plasma Preparation*

Plasma from 8 patients (10 µL) was depleted of high abundance proteins using a multi-affinity removal system spin cartridge (Hu14, Agilent, Santa Clara, CA) according to the manufacturer’s recommendations. Remaining proteins were quantified using the BCA Protein Assay Kit (Thermo, Rockford IL), and 50 ug of protein was isolated from each patient for trypsin digestion. Plasma proteins samples were denatured using ProteasMax™ surfactant (0.1%; Promega, Madison WI) and urea (4 M) in 25 mM ammonium bicarbonate (pH=8). The solution was reduced with TCEP (5 mM) for 20 min at room temperature with mixing, followed by incubation with Iodoacetamide (10 mM) in the dark for 20 min to chemically modify reduced cysteines. Plasma proteins were then digested with trypsin (Promega, Madison WI) at 37 C overnight. The following day formic acid was added to a total volume of 5%, and peptides were concentrated and desalted prior to LC-MS/MS using a C18 spec tip (Varian, Palo Alto CA).

Immunoprecipitation of TGFBI was performed using mass spectrometric immunoassay streptavidin disposable automation research tips (Thermo Fisher, Waltham MA). Tips were loaded with 3 μg of biotinylated antibody targeting TGFBI (BAF2935; polyclonal goat IgG; immunogen: recombinant human TGFBI; Lot #VZA0110061; R&D Systems) using a multichannel pipette. For immunoprecipitation of TGFBI, 200 μL plasma diluted 1:2 in PBS was cycled through each tip 500X. MSIA tips were then rinsed 3X with PBS and water to remove unbound proteins, followed by protein elution in 33% acetonitrile/0.4% trifluoroacetic acid. Eluent was dried by vacuum centrifugation and reconstituted in 50 mM NH_4_HCO_3_, 50% trifluoroethanol, 50 mM dithiothreitol, followed by incubation at 60 C for 1 hr. 50 mM iodoacetamide was then added to samples followed by incubation in the dark for 20 min. Samples were diluted, digested with trypsin overnight, spiked with 0.15% trifluoroacetic acid, vacuum centrifuged, and reconstituted in in 0.1% formic acid/3% acetonitrile prior to LC-MS/MS analysis.

*Determination of Protein Fractional Synthesis Rates*

Acquired MS/MS spectra were extracted and searched using Spectrum Mill Proteomics Workbench software (version B.04.00, Agilent Technologies, Santa Clara, CA) and a UniProtKB/Swiss-Prot human protein database (20,265 proteins, UniProt.org, release 2013_05). Fixed modifications (carbamidomethylation of cysteine) and variable modifications (oxidized methionine, pyroglutamic acid, hydroxylation of proline) were enabled with up to two missed cleavages permitted. Search results were autovalidated with a global false discovery rate of 1%. Proteins with scores greater than 11.0 were reported and a list of peptides with scores greater than 6 and scored peak intensities greater than 50% was exported from Spectrum Mill and condensed to a non-redundant peptide formula database using Excel. This database, containing peptide elemental composition, mass, and retention time was used to extract mass isotopomer abundances (M0-M3) of each peptide from corresponding MS-only acquisition files with the Find-by-Formula algorithm in Mass Hunter (version B.05.00, Agilent Technologies, Santa Clara, CA, USA). Software developed at KineMed, Inc. was used to calculate peptide elemental composition and curve-fit parameters for determining peptide isotope enrichment (EM0) in newly synthesized proteins during the period of heavy water exposure, based on precursor body water enrichment (p) and the number (n) of amino acid C–H positions per peptide actively incorporating hydrogen (H) and deuterium (D) from body water. Subsequent data handling was performed using Microsoft Excel templates, with input of precursor body water enrichment for each subject, to yield FSR data at the protein level from the peptides analyzed for each protein similar to that previously described [[1-3](#_ENREF_1)].

**References**

1. Price JC, Khambatta CF, Li KW, Bruss MD, Shankaran M, et al. (2012) The effect of long term calorie restriction on in vivo hepatic proteostatis: a novel combination of dynamic and quantitative proteomics. Mol Cell Proteomics.

2. Decaris ML, Gatmaitan M, Florcruz S, Luo F, Li K, et al. (2014) Proteomic Analysis of Altered Extracellular Matrix Turnover in Bleomycin-Induced Pulmonary Fibrosis. Mol Cell Proteomics.

3. Price JC, Holmes WE, Li KW, Floreani NA, Neese RA, et al. (2012) Measurement of human plasma proteome dynamics with (2)H(2)O and liquid chromatography tandem mass spectrometry. Anal Biochem 420: 73-83.
