# Supplementary material for: Turnover Rates of Hepatic Collagen and Circulating Collagen-Associated Proteins in Humans with Chronic Liver Disease
Source: PLoS One. 2015 Apr 24;10(4):e0123311. doi: 10.1371/journal.pone.0123311 (PMC4409311; doi:10.1371/journal.pone.0123311)
Supplement: S1 Table — (PDF) [file pone.0123311.s002.pdf]

Supplementary Table 1

		Relative protein quantitation (relative abundance units)						
		Subject #003	Subject #004	Subject #005	Subject #007	Subject #009	Subject #011	
	Fibrosis Score	3	0	2	1	4	0	Protein Relative Abundance vs Fibrosis Score (r^2)
**Liver Proteins**	**Uniprot Accession**							
14-3-3 protein beta/alpha	P31946	390016	171967	158083	153238	279392	167419	0.51
14-3-3 protein eta	Q04917	90935	296191	237619	502794	564718	282864	0.02
17-beta-hydroxysteroid dehydrogenase 13	Q7Z5P4	567760	262348	305142	218436	368650	284814	0.42
17-beta-hydroxysteroid dehydrogenase type 6	O14756	243073	106063	279516	124726	299060	159793	0.76
2,4-dienoyl-CoA reductase, mitochondrial	Q16698	931314	1130684	680662	753338	492344	451256	0.06
3-hydroxyacyl-CoA dehydrogenase type-2	Q99714	511021	468255	419662	348686	388496	410185	0.00
3-hydroxyanthranilate 3,4-dioxygenase	P46952	258141	166735	448526	167587	142616	222749	0.01
3-ketoacyl-CoA thiolase, mitochondrial	P42765	321019	441734	645040	292564	460308	699140	0.08
3-ketoacyl-CoA thiolase, peroxisomal	P09110	324774	406957	495029	239148	152220	388735	0.28
3-mercaptopyruvate sulfurtransferase	P25325	272940	467186	448559	511781	385653	244737	0.02
40S ribosomal protein S3	P23396	149467	231130	143076	234760	99627	246593	0.90
40S ribosomal protein S7	P62081	299910	179058	148195	196546	131081	81042	0.10
40S ribosomal protein S8	P62241	14371039	1734742	1402970	1539634	10137559	1054433	0.64
40S ribosomal protein SA	P08865	573125	422502	308623	361275	338146	327499	0.04
4-aminobutyrate aminotransferase, mitochondrial	P80404	242295	442823	652004	296564	139465	361289	0.21
4-hydroxyphenylpyruvate dioxygenase	P32754	366919	547351	298035	85995	444299	295323	0.02
4-trimethylaminobutyraldehyde dehydrogenase	P49189	206824	291306	543668	173594	108619	332114	0.13
60 kDa heat shock protein, mitochondrial	P10809	407786	527707	431567	238668	194147	299972	0.15
60S acidic ribosomal protein P0-like	Q8NHW5	157627	239155	252211	217989	178536	132906	0.02
60S acidic ribosomal protein P1	P05386	310096	316654	393704	266666	281847	406218	0.15
60S ribosomal protein L6	Q02878	179098	135891	183446	134197	152592	130491	0.41

Supplementary Table 1

		Relative protein quantitation (relative abundance units)						
		Subject #003	Subject #004	Subject #005	Subject #007	Subject #009	Subject #011	
	Fibrosis Score	3	0	2	1	4	0	Protein Relative Abundance vs Fibrosis Score (r^2)
6-phosphogluconate dehydrogenase, decarboxylating	P52209	267405	260327	69732	172029	171175	269377	0.11
78 kDa glucose-regulated protein	P11021	356153	521386	279327	366647	160969	392771	0.71
Abhydrolase domain-containing protein 14B	Q96IU4	474080	361309	489383	304351	416608	403295	0.26
Acetyl-CoA acetyltransferase, cytosolic	Q9BWD1	280092	101850	472538	259826	141371	155171	0.05
Acetyl-CoA acetyltransferase, mitochondrial	P24752	553273	828820	536354	487607	335621	720691	0.72
Aconitate hydratase, mitochondrial	Q99798	126768	505977	217783	343650	206941	498463	0.82
Actin, aortic smooth muscle	P62736	274081	1006743	289663	640546	1006683	1162560	0.15
Actin, cytoplasmic 1	P60709	1524953	1055774	684040	1089633	1623840	741735	0.54
Actin-related protein 3B	Q9P1U1	581457	297543	497708	213656	351224	375482	0.21
Acyl-coenzyme A synthetase ACSM2A, mitochondrial	Q08AH3	128759	246929	197206	226356	92803	110840	0.31
Acyl-coenzyme A synthetase ACSM2B, mitochondrial	Q68CK6	172592	285255	211214	162307	168352	154503	0.13
Acyl-coenzyme A thioesterase 1	Q86TX2	361557	445371	454688	338046	189739	405079	0.51
Adenine phosphoribosyltransferase	P07741	156540	115399	134323	185994	181011	84331	0.46
Adenylate kinase 2, mitochondrial	P54819	155228	298321	225916	182140	113274	388472	0.76
ADP/ATP translocase 1	P12235	208473	477125	370758	590026	455709	923308	0.41
ADP/ATP translocase 2	P05141	851979	361003	383389	311278	165660	1052450	0.10
ADP-ribosylation factor 1	P84077	360803	358984	354920	395486	463714	335952	0.48
Aflatoxin B1 aldehyde reductase member 3	O95154	177556	328636	236484	397982	440774	145232	0.09
Agmatinase, mitochondrial	Q9BSE5	344353	221665	171135	138647	90941	163255	0.00
Alanine aminotransferase 1	P24298	282167	193224	397684	155702	185960	306813	0.00
Alcohol dehydrogenase 1A	P07327	887477	1154516	2469488	1299993	574326	1643925	0.16
Alcohol dehydrogenase 1B	P00325	306952	942507	2966067	1036885	870296	1308014	0.02

Supplementary Table 1

		Relative protein quantitation (relative abundance units)						
		Subject #003	Subject #004	Subject #005	Subject #007	Subject #009	Subject #011	Protein Relative Abundance vs Fibrosis Score (r^2)
	Fibrosis Score	3	0	2	1	4	0	
Alcohol dehydrogenase 1C	P00326	461947	848326	77460	305137	232659	778711	0.47
Alcohol dehydrogenase 4	P08319	355682	481488	1993403	885862	407368	709220	0.01
Alcohol dehydrogenase 6	P28332	110775	411190	816238	495402	136221	225894	0.09
Alcohol dehydrogenase class-3	P11766	359819	515757	933728	442465	477505	107390	0.07
Aldehyde dehydrogenase X, mitochondrial	P30837	379957	212768	143706	168285	115559	124732	0.03
Aldehyde dehydrogenase, mitochondrial	P05091	400538	651008	1078053	608503	172180	628166	0.25
Aldehyde oxidase	Q06278	384453	384815	428579	535264	296361	259226	0.01
Aldo-keto reductase family 1 member C1	Q04828	322047	1421063	532538	447862	195031	210105	0.27
Aldo-keto reductase family 1 member C2	P52895	82753	127802	61249	66653	57674	158821	0.58
Aldo-keto reductase family 1 member C4	P17516	291163	266324	535466	312129	420554	243378	0.29
Alpha-1-antitrypsin	P01009	203992	296709	124437	99192	471559	105440	0.32
Alpha-2-macroglobulin	P01023	166236	178817	210252	278319	313038	125897	0.32
Alpha-actinin-1	P12814	177938	253328	214395	209370	234626	259144	0.31
Alpha-actinin-2	P35609	151998	95526	105238	100487	220393	613087	0.09
Alpha-actinin-4	O43707	312283	334929	256249	210717	380638	332760	0.07
Alpha-aminoadipic semialdehyde dehydrogenase	P49419	109513	194235	433547	292611	110217	251378	0.15
Alpha-enolase	P06733	362304	357972	425754	329380	442979	587080	0.03
Amine oxidase [flavin-containing] A	P21397	213010	208951	362264	394705	206918	197694	0.01
Amine oxidase [flavin-containing] B	P27338	384404	409125	407970	404062	153693	214525	0.10
Aminoacylase-1	Q03154	391277	291920	326906	216752	370988	279144	0.58
Annexin A2	P07355	286098	166004	180235	135674	792396	178227	0.63
Annexin A4	P09525	212014	189509	226986	216985	401858	253651	0.43
Annexin A5	P08758	267192	198464	45294	213419	414263	177124	0.33
Annexin A6	P08133	410410	334099	314023	355178	362150	376798	0.08

Supplementary Table 1

		Relative protein quantitation (relative abundance units)						
		Subject #003	Subject #004	Subject #005	Subject #007	Subject #009	Subject #011	Protein Relative Abundance vs Fibrosis Score (r^2)
	Fibrosis Score	3	0	2	1	4	0	
Apolipoprotein A-I	P02647	246216	365228	165620	204408	404322	117578	0.18
Apoptosis-inducing factor 1, mitochondrial	O95831	210859	253312	301006	263138	146035	206075	0.24
Arginase-1	P05089	497808	500998	610382	526215	258129	385222	0.13
Argininosuccinate lyase	P04424	307292	318335	282359	454260	220608	290972	0.22
Argininosuccinate synthase	P00966	433991	287147	1055696	537575	538243	905545	0.00
Arylacetamide deacetylase	P22760	138106	340196	126356	92496	140320	179245	0.27
Aspartate aminotransferase, cytoplasmic	P17174	410791	650264	557384	441305	212504	297995	0.24
Aspartate aminotransferase, mitochondrial	P00505	720319	1090283	761722	1232983	490247	1014776	0.77
ATP synthase subunit alpha, mitochondrial	P25705	746211	817706	423800	560193	354951	899795	0.50
ATP synthase subunit b, mitochondrial	P24539	256631	289302	340743	307720	214527	487545	0.50
ATP synthase subunit beta, mitochondrial	P06576	468671	534628	485517	424796	294116	665903	0.66
ATP synthase subunit delta, mitochondrial	P30049	137597	74809	100390	124564	103436	289559	0.14
ATP synthase subunit O, mitochondrial	P48047	394723	559874	421407	527444	315991	561667	0.98
ATP-binding cassette sub-family D member 3	P28288	187378	101982	92925	195004	125141	163522	0.00
Basement membrane-specific heparan sulfate proteoglycan core protein	P98160	88374	68479	76187	167590	111062	52982	0.07
Beta-enolase	P13929	355426	933086	336765	315625	772491	556946	0.02
Betaine--homocysteine S-methyltransferase 1	Q93088	532751	732260	1214333	789318	317889	988298	0.35
Betaine--homocysteine S-methyltransferase 2	Q9H2M3	152215	408705	225006	256654	123515	249318	0.74
Bifunctional ATP-dependent dihydroxyacetone kinase/FAD-AMP lyase (cyclizing)	Q3LXA3	393692	442637	281790	371288	180997	347845	0.48
Bile acid-CoA:amino acid N-acyltransferase	Q14032	658194	538211	423559	210618	263570	470954	0.02

Supplementary Table 1

		Relative protein quantitation (relative abundance units)						
		Subject #003	Subject #004	Subject #005	Subject #007	Subject #009	Subject #011	
	Fibrosis Score	3	0	2	1	4	0	Protein Relative Abundance vs Fibrosis Score (r^2)
Bile acyl-CoA synthetase	Q9Y2P5	186923	246297	359361	356951	133219	225478	0.21
Bile salt sulfotransferase	Q06520	216896	850168	407157	319380	238205	581976	0.67
C-1-tetrahydrofolate synthase, cytoplasmic	P11586	464795	471396	426200	569801	495903	260898	0.14
Calcium-binding mitochondrial carrier protein Aralar2	Q9UJS0	264164	343665	305605	211060	159599	221362	0.22
Calmodulin	P62158	956523	301884	567340	246516	540482	1149017	0.00
Calnexin	P27824	610268	479192	438074	319380	147362	431878	0.11
Calreticulin	P27797	513011	382900	459027	357036	413608	343090	0.45
Carbamoyl-phosphate synthase [ammonia], mitochondrial	P31327	608634	837655	1575960	1184389	378147	1128944	0.27
Carbonic anhydrase 1	P00915	320191	307672	243940	123773	354229	142137	0.41
Carbonic anhydrase 2	P00918	273366	321512	518038	211920	287929	336990	0.00
Carbonyl reductase [NADPH] 1	P16152	302504	706811	352507	313125	384308	562570	0.47
Catalase	P04040	406353	416907	407259	469451	254051	457036	0.63
Cathepsin D	P07339	557319	365235	266682	350858	495107	337625	0.43
Clathrin heavy chain 1	Q00610	150323	145481	123770	198036	122506	211249	0.42
Cofilin-1	P23528	335709	273318	141074	340983	448902	396019	0.05
Collagen alpha-1(I) chain	P02452	252164	155662	161999	207446	334606	246411	0.44
Collagen alpha-1(III) chain	P02461	280410	189953	194664	193896	371597	177778	0.81
Collagen alpha-1(IV) chain	P02462	184860	138707	149828	168323	356365	174017	0.56
Collagen alpha-1(VI) chain	P12109	317392	238331	254398	211815	551534	244783	0.67
Collagen alpha-1(XIV) chain	Q05707	196106	115734	135591	131696	428986	220098	0.48
Collagen alpha-2(I) chain	P08123	366521	227369	319017	308505	576338	315992	0.73
Collagen alpha-2(VI) chain	P12110	255668	194062	209355	301792	269713	144362	0.34
Collagen alpha-3(VI) chain	P12111	274293	188941	211864	219458	460782	217783	0.69

Supplementary Table 1		Relative protein quantitation (relative abundance units)						
		Subject #003	Subject #004	Subject #005	Subject #007	Subject #009	Subject #011	
	Fibrosis Score	3	0	2	1	4	0	Protein Relative Abundance vs Fibrosis Score (r^2)
Complement C3	P01024	134495	187505	128316	167484	213527	117158	0.11
Creatine kinase M-type	P06732	488670	67986	109996	272188	434902	2066700	0.11
Cystathionine gamma-lyase	P32929	144734	124180	368802	215285	170095	320540	0.04
Cytochrome b5	P00167	1284056	3223041	2191395	2519420	770280	3053207	0.98
Cytochrome b-c1 complex subunit 2, mitochondrial	P22695	233557	146989	175112	367130	472931	220582	0.36
Cytochrome c oxidase subunit 2	P00403	178913	297586	252800	419380	157805	445196	0.71
Cytochrome c oxidase subunit 5A, mitochondrial	P20674	365566	381588	466471	374907	217881	352591	0.24
Cytochrome P450 2A6	P11509	292046	277246	245900	421409	117824	142911	0.07
Cytochrome P450 2C8	P10632	423184	538536	469606	230266	159437	531918	0.42
Cytochrome P450 2C9	P11712	529717	420198	246868	255992	73289	393433	0.19
Cytochrome P450 2E1	P05181	174529	265211	419962	327835	234160	129019	0.00
Cytochrome P450 3A4	P08684	96634	183501	147762	734315	126016	551762	0.32
Cytoplasmic aconitate hydratase	P21399	325924	198412	339595	277914	177651	198565	0.04
Cytosol aminopeptidase	P28838	181415	365615	265500	173990	262172	238265	0.10
Cytosolic 10-formyltetrahydrofolate dehydrogenase	O75891	142417	240402	390816	239555	133561	181877	0.09
Cytosolic non-specific dipeptidase	Q96KP4	212149	192455	276502	212721	288105	224476	0.44
D-beta-hydroxybutyrate dehydrogenase, mitochondrial	Q02338	348290	623219	509889	776160	182364	223237	0.21
D-dopachrome decarboxylase	P30046	211333	528448	494189	585031	316231	842264	0.69
D-dopachrome decarboxylase-like protein	A6NHG4	378851	1409516	759446	401136	503371	1089923	0.54
Delta(3,5)-Delta(2,4)-dienoyl-CoA isomerase, mitochondrial	Q13011	387978	426735	106717	349972	243077	397193	0.24

Supplementary Table 1		Relative protein quantitation (relative abundance units)						
		Subject #003	Subject #004	Subject #005	Subject #007	Subject #009	Subject #011	
	Fibrosis Score	3	0	2	1	4	0	Protein Relative Abundance vs Fibrosis Score (r^2)
Delta-1-pyrroline-5-carboxylate dehydrogenase, mitochondrial	P30038	331796	506202	452505	586798	232308	379346	0.50
Desmin	P17661	757962	179426	222131	344236	693997	334589	0.66
Dihydropyrimidinase	Q14117	186944	457016	319893	258023	165363	501282	0.82
Dimethylaniline monooxygenase [N-oxide-forming] 3	P31513	394972	303569	824179	461530	367279	176452	0.09
Dimethylglycine dehydrogenase, mitochondrial	Q9UI17	71098	204884	310757	166799	108610	172464	0.18
Dolichyl-diphosphooligosaccharide--protein glycosyltransferase subunit 1	P04843	449854	352575	344242	418752	167056	438193	0.33
Dolichyl-diphosphooligosaccharide--protein glycosyltransferase subunit 2	P04844	172761	201617	267661	304520	155656	301266	0.45
Electron transfer flavoprotein subunit alpha, mitochondrial	P13804	386707	290257	275701	266415	210552	319917	0.04
Electron transfer flavoprotein subunit beta	P38117	494636	694544	453915	649042	695915	580900	0.02
Electron transfer flavoprotein-ubiquinone oxidoreductase, mitochondrial	Q16134	141251	263238	254499	287683	136808	125776	0.17
Elongation factor 1-alpha 1	P68104	525964	340486	584660	398721	313436	248039	0.13
Elongation factor 2	P13639	250831	283020	330321	254603	131018	81463	0.00
Elongation factor Tu, mitochondrial	P49411	137925	154500	328492	379418	147467	455832	0.31
Endoplasmin	P14625	344267	369855	353882	329889	248518	365316	0.62
Enoyl-CoA delta isomerase 1, mitochondrial	P42126	195334	232074	274642	118133	109845	191030	0.11
Enoyl-CoA hydratase domain-containing protein 2, mitochondrial	Q86YB7	204058	415907	401002	278417	141470	161856	0.18
Enoyl-CoA hydratase, mitochondrial	P30084	610858	1112143	690096	610845	326055	473033	0.33

Supplementary Table 1

		Relative protein quantitation (relative abundance units)						
		Subject #003	Subject #004	Subject #005	Subject #007	Subject #009	Subject #011	
	Fibrosis Score	3	0	2	1	4	0	Protein Relative Abundance vs Fibrosis Score (r^2)
Epoxide hydrolase 1	P07099	654135	771135	704578	1275579	383199	555140	0.18
Epoxide hydrolase 2	P34913	243899	243298	437321	283429	162092	172393	0.00
Fatty acid synthase	P49327	235503	244420	196319	138753	161299	183450	0.02
Fatty acid-binding protein, liver	P07148	1254152	916977	1131374	672184	392193	1481518	0.21
Ferritin light chain	P02792	527382	848477	278869	771310	189208	1188323	0.76
Fibrinogen alpha chain	P02671	291943	148925	175322	151221	125799	173557	0.05
Fibronectin	P02751	254900	272894	107370	297530	224282	140261	0.00
Filamin-A	P21333	85578	147265	64499	217833	445665	109662	0.27
Flavin reductase (NADPH)	P30043	227722	372543	465491	335254	236524	331588	0.28
Formimidoyltransferase-cyclodeaminase	O95954	436453	349652	688029	518837	243495	388085	0.02
Fructose-1,6-bisphosphatase 1	P09467	711237	604075	711906	590495	258085	931007	0.42
Fructose-1,6-bisphosphatase isozyme 2	O00757	639445	893679	436424	433101	172263	711626	0.57
Fructose-bisphosphate aldolase A	P04075	186247	67871	265448	75813	176687	445526	0.02
Fructose-bisphosphate aldolase B	P05062	586162	771278	1180884	756997	379776	1364757	0.44
Fumarate hydratase, mitochondrial	P07954	216459	169188	205354	182483	217047	270764	0.00
Fumarylacetoacetase	P16930	393437	215734	319463	317627	211474	212186	0.10
Gamma-glutamyltransferase 5	P36269	185168	50131	141927	86105	377007	46163	0.88
Gelsolin	P06396	308953	551634	517407	450924	415235	480051	0.44
Glucose-6-phosphate isomerase	P06744	383323	275377	227165	202608	331686	350852	0.10
Glutamate dehydrogenase 1, mitochondrial	P00367	270121	568343	613558	461862	271996	563922	0.63
Glutathione S-transferase A1	P08263	574442	632373	867713	1005053	366771	498836	0.11
Glutathione S-transferase kappa 1	Q9Y2Q3	304799	353209	273203	203370	170263	289950	0.32
Glutathione S-transferase Mu 1	P09488	279812	228291	552558	368656	350683	764693	0.10
Glutathione S-transferase Mu 4	Q03013	108458	61081	324203	92904	84462	72348	0.04
Glutathione S-transferase omega-1	P78417	548357	642841	170320	426602	169090	381940	0.26

Supplementary Table 1

		Relative protein quantitation (relative abundance units)						
		Subject #003	Subject #004	Subject #005	Subject #007	Subject #009	Subject #011	Protein Relative Abundance vs Fibrosis Score (r^2)
	Fibrosis Score	3	0	2	1	4	0	
Glyceraldehyde-3-phosphate dehydrogenase	P04406	1380644	1170337	894214	783813	1177895	1335494	0.01
Glycine amidinotransferase, mitochondrial	P50440	184289	338702	279142	410511	185126	333337	0.71
Glycine dehydrogenase [decarboxylating], mitochondrial	P23378	81127	167082	380357	123061	328092	161041	0.15
Glycogen debranching enzyme	P35573	98264	265612	289618	178854	174994	136565	0.06
Glycogen phosphorylase, liver form	P06737	333794	211310	380734	281967	151228	241261	0.00
Glyoxylate reductase/hydroxypyruvate reductase	Q9UBQ7	510920	533438	821990	340740	205683	460226	0.07
GTP:AMP phosphotransferase, mitochondrial	Q9UIJ7	74992	327973	99384	131016	93293	124175	0.41
Heat shock 70 kDa protein 1A/1B	P08107	150358	155446	238416	170306	196951	180583	0.06
Heat shock cognate 71 kDa protein	P11142	438347	414764	220680	432117	529689	225657	0.28
Heat shock protein beta-1	P04792	492826	359815	267765	371582	964182	322955	0.59
Heat shock-related 70 kDa protein 2	P54652	556148	529909	156992	313833	335676	339085	0.00
Hemoglobin subunit alpha	P69905	2308345	2237914	2293501	1540541	2157804	795200	0.32
Hemoglobin subunit beta	P68871	4949829	8349896	4634742	6511228	9521177	840942	0.18
Hemoglobin subunit delta	P02042	416087	524363	323881	352702	568069	397942	0.08
Heterogeneous nuclear ribonucleoprotein K	P61978	309056	332536	279481	132729	298335	324611	0.00
Histone H1.1	Q02539	132381	69571	111903	336336	391912	264433	0.10
Histone H1.2	P16403	909240	83870	356725	348145	1036080	267101	0.89
Histone H2A type 1-A	Q96QV6	316017	273418	349390	353864	356543	238497	0.49
Histone H2A type 1-B/E	P04908	4238046	5868930	8319763	6297621	9881736	4136551	0.33
Histone H2A type 2-A	Q6FI13	425921	566413	348389	855773	570557	245626	0.00
Histone H2B type 1-A	Q96A08	1182246	7420451	678758	1451361	2249162	971255	0.14
Histone H2B type 1-B	P33778	1205545	949097	829574	777608	1297973	603589	0.70
Histone H3.1t	Q16695	2835253	1783906	2136936	4131110	3543674	1981725	0.24

Supplementary Table 1

	Fibrosis Score	Subject #003	Subject #004	Subject #005	Subject #007	Subject #009	Subject #011	Protein Relative Abundance vs Fibrosis Score (r^2)
		Relative protein quantitation (relative abundance units)						
		3	0	2	1	4	0	
Histone H3.3	P84243	1196440	1922767	4426890	2312006	3026133	2534041	0.02
Histone H4	P62805	477754	511143	677167	626834	881923	589060	0.32
HLA class I histocompatibility antigen, A-1 alpha chain	P30443	385334	483310	81563	295295	169066	246140	0.16
Homogentisate 1,2-dioxygenase	Q93099	125931	151050	150399	296300	101370	194141	0.32
Hydroxyacid oxidase 1	Q9UJM8	358219	463199	560663	443370	118680	499258	0.56
Hydroxyacyl-coenzyme A dehydrogenase, mitochondrial	Q16836	436489	663737	708468	347019	263779	660063	0.42
Hydroxymethylglutaryl-CoA synthase, cytoplasmic	Q01581	561899	807112	1309498	948830	951583	1249507	0.08
Hydroxymethylglutaryl-CoA synthase, mitochondrial	P54868	889906	805901	921317	1009559	380966	816608	0.31
Ig gamma-1 chain C region	P01857	594949	650478	366374	490347	2346445	333381	0.50
Ig kappa chain C region	P01834	907494	680757	229606	365814	2300271	418585	0.54
Ig kappa chain V-IV region Len	P01625	528091	652585	860040	455142	608480	1030181	0.16
Ig lambda-2 chain C regions	P0CG05	250485	334400	93896	160120	1023329	131636	0.43
Immunoglobulin lambda-like polypeptide 5	B9A064	518113	557161	147091	205781	1443485	88096	0.49
Isochorismatase domain-containing protein 2, mitochondrial	Q96AB3	435788	444997	755216	319952	245420	661910	0.17
Isocitrate dehydrogenase [NADP] cytoplasmic	O75874	187044	390294	277406	253201	163283	345636	0.87
Isocitrate dehydrogenase [NADP], mitochondrial	P48735	188002	221553	248502	333080	234604	259900	0.13
Keratin, type I cytoskeletal 10	P13645	173860	52025	128733	282718	127157	103590	0.04
Keratin, type I cytoskeletal 18	P05783	320065	362677	349298	321485	478609	260745	0.47
Keratin, type II cytoskeletal 1	P04264	263954	164678	180364	290400	272544	297854	0.03

Supplementary Table 1

		Relative protein quantitation (relative abundance units)						
		Subject #003	Subject #004	Subject #005	Subject #007	Subject #009	Subject #011	
	Fibrosis Score	3	0	2	1	4	0	Protein Relative Abundance vs Fibrosis Score (r^2)
Keratin, type II cytoskeletal 8	P05787	517904	379924	469837	730100	577197	303211	0.21
Liver carboxylesterase 1	P23141	429223	432849	1028001	539610	309484	938683	0.17
L-lactate dehydrogenase A chain	P00338	266267	212986	76863	164478	168385	233458	0.02
L-lactate dehydrogenase A-like 6B	Q9BYZ2	91251	288189	275476	188865	76797	501948	0.67
Long-chain-fatty-acid--CoA ligase 1	P33121	319105	273868	394015	351436	222037	376745	0.21
Lumican	P51884	354276	92826	192504	225709	1364902	156756	0.64
L-xylulose reductase	Q7Z4W1	209977	740233	1501310	785764	337978	933381	0.22
Malate dehydrogenase, cytoplasmic	P40925	369953	248507	276780	190024	310564	370791	0.07
Malate dehydrogenase, mitochondrial	P40926	674643	618158	397880	551420	412576	537876	0.11
Medium-chain specific acyl-CoA dehydrogenase, mitochondrial	P11310	276816	362594	499274	547578	198888	307125	0.19
Methylmalonate-semialdehyde dehydrogenase [acylating], mitochondrial	Q02252	214009	170819	338829	178651	123409	211277	0.01
Methyltransferase-like protein 7A	Q9H8H3	213444	226944	291388	289517	94370	265287	0.47
Microsomal glutathione S-transferase 1	P10620	305488	473622	480316	462509	1168122	439901	0.34
Microsomal triglyceride transfer protein large subunit	P55157	222743	237343	213733	218338	290980	136503	0.47
Mitochondrial carrier homolog 2	Q9Y6C9	141103	204958	266193	183383	160187	185162	0.12
MOSC domain-containing protein 2, mitochondrial	Q969Z3	199251	248491	400764	133183	73470	267834	0.16
Myomesin-2	P54296	122138	118515	62675	141653	166446	144186	0.02
Myosin light polypeptide 6	P60660	152264	109371	87981	148291	302028	176836	0.35
Myosin-1	P12882	144139	148675	148866	126291	155043	853283	0.24
Myosin-10	P35580	98278	188359	181543	193612	352707	110992	0.27
Myosin-11	P35749	133129	173074	111117	150203	399902	112616	0.40

Supplementary Table 1		Relative protein quantitation (relative abundance units)						
		Subject #003	Subject #004	Subject #005	Subject #007	Subject #009	Subject #011	
	Fibrosis Score	3	0	2	1	4	0	Protein Relative Abundance vs Fibrosis Score (r^2)
Myosin-2	Q9UKX2	319079	174041	182218	250918	120906	855377	0.25
Myosin-6	P13533	175503	159372	152535	170318	205518	1896148	0.23
Myosin-7	P12883	351774	67583	225849	159774	252354	1510719	0.13
Myosin-9	P35579	185963	161920	126915	193885	304824	175106	0.40
NAD kinase domain-containing protein 1	Q4G0N4	138704	219918	487972	364372	94097	104197	0.03
NAD(P) transhydrogenase, mitochondrial	Q13423	330472	379933	146101	493332	55110	111821	0.14
NADH dehydrogenase [ubiquinone] 1 alpha subcomplex subunit 13	Q9P0J0	228085	147347	34194	248503	94633	123338	0.01
NADPH--cytochrome P450 reductase	P16435	362088	339294	146749	314246	69072	121134	0.07
Neuroblast differentiation-associated protein AHNAK	Q09666	243745	488741	156684	148911	324724	367484	0.13
Neutral alpha-glucosidase AB	Q14697	272318	243840	377729	334901	430848	228709	0.55
Nucleophosmin	P06748	231399	222614	331409	202493	272443	272969	0.06
Omega-amidase NIT2	Q9NQR4	118702	212433	99852	216415	138769	264229	0.63
Ornithine carbamoyltransferase, mitochondrial	P00480	314963	389361	341659	229499	261620	281347	0.07
Peptidyl-prolyl cis-trans isomerase A	P62937	590983	747625	425151	674343	836311	602362	0.02
Peptidyl-prolyl cis-trans isomerase B	P23284	170275	233169	171629	209192	159437	200157	0.79
Peroxiredoxin-1	Q06830	1019936	666381	907221	735308	1107401	414530	0.88
Peroxiredoxin-2	P32119	269282	255307	135964	334085	302123	177410	0.07
Peroxiredoxin-4	Q13162	194149	445316	538884	150427	275059	363221	0.09
Peroxiredoxin-5, mitochondrial	P30044	183564	253353	374868	198616	135622	220625	0.12
Peroxiredoxin-6	P30041	490177	563437	728707	523567	450652	540395	0.09
Peroxisomal acyl-coenzyme A oxidase 2	Q99424	249716	349974	350169	476824	337004	533077	0.43
Peroxisomal bifunctional enzyme	Q08426	430661	446604	469253	465354	274039	298704	0.07

Supplementary Table 1

		Relative protein quantitation (relative abundance units)						
		Subject #003	Subject #004	Subject #005	Subject #007	Subject #009	Subject #011	
	Fibrosis Score	3	0	2	1	4	0	Protein Relative Abundance vs Fibrosis Score (r^2)
Peroxisomal multifunctional enzyme type 2	P51659	608784	507417	576479	729150	487969	648049	0.13
Peroxisomal sarcosine oxidase	Q9P0Z9	383050	235906	286224	311947	167149	287058	0.02
Phosphate carrier protein, mitochondrial	Q00325	252977	288114	225932	416625	184851	390159	0.56
Phosphatidylethanolamine-binding protein 1	P30086	767962	1131180	1553432	756538	589811	1454077	0.34
Phosphoenolpyruvate carboxykinase [GTP], mitochondrial	Q16822	378560	467212	887217	599282	244319	495337	0.13
Phosphoenolpyruvate carboxykinase, cytosolic [GTP]	P35558	457494	784118	918233	672141	158033	746207	0.59
Phosphoglucomutase-1	P36871	280233	375444	711595	194380	249171	422267	0.03
Phosphoglycerate kinase 1	P00558	343212	425896	371534	342606	525533	315115	0.30
Phosphoglycerate mutase 1	P18669	312973	324817	291884	221765	454533	315200	0.34
Phospholysine phosphohistidine inorganic pyrophosphate phosphatase	Q9H008	138974	147638	170379	104926	127039	149614	0.02
Plastin-2	P13796	385682	116196	227873	174940	490320	357454	0.51
Polypyrimidine tract-binding protein 1	P26599	72843	144719	360994	410094	169894	153720	0.02
Prelamin-A/C	P02545	188447	271218	235550	318499	388907	265579	0.05
Profilin-1	P07737	1210380	793256	928575	866264	1501847	988045	0.76
Prohibitin	P35232	241752	418077	340922	285458	241614	232957	0.21
Prohibitin-2	Q99623	373784	603661	418245	434138	231929	396791	0.64
Prolargin	P51888	156338	208072	527103	189125	640116	128846	0.45
Prostaglandin reductase 1	Q14914	458383	533179	300703	393300	331768	299576	0.05
Proteasome activator complex subunit 1	Q06323	248847	278340	309086	165819	256312	285677	0.00
Proteasome activator complex subunit 2	Q9UL46	418562	237898	98706	195806	107514	255705	0.01
Proteasome subunit alpha type-2	P25787	326604	217234	249857	213388	196737	226574	0.06
Protein disulfide-isomerase	P07237	856864	875122	1047673	762905	471495	900313	0.31

Supplementary Table 1

		Relative protein quantitation (relative abundance units)						
		Subject #003	Subject #004	Subject #005	Subject #007	Subject #009	Subject #011	
	Fibrosis Score	3	0	2	1	4	0	Protein Relative Abundance vs Fibrosis Score (r^2)
Protein disulfide-isomerase A3	P30101	363316	306944	264379	449013	190429	338278	0.25
Protein disulfide-isomerase A4	P13667	339980	405192	396269	305310	178474	161294	0.03
Protein disulfide-isomerase A6	Q15084	170102	444656	246133	258072	180730	244350	0.53
Protein DJ-1	Q99497	342033	170704	543689	492107	370948	197029	0.21
Protein NipSnap homolog 1	Q9BPW8	554620	339532	654851	538666	91258	494848	0.12
Protein-glutamine gamma-glutamyltransferase 2	P21980	280640	412463	281644	444373	346645	283366	0.09
Pterin-4-alpha-carbinolamine dehydratase	P61457	111669	71571	107521	142307	82137	66089	0.05
Putative beta-actin-like protein 3	Q9BYX7	3136398	1848144	1998178	3203406	7850359	4967053	0.27
Putative inactive carboxylesterase 4	Q9UKY3	673661	673716	904899	981529	290181	595623	0.20
Pyruvate carboxylase, mitochondrial	P11498	242388	516917	212188	251592	122564	283298	0.58
Pyruvate kinase isozymes M1/M2	P14618	192536	78096	96074	90694	356708	269119	0.27
Quinone oxidoreductase	Q08257	630240	803534	517238	560463	483693	341605	0.01
Rab GDP dissociation inhibitor alpha	P31150	275035	199342	232406	188225	195057	204269	0.14
Rab GDP dissociation inhibitor beta	P50395	317369	152820	267253	165559	130635	171181	0.08
Ras GTPase-activating-like protein IQGAP2	Q13576	52852	109293	82076	143967	60941	70354	0.31
Ras-related protein Rab-1A	P62820	97026	138450	88396	134877	138951	77030	0.02
Ras-related protein Rap-1A	P62834	190383	174240	187007	181260	339209	177705	0.58
Receptor expression-enhancing protein 6	Q96HR9	394313	270467	364846	246221	202868	289470	0.00
Retinal dehydrogenase 1	P00352	640764	1052662	794067	585992	703444	712443	0.18
Retinol dehydrogenase 16	O75452	404527	399300	819999	725077	178152	664770	0.27
Ribonuclease UK114	P52758	712584	871784	1101466	565073	198008	653361	0.21
S-adenosylmethionine synthase isoform type-1	Q00266	206731	221917	857294	218241	171718	385961	0.01
Sarcosine dehydrogenase, mitochondrial	Q9UL12	237901	146461	673861	219883	692150	227286	0.47

Supplementary Table 1

		Relative protein quantitation (relative abundance units)						
		Subject #003	Subject #004	Subject #005	Subject #007	Subject #009	Subject #011	
	Fibrosis Score	3	0	2	1	4	0	Protein Relative Abundance vs Fibrosis Score (r^2)
Selenium-binding protein 1	Q13228	269174	460120	478881	387427	331953	416338	0.43
Serine hydroxymethyltransferase, cytosolic	P34896	103419	242258	253714	246251	91124	252604	0.75
Serine--pyruvate aminotransferase	P21549	963371	775850	1248220	705912	361763	1041487	0.14
Serotransferrin	P02787	197550	223735	187065	235812	546312	186073	0.44
Serum albumin	P02768	1264608	843497	758360	742282	2074243	656875	0.73
Short/branched chain specific acyl-CoA dehydrogenase, mitochondrial	P45954	188899	571479	651389	303017	99599	364225	0.37
Short-chain specific acyl-CoA dehydrogenase, mitochondrial	P16219	238562	454766	424167	404246	158857	429138	0.84
Sideroflexin-1	Q9H9B4	421566	197220	409846	240995	148725	236493	0.03
Solute carrier family 2, facilitated glucose transporter member 2	P11168	109893	171615	126971	92580	58719	51605	0.09
Sorbitol dehydrogenase	Q00796	472964	240475	397337	438279	182375	203133	0.02
Spectrin alpha chain, brain	Q13813	150222	288942	105558	184258	142156	90319	0.10
Stress-70 protein, mitochondrial	P38646	286719	298779	142840	194284	133980	227072	0.21
Succinate dehydrogenase [ubiquinone] flavoprotein subunit, mitochondrial	P31040	346803	450447	266041	343266	146990	282984	0.45
Succinate-semialdehyde dehydrogenase, mitochondrial	P51649	108023	138238	165662	118327	74702	160060	0.52
Sulfotransferase 1A1	P50225	212486	288308	465995	459330	245442	528910	0.36
Superoxide dismutase [Cu-Zn]	P00441	457245	552857	922800	484957	232852	392405	0.05
Superoxide dismutase [Mn], mitochondrial	P04179	657785	883239	335770	510350	460642	347029	0.04
Talin-1	Q9Y490	384766	167009	259832	103707	329971	160789	0.72
Tetratricopeptide repeat protein 33	Q6PID6	174790	134341	190471	126723	297764	222060	0.37

Supplementary Table 1

		Relative protein quantitation (relative abundance units)						
		Subject #003	Subject #004	Subject #005	Subject #007	Subject #009	Subject #011	
	Fibrosis Score	3	0	2	1	4	0	Protein Relative Abundance vs Fibrosis Score (r^2)
Transforming growth factor-beta-induced protein ig-h3	Q15582	108640	75347	74142	109238	164131	82862	0.60
Thioredoxin-dependent peroxide reductase, mitochondrial	P30048	703891	653841	877673	649288	322639	752181	0.27
Thiosulfate sulfurtransferase	Q16762	517990	738458	839561	295008	233012	1013266	0.40
Thymidine phosphorylase	P19971	199934	249652	62457	141936	222649	166154	0.00
Titin	Q8WZ42	119069	112111	37581	142389	119531	166926	0.10
Transitional endoplasmic reticulum ATPase	P55072	190963	138936	116861	144239	139861	110615	0.22
Transketolase	P29401	279026	366654	137872	175814	280822	138339	0.02
Trifunctional enzyme subunit alpha, mitochondrial	P40939	266439	259370	488121	276743	232436	394676	0.07
Trifunctional enzyme subunit beta, mitochondrial	P55084	579074	621268	149764	733928	224247	244328	0.08
Triosephosphate isomerase	P60174	518834	457509	568470	458353	446480	495912	0.01
Tripeptidyl-peptidase 1	O14773	323319	649561	737563	308494	411842	228362	0.00
Tropomyosin alpha-1 chain	P09493	547638	563362	778518	455018	557770	817380	0.07
Tubulin alpha-1A chain	Q71U36	859939	674352	590628	440398	703606	511317	0.34
Tubulin alpha-1B chain	P68363	1709862	1289687	719981	912061	1146304	921777	0.10
Tubulin beta chain	P07437	261355	506499	180804	381288	430937	545378	0.26
Tubulin beta-1 chain	Q9H4B7	473291	585317	179866	191000	636882	759175	0.01
Tubulin beta-2A chain	Q13885	403802	439371	247221	354059	461813	243764	0.18
Tubulin beta-3 chain	Q13509	166022	422122	349708	176775	142994	163797	0.19
Tubulin beta-4A chain	P04350	219196	219081	287775	158776	216158	151416	0.17
Type I inositol-3,4-bisphosphate 4-phosphatase	Q96PE3	341799	369389	385697	313398	335283	385792	0.17

Supplementary Table 1

		Relative protein quantitation (relative abundance units)						
		Subject #003	Subject #004	Subject #005	Subject #007	Subject #009	Subject #011	
	Fibrosis Score	3	0	2	1	4	0	Protein Relative Abundance vs Fibrosis Score (r^2)
Ubiquitin-60S ribosomal protein L40	P62987	1449660	1139230	1014710	932848	1862468	1157719	0.60
UDP-glucose 6-dehydrogenase	O60701	180664	432347	228292	228194	86757	234822	0.65
UDP-glucuronosyltransferase 1-1	P22309	255065	694079	136377	149674	216184	235772	0.20
UDP-glucuronosyltransferase 1-10	Q9HAW8	212468	713681	408846	248535	142542	514565	0.69
UDP-glucuronosyltransferase 1-4	P22310	765404	566843	605745	254091	219322	242903	0.01
UDP-glucuronosyltransferase 2B4	P06133	546673	497816	335606	217334	296539	305526	0.00
UDP-glucuronosyltransferase 2B7	P16662	417669	409813	539230	200338	340375	171213	0.14
UTP--glucose-1-phosphate uridylyltransferase	Q16851	475766	501346	536491	304878	274912	492072	0.20
Very long-chain acyl-CoA synthetase	O14975	205157	342755	223850	265818	120463	339131	0.97
Very long-chain specific acyl-CoA dehydrogenase, mitochondrial	P49748	186587	179784	148026	208817	126272	151288	0.16
Vimentin	P08670	457354	352157	259116	313064	1242378	255864	0.58
Vinculin	P18206	137409	96949	51386	90821	517914	89404	0.52
Voltage-dependent anion-selective channel protein 1	P21796	288950	209133	93334	477319	531222	432717	0.04
Xanthine dehydrogenase/oxidase	P47989	119484	137317	112105	60063	171274	188489	0.00
Zinc finger protein 891	A8MT65	79656	46911	39968	53149	136749	160549	0.02
