# Supplementary material for: Turnover Rates of Hepatic Collagen and Circulating Collagen-Associated Proteins in Humans with Chronic Liver Disease
Source: PLoS One. 2015 Apr 24;10(4):e0123311. doi: 10.1371/journal.pone.0123311 (PMC4409311; doi:10.1371/journal.pone.0123311)
Supplement: S2 Table — (PDF) [file pone.0123311.s003.pdf]

**Supplementary Table 2**

**Total fraction new at end of labeleing period - % new (F)**

| Liver Proteins | Uniprot Accession | Subject #003 | Subject #004 | Subject #005 | Subject #007 | Subject #009 | Subject #011 |
|---|---|---|---|---|---|---|---|
| | Fibrosis Score | 3 | 0 | 2 | 1 | 4 | 0 |
| 1,4-alpha-glucan-branching enzyme | Q04446 | | | 30.88% | | | |
| 14-3-3 protein epsilon | P62258 | | | | | | 72.43% |
| 14-3-3 protein eta | Q04917 | | | | | 34.39% | 36.91% |
| 17-beta-hydroxysteroid dehydrogenase 13 | Q7Z5P4 | | 75.91% | 79.19% | 97.87% | | 74.13% |
| 17-beta-hydroxysteroid dehydrogenase type 6 | O14756 | | | 62.68% | 74.18% | | 73.86% |
| 2,4-dienoyl-CoA reductase, mitochondrial | Q16698 | 93.15% | 51.27% | 51.38% | 84.89% | | 64.27% |
| 3-hydroxyacyl-CoA dehydratase 3 | Q9P035 | | | 57.24% | | | |
| 3-hydroxyacyl-CoA dehydrogenase type-2 | Q99714 | 52.52% | 42.53% | 42.53% | 52.36% | | 47.27% |
| 3-hydroxyanthranilate 3,4-dioxygenase | P46952 | | | 40.76% | | | |
| 3-ketoacyl-CoA thiolase, mitochondrial | P42765 | 81.48% | 46.94% | 45.07% | 78.16% | 79.03% | 53.81% |
| 3-ketoacyl-CoA thiolase, peroxisomal | P09110 | 100.67% | 76.45% | 67.06% | 84.66% | 90.61% | 68.38% |
| 3-mercaptopyruvate sulfurtransferase | P25325 | 95.52% | 57.54% | 54.17% | 78.49% | | 58.36% |
| 40S ribosomal protein S7 | P62081 | | 70.49% | 62.69% | 89.55% | | 68.47% |
| 40S ribosomal protein S8 | P62241 | 23.12% | 5.75% | 5.39% | 36.76% | 31.51% | 8.73% |
| 40S ribosomal protein SA | P08865 | 86.45% | 54.32% | 52.22% | 74.14% | 71.14% | 54.13% |
| 4-aminobutyrate aminotransferase, mitochondrial | P80404 | 90.42% | 58.38% | 48.13% | 76.79% | 94.46% | 59.39% |
| 4-hydroxyphenylpyruvate dioxygenase | P32754 | | 91.59% | 77.97% | | | 77.07% |
| 4-trimethylaminobutyraldehyde dehydrogenase | P49189 | | 46.09% | 44.96% | | | |
| 60 kDa heat shock protein, mitochondrial | P10809 | 87.05% | 50.39% | 45.29% | 73.48% | | 53.91% |
| 60S acidic ribosomal protein P0 | P05388 | | | 52.12% | | | |
| 60S ribosomal protein L6 | Q02878 | 79.22% | 51.32% | 51.69% | 68.00% | 55.20% | 53.24% |
| 60S ribosomal protein L7 | P18124 | 95.32% | | | | | |
| 78 kDa glucose-regulated protein | P11021 | 102.49% | 76.33% | 62.58% | 87.18% | 96.40% | 79.34% |
| Abhydrolase domain-containing protein 14B | Q96IU4 | | 77.95% | | | | |

**Supplementary Table 2** | **Total fraction new at end of labeleing period - % new (F)**

| | | **Subject #003** | **Subject #004** | **Subject #005** | **Subject #007** | **Subject #009** | **Subject #011** |
|---|---|---|---|---|---|---|---|
| | **Fibrosis Score** | **3** | **0** | **2** | **1** | **4** | **0** |
| Acetyl-CoA acetyltransferase, cytosolic | Q9BWD1 | | 51.96% | 52.67% | 67.44% | | 80.75% |
| Acetyl-CoA acetyltransferase, mitochondrial | P24752 | 83.72% | 52.77% | 41.38% | 78.63% | 70.53% | 49.96% |
| Aconitate hydratase, mitochondrial | Q99798 | | 70.22% | | | | |
| Actin, alpha skeletal muscle | P68133 | | | | | | 9.48% |
| Actin, aortic smooth muscle | P62736 | 93.33% | 50.79% | 48.98% | 72.21% | 37.71% | 12.67% |
| Actin, cytoplasmic 1 | P60709 | 84.88% | 55.94% | 49.63% | 74.02% | 51.79% | 54.80% |
| Acyl-coenzyme A synthetase ACSM2A, mitochondrial | Q08AH3 | | 55.43% | 53.26% | | | |
| Acyl-coenzyme A thioesterase 1 | Q86TX2 | | 60.49% | | | | 57.38% |
| Adenylate kinase 2, mitochondrial | P54819 | | 45.40% | 50.51% | | | |
| ADP/ATP translocase 1 | P12235 | | 54.57% | 68.90% | 85.58% | | 54.53% |
| Aflatoxin B1 aldehyde reductase member 3 | O95154 | | 76.54% | | | | |
| Agmatinase, mitochondrial | Q9BSE5 | | | 33.69% | | | |
| Alanine aminotransferase 1 | P24298 | | | 28.31% | | | |
| Alcohol dehydrogenase [NADP+] | P14550 | | | 68.08% | | | |
| Alcohol dehydrogenase 1A | P07327 | 95.49% | 59.93% | 43.79% | 78.37% | 70.39% | 56.61% |
| Alcohol dehydrogenase 1B | P00325 | 95.13% | 58.48% | 40.30% | 74.17% | 84.42% | 57.70% |
| Alcohol dehydrogenase 1C | P00326 | 96.31% | 61.22% | | 84.16% | | 59.47% |
| Alcohol dehydrogenase 4 | P08319 | 100.36% | 61.47% | 46.73% | 76.33% | 86.49% | 61.52% |
| Alcohol dehydrogenase 6 | P28332 | | 46.82% | 48.70% | 70.01% | | 55.40% |
| Alcohol dehydrogenase class-3 | P11766 | | 46.93% | 32.23% | | | |
| Aldehyde dehydrogenase family 8 member A1 | Q9H2A2 | | 70.12% | 55.80% | | | 60.83% |
| Aldehyde dehydrogenase X, mitochondrial | P30837 | 100.77% | 61.21% | | 96.02% | 96.53% | 61.46% |
| Aldehyde dehydrogenase, mitochondrial | P05091 | 101.68% | 68.33% | 60.89% | 83.66% | 99.04% | 69.34% |
| Aldehyde oxidase | Q06278 | | 70.58% | 72.00% | 84.03% | | 65.25% |
| Aldo-keto reductase family 1 member C1 | Q04828 | | 63.01% | 46.66% | 68.73% | 88.52% | 55.57% |
| Aldo-keto reductase family 1 member C3 | P42330 | | | 47.71% | | | |
| Aldo-keto reductase family 1 member C4 | P17516 | | | 49.18% | | | |
| Alpha-1-antitrypsin | P01009 | | 75.45% | | | 93.00% | 86.47% |

**Supplementary Table 2**

**Total fraction new at end of labeleing period - % new (F)**

| | | Subject #003 | Subject #004 | Subject #005 | Subject #007 | Subject #009 | Subject #011 |
|---|---|---|---|---|---|---|---|
| | **Fibrosis Score** | **3** | **0** | **2** | **1** | **4** | **0** |
| Alpha-2-macroglobulin | P01023 | 85.17% | 62.74% | 54.75% | 71.62% | 73.84% | 51.44% |
| Alpha-actinin-1 | P12814 | 95.48% | 73.65% | | 84.87% | 68.06% | 38.76% |
| Alpha-actinin-2 | P35609 | | | | | | 22.31% |
| Alpha-actinin-4 | O43707 | 83.31% | 58.67% | 57.48% | | 58.97% | 48.70% |
| Alpha-enolase | P06733 | 101.49% | 65.36% | 42.36% | 83.52% | 98.20% | 70.52% |
| Amine oxidase [flavin-containing] A | P21397 | | 45.47% | 44.71% | 68.98% | | |
| Amine oxidase [flavin-containing] B | P27338 | 99.60% | 47.94% | 26.88% | 82.90% | | |
| Aminoacylase-1 | Q03154 | | 59.00% | 50.50% | 71.90% | 96.25% | 66.55% |
| Annexin A1 | P04083 | | | | | 69.27% | |
| Annexin A2 | P07355 | | 58.01% | | | 55.55% | 49.55% |
| Annexin A4 | P09525 | | | | | 89.70% | |
| Annexin A5 | P08758 | | 65.23% | 64.06% | 82.47% | 77.25% | 60.28% |
| Annexin A6 | P08133 | 98.52% | 76.68% | 72.19% | 96.40% | 88.56% | 70.84% |
| Apolipoprotein A-I | P02647 | | | | | 73.97% | |
| Apoptosis-inducing factor 1, mitochondrial | O95831 | | 42.18% | 27.52% | 65.88% | | 39.30% |
| Arginase-1 | P05089 | 87.95% | 54.91% | 44.15% | 72.12% | | 57.40% |
| Argininosuccinate lyase | P04424 | 83.63% | 56.97% | 57.44% | 65.16% | 75.70% | 57.40% |
| Argininosuccinate synthase | P00966 | | 66.41% | 50.21% | 75.61% | | 73.25% |
| Arylacetamide deacetylase | P22760 | | | | | | 77.43% |
| Aspartate aminotransferase, cytoplasmic | P17174 | 98.70% | 63.08% | 53.48% | 83.87% | | 57.55% |
| Aspartate aminotransferase, mitochondrial | P00505 | 81.36% | 49.94% | 43.66% | 73.66% | 71.63% | 46.22% |
| ATP synthase subunit alpha, mitochondrial | P25705 | 100.10% | 57.25% | 52.56% | 85.48% | 75.65% | 57.08% |
| ATP synthase subunit b, mitochondrial | P24539 | | 45.19% | | 71.06% | | |
| ATP synthase subunit beta, mitochondrial | P06576 | 92.60% | 48.87% | 41.53% | 81.60% | 80.44% | 49.27% |
| ATP synthase subunit delta, mitochondrial | P30049 | | | | 81.37% | | |
| ATP synthase subunit f, mitochondrial | P56134 | | 43.11% | | | | |
| ATP synthase subunit O, mitochondrial | P48047 | 86.82% | 42.31% | 71.45% | 58.51% | | 59.94% |
| ATP-binding cassette sub-family D member 3 | P28288 | | 64.73% | 57.52% | 81.42% | | 76.65% |
| Beta-actin-like protein 2 | Q562R1 | | | 39.83% | | | |

**Supplementary Table 2**

**Total fraction new at end of labeleing period - % new (F)**

| | | Subject #003 | Subject #004 | Subject #005 | Subject #007 | Subject #009 | Subject #011 |
|---|---|---|---|---|---|---|---|
| | **Fibrosis Score** | **3** | **0** | **2** | **1** | **4** | **0** |
| Beta-enolase | P13929 | | | | | | 52.95% |
| Betaine--homocysteine S-methyltransferase 1 | Q93088 | 98.02% | 64.46% | 54.88% | 80.63% | | 64.57% |
| Betaine--homocysteine S-methyltransferase 2 | Q9H2M3 | | | 59.24% | | | 51.03% |
| Bifunctional ATP-dependent dihydroxyacetone kinase/FAD-AMP lyase (cyclizing) | Q3LXA3 | 90.94% | 57.04% | 54.42% | 71.57% | | 57.30% |
| Biglycan | P21810 | | | | | 32.65% | |
| Bile acid-CoA:amino acid N-acyltransferase | Q14032 | | 59.23% | 41.37% | | | 73.80% |
| Bile acyl-CoA synthetase | Q9Y2P5 | | 74.76% | 57.47% | 85.30% | | 70.56% |
| Bile salt sulfotransferase | Q06520 | 98.88% | 62.24% | 54.68% | 83.23% | | 64.58% |
| C-1-tetrahydrofolate synthase, cytoplasmic | P11586 | 72.35% | 51.57% | 41.35% | 58.76% | | 58.65% |
| Calcium-binding mitochondrial carrier protein Aralar1 | O75746 | | 46.85% | | 63.14% | | 50.34% |
| Calcium-binding mitochondrial carrier protein Aralar2 | Q9UJS0 | 86.73% | 46.01% | 34.66% | 75.48% | | 53.02% |
| Caldesmon | Q05682 | | | | | 81.10% | |
| Calmodulin | P62158 | | | | | 95.70% | 89.22% |
| Calnexin | P27824 | 103.89% | 74.55% | 64.24% | | | 70.29% |
| Calreticulin | P27797 | 109.41% | 66.82% | 61.52% | 75.87% | 84.77% | 71.89% |
| Calsequestrin-1 | P31415 | | | | | | 15.37% |
| Carbamoyl-phosphate synthase [ammonia], mitochondrial | P31327 | 87.63% | 47.79% | 41.85% | 77.01% | 80.08% | 51.03% |
| Carbonic anhydrase 1 | P00915 | | | 26.51% | | 58.57% | |
| Carbonic anhydrase 2 | P00918 | 89.24% | 57.72% | 45.82% | 75.87% | 73.82% | 55.03% |
| Carbonic anhydrase 3 | P07451 | | | | 87.41% | | 22.35% |
| Carbonyl reductase [NADPH] 1 | P16152 | 93.21% | 59.10% | 52.55% | 72.11% | 77.01% | 58.59% |
| Catalase | P04040 | 97.82% | 66.35% | 65.82% | 83.43% | 81.49% | 70.16% |
| Cathepsin D | P07339 | 103.88% | 68.31% | 58.14% | 86.91% | 92.44% | 74.94% |
| Clathrin heavy chain 1 | Q00610 | | | | | | 65.23% |
| Cocaine esterase | O00748 | | 47.67% | 34.09% | | | |

**Supplementary Table 2**

**Total fraction new at end of labeleing period - % new (F)**

| | | **Subject #003** | **Subject #004** | **Subject #005** | **Subject #007** | **Subject #009** | **Subject #011** |
|---|---|---|---|---|---|---|---|
| | **Fibrosis Score** | **3** | **0** | **2** | **1** | **4** | **0** |
| Cofilin-1 | P23528 | | | 28.58% | | 79.22% | |
| Collagen alpha-1(I) chain | P02452 | 18.65% | 4.26% | 7.41% | 12.13% | 11.66% | 6.41% |
| Collagen alpha-1(III) chain | P02461 | 17.60% | 5.28% | 4.68% | 9.37% | 8.77% | 6.69% |
| Collagen alpha-1(IV) chain | P02462 | | | 1.81% | | 18.48% | |
| Collagen alpha-1(VI) chain | P12109 | 34.81% | 16.54% | 17.33% | 34.35% | 22.13% | 16.80% |
| Collagen alpha-1(XIV) chain | Q05707 | | | | | 38.24% | |
| Collagen alpha-2(I) chain | P08123 | 19.58% | 8.65% | 9.37% | 12.68% | 11.59% | 8.35% |
| Collagen alpha-3(VI) chain | P12111 | 28.21% | 24.51% | 12.37% | 25.41% | 18.63% | 19.25% |
| Complement C3 | P01024 | | 97.13% | | 81.81% | | |
| Complement C4-A | P0C0L4 | | 82.13% | | | | |
| Corticosteroid 11-beta-dehydrogenase isozyme 1 | P28845 | | 78.97% | | | | |
| Creatine kinase M-type | P06732 | | | | | | 16.36% |
| Creatine kinase S-type, mitochondrial | P17540 | | | | | | 39.94% |
| Cullin-associated NEDD8-dissociated protein 1 | Q86VP6 | | 50.56% | 47.48% | | | |
| Cystathionine gamma-lyase | P32929 | 107.46% | 75.44% | 55.98% | 96.23% | | 66.98% |
| Cytochrome b5 | P00167 | 106.58% | 65.41% | 57.12% | 84.83% | 89.46% | 67.84% |
| Cytochrome b-c1 complex subunit 2, mitochondrial | P22695 | | | | | | 57.17% |
| Cytochrome c oxidase subunit 2 | P00403 | 85.35% | 57.55% | 43.35% | 91.81% | 81.64% | 55.12% |
| Cytochrome c oxidase subunit 5A, mitochondrial | P20674 | | 42.35% | 34.92% | 65.14% | | 41.99% |
| Cytochrome P450 1A2 | P05177 | | 73.40% | | 65.12% | | |
| Cytochrome P450 2A6 | P11509 | | 101.36% | | | | |
| Cytochrome P450 2C8 | P10632 | | 97.51% | 101.84% | | | |
| Cytochrome P450 2C9 | P11712 | 94.02% | 83.99% | 63.08% | 86.00% | | 80.84% |
| Cytochrome P450 2E1 | P05181 | | | | 85.61% | | |
| Cytochrome P450 3A4 | P08684 | | | 75.74% | | | |
| Cytochrome P450 3A5 | P20815 | | 76.90% | | 75.72% | 93.98% | 77.39% |

**Supplementary Table 2**

**Total fraction new at end of labeleing period - % new (F)**

| | | **Subject #003** | **Subject #004** | **Subject #005** | **Subject #007** | **Subject #009** | **Subject #011** |
|---|---|---|---|---|---|---|---|
| | **Fibrosis Score** | **3** | **0** | **2** | **1** | **4** | **0** |
| Cytoplasmic aconitate hydratase | P21399 | | 61.12% | 52.86% | 81.05% | | |
| Cytosol aminopeptidase | P28838 | | 67.20% | | | | 74.02% |
| Cytosolic 10-formyltetrahydrofolate dehydrogenase | O75891 | | 53.49% | 60.92% | 93.43% | | 57.97% |
| Cytosolic non-specific dipeptidase | Q96KP4 | 70.97% | 57.18% | 31.06% | | | 80.80% |
| D-beta-hydroxybutyrate dehydrogenase, mitochondrial | Q02338 | 96.54% | 48.47% | 57.34% | | | 52.22% |
| D-dopachrome decarboxylase | P30046 | | 60.38% | 47.92% | 81.59% | 81.27% | 60.69% |
| Delta(3,5)-Delta(2,4)-dienoyl-CoA isomerase, mitochondrial | Q13011 | 88.13% | 49.83% | | 75.09% | 73.56% | 57.22% |
| Delta-1-pyrroline-5-carboxylate dehydrogenase, mitochondrial | P30038 | 92.90% | 55.33% | 50.84% | 83.61% | 49.54% | 59.35% |
| Delta-aminolevulinic acid dehydratase | P13716 | | | 37.26% | | | |
| Desmin | P17661 | | | | | 61.67% | 61.98% |
| Dihydropyrimidinase | Q14117 | | | 62.10% | 91.70% | | 70.09% |
| Dimethylaniline monooxygenase [N-oxide-forming] 3 | P31513 | | 69.67% | 55.11% | 77.93% | | |
| Dimethylglycine dehydrogenase, mitochondrial | Q9UI17 | | 48.99% | 62.73% | | | |
| Dolichyl-diphosphooligosaccharide--protein glycosyltransferase subunit 1 | P04843 | 89.46% | 50.88% | | 70.20% | | |
| Dolichyl-diphosphooligosaccharide--protein glycosyltransferase subunit 2 | P04844 | | 65.99% | 54.69% | 75.44% | | 55.86% |
| Elastin | P15502 | | | | | 7.86% | |
| Electron transfer flavoprotein subunit alpha, mitochondrial | P13804 | 89.84% | 49.58% | 46.01% | 75.94% | 77.93% | 49.71% |
| Electron transfer flavoprotein subunit beta | P38117 | 67.15% | 48.44% | 42.74% | 85.22% | | 59.50% |
| Electron transfer flavoprotein-ubiquinone oxidoreductase, mitochondrial | Q16134 | | 68.59% | 59.55% | | | 76.79% |

**Supplementary Table 2**

**Total fraction new at end of labeleing period - % new (F)**

| | | Subject #003 | Subject #004 | Subject #005 | Subject #007 | Subject #009 | Subject #011 |
|---|---|---|---|---|---|---|---|
| | Fibrosis Score | 3 | 0 | 2 | 1 | 4 | 0 |
| Elongation factor 1-alpha 1 | P68104 | 90.45% | 61.96% | 55.87% | 69.03% | 77.40% | 55.06% |
| Elongation factor 2 | P13639 | | 85.18% | 75.04% | | | |
| Endoplasmin | P14625 | 102.60% | 73.35% | 65.52% | 87.97% | 95.42% | 77.69% |
| Enoyl-CoA hydratase domain-containing protein 2, mitochondrial | Q86YB7 | | 53.38% | 58.12% | 96.52% | | 80.40% |
| Enoyl-CoA hydratase, mitochondrial | P30084 | 86.48% | 44.92% | 41.81% | 72.24% | 72.59% | 48.89% |
| Epoxide hydrolase 1 | P07099 | 98.15% | 63.71% | 52.81% | 82.49% | 86.46% | 66.28% |
| Epoxide hydrolase 2 | P34913 | | 67.44% | 52.54% | 80.70% | | 73.63% |
| Ester hydrolase C11orf54 | Q9H0W9 | | 54.78% | 67.96% | | | |
| Estradiol 17-beta-dehydrogenase 11 | Q8NBQ5 | | | 62.89% | | | |
| Fatty acid synthase | P49327 | 105.87% | 86.59% | 91.38% | 79.43% | | 82.10% |
| Fatty acid-binding protein, liver | P07148 | 93.26% | 73.50% | 47.63% | 78.45% | 83.61% | 59.10% |
| Ferritin light chain | P02792 | 95.31% | 70.40% | 77.45% | 83.76% | 97.61% | 65.16% |
| Fibronectin | P02751 | 104.52% | 60.85% | | | 81.85% | 80.44% |
| Filamin-A | P21333 | | | | 108.52% | 53.81% | 80.16% |
| Filamin-B | O75369 | | | 60.70% | | | |
| Filamin-C | Q14315 | | | | | | 26.46% |
| Flavin reductase (NADPH) | P30043 | | 53.46% | 54.53% | 69.77% | | 65.26% |
| Formimidoyltransferase-cyclodeaminase | O95954 | 91.20% | 62.17% | 54.44% | 80.74% | 87.14% | 59.95% |
| Fructose-1,6-bisphosphatase 1 | P09467 | 104.22% | 68.29% | 52.19% | 86.82% | 80.55% | 65.05% |
| Fructose-bisphosphate aldolase A | P04075 | | | | | | 42.13% |
| Fructose-bisphosphate aldolase B | P05062 | 111.24% | 77.15% | 65.90% | 84.37% | 95.51% | 74.32% |
| Fumarate hydratase, mitochondrial | P07954 | | 50.49% | 45.47% | 76.69% | | 51.00% |
| Fumarylacetoacetase | P16930 | 89.91% | 56.58% | 42.86% | 71.22% | 69.76% | 50.09% |
| Galactokinase | P51570 | | 50.23% | 42.12% | | | 51.13% |
| Galectin-1 | P09382 | | | | | 58.98% | 61.11% |
| Gamma-glutamyltransferase 5 | P36269 | 98.64% | | 58.23% | | 60.85% | |
| Gelsolin | P06396 | | 57.90% | 64.72% | | | |
| Glucose-6-phosphate isomerase | P06744 | 77.30% | 52.15% | 45.38% | 75.83% | 72.33% | 50.52% |

**Supplementary Table 2**

**Total fraction new at end of labeleing period - % new (F)**

| | Fibrosis Score | Subject #003 | Subject #004 | Subject #005 | Subject #007 | Subject #009 | Subject #011 |
|---|---|---|---|---|---|---|---|
| | | 3 | 0 | 2 | 1 | 4 | 0 |
| Glutamate dehydrogenase 1, mitochondrial | P00367 | 87.26% | 43.79% | 38.33% | 76.89% | | 43.42% |
| Glutathione S-transferase A1 | P08263 | 93.75% | 58.45% | 41.51% | 74.62% | 86.92% | 57.54% |
| Glutathione S-transferase kappa 1 | Q9Y2Q3 | | 67.96% | | | | 74.51% |
| Glutathione S-transferase Mu 1 | P09488 | 91.49% | | 52.38% | 65.98% | | 65.08% |
| Glutathione S-transferase omega-1 | P78417 | | | 35.67% | | | |
| Glyceraldehyde-3-phosphate dehydrogenase | P04406 | 95.24% | 59.62% | 50.23% | 79.11% | 64.22% | 50.29% |
| Glycerol-3-phosphate dehydrogenase [NAD+], cytoplasmic | P21695 | | 60.22% | 46.27% | | | |
| Glycine amidinotransferase, mitochondrial | P50440 | 104.05% | 69.98% | 68.17% | 81.68% | 90.24% | 66.90% |
| Glycine dehydrogenase [decarboxylating], mitochondrial | P23378 | | | 15.99% | | | |
| Glycogen debranching enzyme | P35573 | | | 19.89% | | | 35.83% |
| Glycogen phosphorylase, brain form | P11216 | | | | | | 37.80% |
| Glycogen phosphorylase, liver form | P06737 | 75.62% | 46.99% | 30.22% | 60.46% | | 32.21% |
| Glycogen phosphorylase, muscle form | P11217 | | | | | | 26.70% |
| Glyoxylate reductase/hydroxypyruvate reductase | Q9UBQ7 | 87.44% | 56.97% | 46.20% | 73.77% | 75.96% | 55.26% |
| Heat shock 70 kDa protein 1A/1B | P08107 | | 73.34% | 69.27% | | 72.62% | 59.46% |
| Heat shock cognate 71 kDa protein | P11142 | | 80.69% | | 89.96% | | |
| Heat shock protein beta-1 | P04792 | 99.56% | 69.28% | 63.57% | 80.61% | 76.66% | 58.63% |
| Heat shock-related 70 kDa protein 2 | P54652 | | | 59.33% | 88.25% | | 82.67% |
| Hemoglobin subunit alpha | P69905 | 30.42% | 10.13% | 12.24% | 37.10% | 40.45% | 11.75% |
| Hemoglobin subunit beta | P68871 | 29.17% | 9.63% | 6.63% | 39.31% | 36.81% | 14.35% |
| Hemoglobin subunit delta | P02042 | 39.52% | 20.27% | 25.79% | 54.39% | 51.01% | 21.29% |
| Hemoglobin subunit gamma-1 | P69891 | | | 15.67% | | | |
| Heterogeneous nuclear ribonucleoprotein K | P61978 | 101.01% | 77.56% | 88.93% | | 97.46% | |
| Histone H2A type 1-A | Q96QV6 | | 35.68% | 24.45% | 51.89% | 30.27% | 28.63% |
| Histone H2A type 1-B/E | P04908 | 54.67% | 26.91% | 10.42% | 41.99% | 24.35% | 20.52% |
| Histone H2B type 1-A | Q96A08 | | | | | 25.82% | |

**Supplementary Table 2** **Total fraction new at end of labeleing period - % new (F)**

| | | Subject #003 | Subject #004 | Subject #005 | Subject #007 | Subject #009 | Subject #011 |
|---|---|---|---|---|---|---|---|
| | Fibrosis Score | 3 | 0 | 2 | 1 | 4 | 0 |
| Histone H2B type 1-B | P33778 | 54.94% | 27.23% | 17.79% | 47.63% | 21.19% | 23.46% |
| Histone H3.1t | Q16695 | 29.73% | 18.92% | 6.35% | 22.39% | 11.72% | 9.91% |
| Histone H3.3 | P84243 | 32.61% | 12.52% | 15.03% | 20.92% | 11.14% | 14.41% |
| Histone H4 | P62805 | 44.46% | 19.22% | 13.97% | 34.38% | 21.86% | 18.99% |
| HLA class I histocompatibility antigen, A-1 alpha chain | P30443 | 104.57% | | | | | |
| HLA class I histocompatibility antigen, A-25 alpha chain | P18462 | 106.86% | | | | | |
| Homogentisate 1,2-dioxygenase | Q93099 | | | 39.83% | 95.44% | | 75.36% |
| Hydroxyacid oxidase 1 | Q9UJM8 | 108.55% | 74.86% | 75.82% | 95.38% | | 73.12% |
| Hydroxyacyl-coenzyme A dehydrogenase, mitochondrial | Q16836 | 89.11% | 43.51% | 40.08% | 72.34% | 62.53% | 43.46% |
| Hydroxymethylglutaryl-CoA synthase, cytoplasmic | Q01581 | | | 62.74% | 94.04% | | 73.28% |
| Hydroxymethylglutaryl-CoA synthase, mitochondrial | P54868 | 95.07% | 65.52% | 62.92% | 84.23% | 98.47% | 64.91% |
| Ig alpha-1 chain C region | P01876 | | | | | 73.25% | |
| Ig gamma-1 chain C region | P01857 | 67.12% | 57.70% | 18.18% | 63.15% | 48.30% | 47.34% |
| Ig gamma-2 chain C region | P01859 | | | | | 41.73% | |
| Ig heavy chain V-III region BRO | P01766 | | | | | 59.41% | |
| Ig kappa chain C region | P01834 | 63.03% | 52.24% | 27.84% | 49.05% | 47.88% | 33.83% |
| Ig lambda-2 chain C regions | P0CG05 | | | | | 57.67% | |
| Immunoglobulin lambda-like polypeptide 5 | B9A064 | | 56.71% | | | 61.82% | |
| Isochorismatase domain-containing protein 2, mitochondrial | Q96AB3 | 77.79% | 40.66% | 35.87% | 44.40% | | 47.26% |
| Isocitrate dehydrogenase [NADP] cytoplasmic | O75874 | 110.19% | 56.22% | 51.49% | 80.30% | | 51.94% |
| Isocitrate dehydrogenase [NADP], mitochondrial | P48735 | 80.92% | 35.20% | 43.45% | | 61.04% | 43.95% |
| Keratin, type I cytoskeletal 10 | P13645 | | | | 11.99% | | |

**Supplementary Table 2**

**Total fraction new at end of labeleing period - % new (F)**

| | | **Subject #003** | **Subject #004** | **Subject #005** | **Subject #007** | **Subject #009** | **Subject #011** |
|---|---|---|---|---|---|---|---|
| | **Fibrosis Score** | **3** | **0** | **2** | **1** | **4** | **0** |
| Keratin, type I cytoskeletal 18 | P05783 | 96.59% | 59.22% | 52.17% | 79.83% | 74.40% | 63.43% |
| Keratin, type II cytoskeletal 1 | P04264 | | | | 3.51% | | |
| Keratin, type II cytoskeletal 2 epidermal | P35908 | | | | 20.05% | | |
| Keratin, type II cytoskeletal 7 | P08729 | | | | | 95.56% | |
| Keratin, type II cytoskeletal 8 | P05787 | 106.78% | 67.40% | 49.77% | 85.54% | 82.95% | 73.75% |
| Ketohexokinase | P50053 | | | -3.70% | | | |
| Lambda-crystallin homolog | Q9Y2S2 | | | 35.64% | | | |
| Leukotriene-B(4) omega-hydroxylase 1 | P78329 | | 78.03% | | | | |
| Liver carboxylesterase 1 | P23141 | 102.43% | 61.68% | 55.53% | 83.31% | 96.40% | 67.18% |
| L-lactate dehydrogenase A-like 6B | Q9BYZ2 | | 62.39% | 53.46% | 78.65% | | 46.24% |
| Long-chain-fatty-acid--CoA ligase 1 | P33121 | 69.44% | 68.04% | 66.40% | 74.73% | | 61.62% |
| Lumican | P51884 | | | | | 57.45% | 28.15% |
| L-xylulose reductase | Q7Z4W1 | 94.48% | 53.83% | 52.59% | 79.05% | 75.39% | 60.26% |
| Malate dehydrogenase, cytoplasmic | P40925 | | 55.30% | 43.80% | 58.63% | | 52.94% |
| Malate dehydrogenase, mitochondrial | P40926 | 84.10% | 44.03% | 46.84% | 69.83% | 65.16% | 42.33% |
| Maleylacetoacetate isomerase | O43708 | | 46.69% | 37.31% | | | 46.21% |
| Medium-chain specific acyl-CoA dehydrogenase, mitochondrial | P11310 | 96.44% | 62.43% | 77.63% | 88.80% | | 78.87% |
| Methylmalonate-semialdehyde dehydrogenase [acylating], mitochondrial | Q02252 | | 56.73% | 45.74% | 73.91% | | 59.50% |
| Microfibril-associated glycoprotein 4 | P55083 | | | | | 17.15% | |
| Microsomal glutathione S-transferase 1 | P10620 | 79.70% | 49.84% | 39.70% | 71.12% | 67.23% | 50.86% |
| Microsomal triglyceride transfer protein large subunit | P55157 | 99.42% | 72.17% | 63.65% | 94.45% | | 91.38% |
| Mimecan | P20774 | | | | | 16.20% | |
| Mitochondrial carrier homolog 2 | Q9Y6C9 | | 49.09% | 49.90% | 72.72% | | 52.62% |
| Moesin | P26038 | | | | | 93.49% | |
| Myoglobin | P02144 | | | | | | 17.05% |
| Myomesin-2 | P54296 | | | | | | 21.00% |

| Supplementary Table 2 | | Total fraction new at end of labeleing period - % new (F) | | | | | |
|---|---|---|---|---|---|---|---|
| | | **Subject #003** | **Subject #004** | **Subject #005** | **Subject #007** | **Subject #009** | **Subject #011** |
| | **Fibrosis Score** | **3** | **0** | **2** | **1** | **4** | **0** |
| Myosin light chain 1/3, skeletal muscle isoform | P05976 | | | | | | 16.24% |
| Myosin light chain 3 | P08590 | | | | | | 8.63% |
| Myosin light chain 6B | P14649 | | | | | 77.16% | 49.34% |
| Myosin light polypeptide 6 | P60660 | | 75.51% | 71.73% | 74.01% | 68.40% | 73.75% |
| Myosin regulatory light chain 2, skeletal muscle isoform | Q96A32 | | | | | | 21.25% |
| Myosin regulatory light chain 2, ventricular/cardiac muscle isoform | P10916 | | | | | | 18.24% |
| Myosin-1 | P12882 | | | | | | 19.94% |
| Myosin-10 | P35580 | | | | | 73.57% | |
| Myosin-11 | P35749 | | | | | 53.13% | |
| Myosin-13 | Q9UKX3 | | | | | | 13.61% |
| Myosin-2 | Q9UKX2 | | | | | | 25.48% |
| Myosin-6 | P13533 | | -23.23% | | 4.93% | | 19.11% |
| Myosin-7 | P12883 | | | | | | 20.75% |
| Myosin-9 | P35579 | 97.98% | 81.39% | 79.12% | 84.15% | 72.33% | 74.85% |
| Myosin-binding protein C, slow-type | Q00872 | | | | | | 43.17% |
| Myozenin-1 | Q9NP98 | | | | | | 28.36% |
| NAD kinase domain-containing protein 1 | Q4G0N4 | | | 8.44% | | | |
| NAD(P) transhydrogenase, mitochondrial | Q13423 | | 33.51% | 32.59% | | | |
| NADH dehydrogenase [ubiquinone] 1 alpha subcomplex subunit 13 | Q9P0J0 | | | | 75.07% | | |
| NADPH--cytochrome P450 reductase | P16435 | | 92.07% | | | | |
| Neutral alpha-glucosidase AB | Q14697 | | | 48.06% | | | |
| Nicotinate phosphoribosyltransferase | Q6XQN6 | | | 44.45% | | | |
| Nucleophosmin | P06748 | | 49.33% | 38.91% | | | |
| Nucleoside diphosphate kinase A | P15531 | | 60.73% | | | 74.18% | |
| Omega-amidase NIT2 | Q9NQR4 | | | | 72.23% | | |
| Ornithine carbamoyltransferase, mitochondrial | P00480 | | 54.02% | 38.08% | 83.87% | | 56.22% |

**Supplementary Table 2**

**Total fraction new at end of labeleing period - % new (F)**

| | **Fibrosis Score** | **Subject #003** | **Subject #004** | **Subject #005** | **Subject #007** | **Subject #009** | **Subject #011** |
|---|---|---|---|---|---|---|---|
| | | **3** | **0** | **2** | **1** | **4** | **0** |
| Peptidyl-prolyl cis-trans isomerase A | P62937 | | | 91.67% | | 88.78% | 80.71% |
| Peroxiredoxin-1 | Q06830 | 113.55% | 66.30% | 51.23% | | | 75.19% |
| Peroxiredoxin-2 | P32119 | 74.30% | 45.57% | 41.66% | 67.81% | | 50.46% |
| Peroxiredoxin-4 | Q13162 | 101.43% | 75.58% | 59.61% | 83.33% | | |
| Peroxiredoxin-5, mitochondrial | P30044 | | | 47.19% | | | |
| Peroxiredoxin-6 | P30041 | 96.97% | 62.59% | 49.23% | 71.85% | 75.63% | 55.24% |
| Peroxisomal acyl-coenzyme A oxidase 2 | Q99424 | | | | 20.56% | | |
| Peroxisomal bifunctional enzyme | Q08426 | 98.60% | 67.23% | 67.82% | 86.31% | 105.35% | 73.26% |
| Peroxisomal membrane protein 2 | Q9NR77 | | | | 92.93% | | |
| Peroxisomal multifunctional enzyme type 2 | P51659 | 101.43% | 70.11% | 70.14% | 90.40% | | 68.51% |
| Peroxisomal sarcosine oxidase | Q9P0Z9 | 64.13% | 53.52% | 58.83% | 85.88% | | 61.58% |
| Peroxisomal trans-2-enoyl-CoA reductase | Q9BY49 | | 42.79% | 50.02% | | | |
| Phenazine biosynthesis-like domain-containing protein | P30039 | | 38.00% | 40.40% | | | |
| Phosphate carrier protein, mitochondrial | Q00325 | 86.50% | 57.43% | 45.94% | 73.58% | 67.44% | 47.56% |
| Phosphatidylethanolamine-binding protein 1 | P30086 | 96.65% | 60.65% | 51.33% | 80.16% | 82.91% | 59.73% |
| Phosphoenolpyruvate carboxykinase [GTP], mitochondrial | Q16822 | 84.05% | 57.55% | 49.95% | 74.45% | 77.66% | 57.48% |
| Phosphoenolpyruvate carboxykinase, cytosolic [GTP] | P35558 | | 63.65% | 62.69% | 82.29% | | |
| Phosphoglucomutase-1 | P36871 | 96.80% | 54.30% | 43.81% | 79.97% | | 54.86% |
| Phosphoglycerate kinase 1 | P00558 | 97.36% | 58.09% | 36.56% | 68.44% | 64.62% | 40.24% |
| Phosphoglycerate mutase 1 | P18669 | 95.36% | 58.60% | 31.96% | 71.85% | 72.21% | 39.35% |
| Polypyrimidine tract-binding protein 1 | P26599 | | 58.03% | | | | |
| Prelamin-A/C | P02545 | 5.43% | 36.85% | 23.08% | 48.53% | 66.96% | 27.89% |
| Profilin-1 | P07737 | | 88.49% | 80.22% | 88.36% | 104.32% | 87.28% |
| Prohibitin | P35232 | 96.53% | 49.02% | 51.71% | 86.63% | 101.28% | 58.05% |
| Prohibitin-2 | Q99623 | 71.15% | 46.00% | 41.55% | 71.91% | 18.43% | 50.26% |
| Prolargin | P51888 | | | | | 38.97% | |

**Supplementary Table 2**

**Total fraction new at end of labeleing period - % new (F)**

| | | **Subject #003** | **Subject #004** | **Subject #005** | **Subject #007** | **Subject #009** | **Subject #011** |
|---|---|---|---|---|---|---|---|
| | **Fibrosis Score** | **3** | **0** | **2** | **1** | **4** | **0** |
| Proline dehydrogenase 1, mitochondrial | O43272 | | | | | 21.37% | |
| Prostaglandin reductase 1 | Q14914 | 84.92% | 56.61% | 46.36% | 66.97% | 78.71% | |
| Proteasome activator complex subunit 1 | Q06323 | | 74.50% | | | | |
| Proteasome activator complex subunit 2 | Q9UL46 | | 70.84% | 58.20% | | | 71.00% |
| Protein CutA | O60888 | | | 7.77% | | | |
| Protein disulfide-isomerase | P07237 | 110.56% | 71.56% | 64.98% | 86.54% | 100.77% | 76.72% |
| Protein disulfide-isomerase A3 | P30101 | | 71.91% | 87.76% | 84.34% | | 79.25% |
| Protein disulfide-isomerase A4 | P13667 | | 74.31% | 59.06% | | | 60.18% |
| Protein disulfide-isomerase A6 | Q15084 | 102.09% | 66.69% | 62.34% | 77.61% | 98.38% | 71.46% |
| Protein DJ-1 | Q99497 | | 61.06% | 57.94% | | | 55.25% |
| Protein NipSnap homolog 1 | Q9BPW8 | | 49.33% | 51.90% | 83.10% | | 62.89% |
| Protein NYNRIN | Q9P2P1 | 85.22% | | 41.49% | | | |
| Protein-glutamine gamma-glutamyltransferase 2 | P21980 | 101.47% | 73.31% | 56.47% | 88.70% | 79.74% | 68.45% |
| Pterin-4-alpha-carbinolamine dehydratase | P61457 | | | 49.03% | | | |
| Putative beta-actin-like protein 3 | Q9BYX7 | 76.53% | 44.32% | 40.68% | 60.75% | 45.26% | 28.44% |
| Putative inactive carboxylesterase 4 | Q9UKY3 | 106.50% | 68.23% | 62.41% | 87.05% | 97.52% | 69.07% |
| Pyruvate carboxylase, mitochondrial | P11498 | 84.02% | 52.72% | 48.42% | 72.71% | | 47.60% |
| Pyruvate kinase isozymes R/L | P30613 | | 66.91% | 60.74% | | | 67.13% |
| Quinone oxidoreductase | Q08257 | | 54.30% | 56.51% | | | 62.34% |
| Rab GDP dissociation inhibitor alpha | P31150 | 99.12% | | 57.55% | | | 66.03% |
| Rab GDP dissociation inhibitor beta | P50395 | | 74.85% | 65.71% | | | 76.39% |
| Ras GTPase-activating-like protein IQGAP2 | Q13576 | | | 59.52% | | | |
| Receptor expression-enhancing protein 6 | Q96HR9 | | 80.60% | 59.88% | 85.52% | | |
| Retinal dehydrogenase 1 | P00352 | 97.40% | 59.75% | 55.09% | 84.09% | 84.25% | 61.17% |
| Retinol dehydrogenase 16 | O75452 | | 63.16% | 47.07% | | | |
| Ribonuclease UK114 | P52758 | 90.90% | 57.47% | 47.12% | 77.07% | 72.55% | 58.10% |
| Ribose-5-phosphate isomerase | P49247 | | | 8.50% | | | |

**Supplementary Table 2** — **Total fraction new at end of labeleing period - % new (F)**

| | Fibrosis Score | Subject #003<br>3 | Subject #004<br>0 | Subject #005<br>2 | Subject #007<br>1 | Subject #009<br>4 | Subject #011<br>0 |
|---|---|---|---|---|---|---|---|
| Sarcoplasmic/endoplasmic reticulum calcium ATPase 1 | O14983 | | | | | | 32.75% |
| Sarcoplasmic/endoplasmic reticulum calcium ATPase 2 | P16615 | | | | | | 44.23% |
| Sarcosine dehydrogenase, mitochondrial | Q9UL12 | | | 49.87% | | 55.01% | |
| Selenium-binding protein 1 | Q13228 | 100.13% | 61.51% | 49.31% | 71.53% | 68.26% | 57.80% |
| Serine hydroxymethyltransferase, cytosolic | P34896 | | 65.24% | 54.48% | | | 66.30% |
| Serine--pyruvate aminotransferase | P21549 | 97.20% | 70.56% | 64.00% | 81.66% | 88.47% | 70.00% |
| Serum albumin | P02768 | 74.70% | 42.28% | 40.74% | 64.77% | 34.71% | 43.65% |
| Short/branched chain specific acyl-CoA dehydrogenase, mitochondrial | P45954 | | 65.41% | 66.79% | 87.24% | | 72.42% |
| Short-chain specific acyl-CoA dehydrogenase, mitochondrial | P16219 | | 50.51% | 40.06% | 71.11% | | 46.61% |
| Sideroflexin-1 | Q9H9B4 | | 46.26% | 38.92% | | | |
| Sorbitol dehydrogenase | Q00796 | | 60.02% | 44.98% | 81.07% | 87.07% | 63.64% |
| Spectrin alpha chain, brain | Q13813 | | 70.25% | 65.02% | 90.36% | | 64.46% |
| Stress-70 protein, mitochondrial | P38646 | 111.66% | 81.66% | 80.74% | 82.17% | | 87.68% |
| Succinate dehydrogenase [ubiquinone] flavoprotein subunit, mitochondrial | P31040 | 75.76% | 51.06% | 52.81% | 81.00% | 86.16% | 58.93% |
| Succinate-semialdehyde dehydrogenase, mitochondrial | P51649 | | 38.68% | 40.82% | | | 38.61% |
| Sulfotransferase 1A1 | P50225 | | | 50.45% | 77.33% | 69.59% | 62.08% |
| Superoxide dismutase [Cu-Zn] | P00441 | | | 77.04% | | | |
| Superoxide dismutase [Mn], mitochondrial | P04179 | 99.13% | 48.74% | 50.26% | 76.52% | 84.95% | 61.84% |
| Talin-1 | Q9Y490 | | | 6.22% | | | |
| Thioredoxin-dependent peroxide reductase, mitochondrial | P30048 | 94.12% | 59.17% | 63.42% | 94.07% | 73.70% | 60.16% |
| Thiosulfate sulfurtransferase | Q16762 | 95.70% | 61.43% | 45.45% | 83.71% | | 65.91% |
| Thymidine phosphorylase | P19971 | 103.48% | 64.23% | | 81.03% | 66.82% | 66.87% |

**Supplementary Table 2** — **Total fraction new at end of labeleing period - % new (F)**

| | Fibrosis Score | Subject #003<br>3 | Subject #004<br>0 | Subject #005<br>2 | Subject #007<br>1 | Subject #009<br>4 | Subject #011<br>0 |
|---|---|---|---|---|---|---|---|
| Titin | Q8WZ42 | | | | | | 28.12% |
| Transforming growth factor-beta-induced protein ig-h3 | Q15582 | | | | | 103.62% | |
| Transgelin | Q01995 | | | | | 62.94% | |
| Transgelin-2 | P37802 | | | | | 71.02% | |
| Transketolase | P29401 | | 48.87% | | | 47.36% | 39.44% |
| Tricarboxylate transport protein, mitochondrial | P53007 | | | | | | 55.27% |
| Trifunctional enzyme subunit alpha, mitochondrial | P40939 | 84.65% | 44.90% | 37.30% | 74.26% | 66.14% | 46.94% |
| Trifunctional enzyme subunit beta, mitochondrial | P55084 | | 47.70% | 42.83% | 78.34% | | 51.25% |
| Triosephosphate isomerase | P60174 | 86.29% | 50.76% | 41.19% | 70.87% | 58.56% | 45.80% |
| Tripeptidyl-peptidase 1 | O14773 | | 54.05% | 30.78% | 69.24% | 72.00% | |
| Tropomyosin alpha-1 chain | P09493 | | | | 75.11% | 50.45% | 23.08% |
| Tropomyosin alpha-3 chain | P06753 | | | | | | 29.78% |
| Tropomyosin alpha-4 chain | P67936 | | | | | 54.15% | |
| Tropomyosin beta chain | P07951 | | | | | | 20.82% |
| Troponin C, slow skeletal and cardiac muscles | P63316 | | | | | | 28.16% |
| Tubulin alpha-1A chain | Q71U36 | 88.66% | 56.89% | 43.86% | 75.24% | 66.14% | 58.59% |
| Tubulin alpha-1B chain | P68363 | 75.74% | 46.92% | 40.63% | 72.03% | 58.64% | 38.13% |
| Tubulin alpha-4A chain | P68366 | 108.81% | 58.55% | | 95.15% | | 59.21% |
| Tubulin beta chain | P07437 | | 66.06% | 68.24% | | | |
| Tubulin beta-2A chain | Q13885 | 98.48% | 73.68% | 36.99% | 64.56% | 50.43% | 46.89% |
| Tubulin beta-3 chain | Q13509 | | | | | | 51.47% |
| Tubulin beta-4A chain | P04350 | | 65.53% | | | | |
| Ubiquitin-60S ribosomal protein L40 | P62987 | 124.84% | 93.91% | 97.14% | 94.16% | 111.51% | 93.48% |
| UDP-glucose 6-dehydrogenase | O60701 | | 67.65% | 54.40% | | | |
| UDP-glucuronosyltransferase 1-1 | P22309 | | 64.75% | | | | |
| UDP-glucuronosyltransferase 1-10 | Q9HAW8 | | 52.52% | | | | 40.34% |

**Supplementary Table 2**

**Total fraction new at end of labeleing period - % new (F)**

| | **Fibrosis Score** | **Subject #003** 3 | **Subject #004** 0 | **Subject #005** 2 | **Subject #007** 1 | **Subject #009** 4 | **Subject #011** 0 |
|---|---|---|---|---|---|---|---|
| UDP-glucuronosyltransferase 1-4 | P22310 | | 79.04% | | | | |
| UDP-glucuronosyltransferase 2A1 | Q9Y4X1 | | | 60.70% | 73.50% | | 56.13% |
| UDP-glucuronosyltransferase 2B15 | P54855 | | | 89.81% | | | |
| UDP-glucuronosyltransferase 2B4 | P06133 | | | 44.70% | | | |
| UDP-glucuronosyltransferase 2B7 | P16662 | 111.36% | 68.99% | 66.76% | | | 95.86% |
| Urocanate hydratase | Q96N76 | | | 53.39% | | | |
| UTP--glucose-1-phosphate uridylyltransferase | Q16851 | 103.20% | 61.15% | 59.13% | 86.37% | 102.06% | 66.56% |
| Very long-chain acyl-CoA synthetase | O14975 | 114.20% | 74.30% | 68.38% | 89.92% | | 77.31% |
| Very long-chain specific acyl-CoA dehydrogenase, mitochondrial | P49748 | | | 72.56% | | | 67.33% |
| Vimentin | P08670 | 101.01% | 63.80% | 52.31% | 93.73% | 56.15% | 57.56% |
| Vinculin | P18206 | | 35.15% | | | 35.94% | 33.64% |
| Voltage-dependent anion-selective channel protein 1 | P21796 | | 49.24% | | | | |

**Supplementary Table 2**

| Liver Proteins | Uniprot Accession | Subject #003 | Subject #004 | Subject #005 | Subject #007 | Subject #009 | Subject #011 | Protein FSR vs Fibrosis Score ($r^2$) |
|---|---|---|---|---|---|---|---|---|
| | | Fractional synthesis rate - % new per day (k) | | | | | | |
| | Fibrosis Score | 3 | 0 | 2 | 1 | 4 | 0 | |
| 1,4-alpha-glucan-branching enzyme | Q04446 | | | 1.85% | | | | |
| 14-3-3 protein epsilon | P62258 | | | | | | 6.13% | |
| 14-3-3 protein eta | Q04917 | | | | | 1.92% | 2.19% | |
| 17-beta-hydroxysteroid dehydrogenase 13 | Q7Z5P4 | | 6.47% | 7.85% | ≥4.11% | | 6.44% | |
| 17-beta-hydroxysteroid dehydrogenase type 6 | O14756 | | | 4.93% | 2.42% | | 6.39% | |
| 2,4-dienoyl-CoA reductase, mitochondrial | Q16698 | ≥6.98% | 3.27% | 3.61% | 3.37% | | 4.90% | |
| 3-hydroxyacyl-CoA dehydratase 3 | Q9P035 | | | 4.25% | | | | |
| 3-hydroxyacyl-CoA dehydrogenase type-2 | Q99714 | 2.26% | 2.52% | 2.77% | 1.32% | | 3.05% | |
| 3-hydroxyanthranilate 3,4-dioxygenase | P46952 | | | 2.62% | | | | |
| 3-ketoacyl-CoA thiolase, mitochondrial | P42765 | 5.11% | 2.88% | 3.00% | 2.72% | 7.10% | 3.68% | 0.69 |
| 3-ketoacyl-CoA thiolase, peroxisomal | P09110 | ≥6.98% | 6.57% | 5.55% | 3.35% | ≥10.47% | 5.48% | |
| 3-mercaptopyruvate sulfurtransferase | P25325 | ≥6.98% | 3.89% | 3.90% | 2.74% | | 4.17% | |
| 40S ribosomal protein S7 | P62081 | | 5.55% | 4.93% | 4.03% | | 5.50% | |
| 40S ribosomal protein S8 | P62241 | 0.80% | 0.27% | 0.28% | 0.82% | 1.72% | 0.44% | 0.60 |
| 40S ribosomal protein SA | P08865 | 6.06% | 3.56% | 3.69% | 2.42% | 5.65% | 3.71% | 0.60 |
| 4-aminobutyrate aminotransferase, mitochondrial | P80404 | ≥6.98% | 3.98% | 3.28% | 2.61% | ≥10.47% | 4.29% | |
| 4-hydroxyphenylpyruvate dioxygenase | P32754 | | ≥10.47% | 7.56% | | | 7.01% | |
| 4-trimethylaminobutyraldehyde dehydrogenase | P49189 | | 2.81% | 2.99% | | | | |
| 60 kDa heat shock protein, mitochondrial | P10809 | 6.19% | 3.19% | 3.02% | 2.37% | | 3.69% | |
| 60S acidic ribosomal protein P0 | P05388 | | | 3.68% | | | | |
| 60S ribosomal protein L6 | Q02878 | 4.76% | 3.27% | 3.64% | 2.03% | 3.65% | 3.62% | 0.20 |
| 60S ribosomal protein L7 | P18124 | ≥6.98% | | | | | | |
| 78 kDa glucose-regulated protein | P11021 | ≥6.98% | 6.55% | 4.91% | 3.67% | ≥10.47% | 7.51% | |
| Abhydrolase domain-containing protein 14B | Q96IU4 | | 6.87% | | | | | |

| Supplementary Table 2 | | Fractional synthesis rate - % new per day (k) | | | | | | |
|---|---|---|---|---|---|---|---|---|
| | | Subject #003 | Subject #004 | Subject #005 | Subject #007 | Subject #009 | Subject #011 | Protein FSR vs Fibrosis Score ($r^2$) |
| | Fibrosis Score | 3 | 0 | 2 | 1 | 4 | 0 | |
| Acetyl-CoA acetyltransferase, cytosolic | Q9BWD1 | | 3.33% | 3.74% | 2.00% | | 7.85% | |
| Acetyl-CoA acetyltransferase, mitochondrial | P24752 | 5.50% | 3.41% | 2.67% | 2.76% | 5.55% | 3.30% | 0.57 |
| Aconitate hydratase, mitochondrial | Q99798 | | 5.51% | | | | | |
| Actin, alpha skeletal muscle | P68133 | | | | | | 0.47% | |
| Actin, aortic smooth muscle | P62736 | ≥6.98% | 3.22% | 3.36% | 2.29% | 2.15% | 0.65% | |
| Actin, cytoplasmic 1 | P60709 | 5.72% | 3.73% | 3.43% | 2.41% | 3.32% | 3.78% | 0.07 |
| Acyl-coenzyme A synthetase ACSM2A, mitochondrial | Q08AH3 | | 3.67% | 3.80% | | | | |
| Acyl-coenzyme A thioesterase 1 | Q86TX2 | | 4.22% | | | | 4.06% | |
| Adenylate kinase 2, mitochondrial | P54819 | | 2.75% | 3.52% | | | | |
| ADP/ATP translocase 1 | P12235 | | 3.59% | 5.84% | 3.46% | | 3.75% | |
| Aflatoxin B1 aldehyde reductase member 3 | O95154 | | 6.59% | | | | | |
| Agmatinase, mitochondrial | Q9BSE5 | | | 2.05% | | | | |
| Alanine aminotransferase 1 | P24298 | | | 1.66% | | | | |
| Alcohol dehydrogenase [NADP+] | P14550 | | | 5.71% | | | | |
| Alcohol dehydrogenase 1A | P07327 | ≥6.98% | 4.16% | 2.88% | 2.73% | 5.53% | 3.98% | |
| Alcohol dehydrogenase 1B | P00325 | ≥6.98% | 4.00% | 2.58% | 2.42% | 8.45% | 4.10% | |
| Alcohol dehydrogenase 1C | P00326 | ≥6.98% | 4.31% | | 3.29% | | 4.30% | |
| Alcohol dehydrogenase 4 | P08319 | ≥6.98% | 4.34% | 3.15% | 2.57% | 9.10% | 4.55% | |
| Alcohol dehydrogenase 6 | P28332 | | 2.87% | 3.34% | 2.15% | | 3.84% | |
| Alcohol dehydrogenase class-3 | P11766 | | 2.88% | 1.95% | | | | |
| Aldehyde dehydrogenase family 8 member A1 | Q9H2A2 | | 5.49% | 4.08% | | | 4.46% | |
| Aldehyde dehydrogenase X, mitochondrial | P30837 | ≥6.98% | 4.30% | | ≥4.11% | ≥10.47% | 4.54% | |
| Aldehyde dehydrogenase, mitochondrial | P05091 | ≥6.98% | 5.23% | 4.69% | 3.24% | ≥10.47% | 5.63% | |
| Aldehyde oxidase | Q06278 | | 5.56% | 6.37% | 3.28% | | 5.03% | |
| Aldo-keto reductase family 1 member C1 | Q04828 | | 4.52% | 3.14% | 2.08% | 9.84% | 3.86% | |
| Aldo-keto reductase family 1 member C3 | P42330 | | | 3.24% | | | | |
| Aldo-keto reductase family 1 member C4 | P17516 | | | 3.38% | | | | |
| Alpha-1-antitrypsin | P01009 | | 6.38% | | | ≥10.47% | 9.53% | |

**Supplementary Table 2**

| | | Fractional synthesis rate - % new per day (k) | | | | | | |
|---|---|---|---|---|---|---|---|---|
| | | Subject #003 | Subject #004 | Subject #005 | Subject #007 | Subject #009 | Subject #011 | Protein FSR vs Fibrosis Score ($r^2$) |
| | Fibrosis Score | 3 | 0 | 2 | 1 | 4 | 0 | |
| Alpha-2-macroglobulin | P01023 | 5.78% | 4.49% | 3.97% | 2.25% | 6.10% | 3.44% | 0.52 |
| Alpha-actinin-1 | P12814 | ≥6.98% | 6.06% | | 3.37% | 5.19% | 2.34% | |
| Alpha-actinin-2 | P35609 | | | | | | 1.20% | |
| Alpha-actinin-4 | O43707 | 5.43% | 4.02% | 4.28% | | 4.05% | 3.18% | |
| Alpha-enolase | P06733 | ≥6.98% | 4.82% | 2.75% | 3.22% | ≥10.47% | 5.82% | |
| Amine oxidase [flavin-containing] A | P21397 | | 2.76% | 2.96% | 2.09% | | | |
| Amine oxidase [flavin-containing] B | P27338 | ≥6.98% | 2.97% | 1.57% | 3.15% | | | |
| Aminoacylase-1 | Q03154 | | 4.05% | 3.52% | 2.27% | ≥10.47% | 5.22% | |
| Annexin A1 | P04083 | | | | | 5.36% | | |
| Annexin A2 | P07355 | | 3.94% | | | 3.69% | 3.26% | |
| Annexin A4 | P09525 | | | | | 10.33% | | |
| Annexin A5 | P08758 | | 4.80% | 5.12% | 3.11% | 6.73% | 4.40% | |
| Annexin A6 | P08133 | ≥6.98% | 6.62% | 6.40% | ≥4.11% | 9.85% | 5.87% | |
| Apolipoprotein A-I | P02647 | | | | | 6.12% | | |
| Apoptosis-inducing factor 1, mitochondrial | O95831 | | 2.49% | 1.61% | 1.92% | | 2.38% | |
| Arginase-1 | P05089 | 6.41% | 3.62% | 2.91% | 2.28% | | 4.06% | |
| Argininosuccinate lyase | P04424 | 5.48% | 3.83% | 4.27% | 1.88% | 6.43% | 4.06% | 0.54 |
| Argininosuccinate synthase | P00966 | | 4.96% | 3.49% | 2.52% | | 6.28% | |
| Arylacetamide deacetylase | P22760 | | | | | | 7.09% | |
| Aspartate aminotransferase, cytoplasmic | P17174 | ≥6.98% | 4.53% | 3.83% | 3.26% | | 4.08% | |
| Aspartate aminotransferase, mitochondrial | P00505 | 5.09% | 3.15% | 2.87% | 2.38% | 5.73% | 2.95% | 0.70 |
| ATP synthase subunit alpha, mitochondrial | P25705 | ≥6.98% | 3.86% | 3.73% | 3.45% | 6.42% | 4.03% | |
| ATP synthase subunit b, mitochondrial | P24539 | | 2.73% | | 2.21% | | | |
| ATP synthase subunit beta, mitochondrial | P06576 | ≥6.98% | 3.05% | 2.68% | 3.02% | 7.42% | 3.23% | |
| ATP synthase subunit delta, mitochondrial | P30049 | | | | 3.00% | | | |
| ATP synthase subunit f, mitochondrial | P56134 | | 2.56% | | | | | |
| ATP synthase subunit O, mitochondrial | P48047 | 6.14% | 2.50% | 6.27% | 1.57% | | 4.36% | |
| ATP-binding cassette sub-family D member 3 | P28288 | | 4.74% | 4.28% | 3.01% | | 6.93% | |
| Beta-actin-like protein 2 | Q562R1 | | | 2.54% | | | | |

| Supplementary Table 2 | | Fractional synthesis rate - % new per day (k) | | | | | | |
|---|---|---|---|---|---|---|---|---|
| | | Subject #003 | Subject #004 | Subject #005 | Subject #007 | Subject #009 | Subject #011 | Protein FSR vs Fibrosis Score ($r^2$) |
| | Fibrosis Score | 3 | 0 | 2 | 1 | 4 | 0 | |
| Beta-enolase | P13929 | | | | | | 3.59% | |
| Betaine--homocysteine S-methyltransferase 1 | Q93088 | ≥6.98% | 4.70% | 3.98% | 2.93% | | 4.94% | |
| Betaine--homocysteine S-methyltransferase 2 | Q9H2M3 | | | 4.49% | | | 3.40% | |
| Bifunctional ATP-dependent dihydroxyacetone kinase/FAD-AMP lyase (cyclizing) | Q3LXA3 | ≥6.98% | 3.84% | 3.93% | 2.25% | | 4.05% | |
| Biglycan | P21810 | | | | | 1.80% | | |
| Bile acid-CoA:amino acid N-acyltransferase | Q14032 | | 4.08% | 2.67% | | | 6.38% | |
| Bile acyl-CoA synthetase | Q9Y2P5 | | 6.26% | 4.28% | 3.42% | | 5.82% | |
| Bile salt sulfotransferase | Q06520 | ≥6.98% | 4.43% | 3.96% | 3.19% | | 4.94% | |
| C-1-tetrahydrofolate synthase, cytoplasmic | P11586 | 3.90% | 3.30% | 2.67% | 1.58% | | 4.21% | |
| Calcium-binding mitochondrial carrier protein Aralar1 | O75746 | | 2.87% | | 1.78% | | 3.33% | |
| Calcium-binding mitochondrial carrier protein Aralar2 | Q9UJS0 | 6.12% | 2.80% | 2.13% | 2.51% | | 3.60% | |
| Caldesmon | Q05682 | | | | | 7.57% | | |
| Calmodulin | P62158 | | | | | ≥10.47% | 10.61% | |
| Calnexin | P27824 | ≥6.98% | 6.22% | 5.14% | | | 5.78% | |
| Calreticulin | P27797 | ≥6.98% | 5.01% | 4.77% | 2.54% | 8.55% | 6.04% | |
| Calsequestrin-1 | P31415 | | | | | | 0.79% | |
| Carbamoyl-phosphate synthase [ammonia], mitochondrial | P31327 | 6.33% | 2.95% | 2.71% | 2.63% | 7.33% | 3.40% | 0.70 |
| Carbonic anhydrase 1 | P00915 | | | 1.54% | | 4.01% | | |
| Carbonic anhydrase 2 | P00918 | 6.76% | 3.91% | 3.06% | 2.54% | 6.09% | 3.81% | 0.49 |
| Carbonic anhydrase 3 | P07451 | | | | 3.70% | | 1.20% | |
| Carbonyl reductase [NADPH] 1 | P16152 | ≥6.98% | 4.06% | 3.73% | 2.28% | 6.68% | 4.20% | |
| Catalase | P04040 | ≥6.98% | 4.95% | 5.37% | 3.21% | 7.67% | 5.76% | |
| Cathepsin D | P07339 | ≥6.98% | 5.22% | 4.35% | 3.63% | ≥10.47% | 6.59% | |
| Clathrin heavy chain 1 | Q00610 | | | | | | 5.03% | |
| Cocaine esterase | O00748 | | 2.94% | 2.08% | | | | |

**Supplementary Table 2**

| | | Fractional synthesis rate - % new per day (k) | | | | | | |
|---|---|---|---|---|---|---|---|---|
| | | Subject #003 | Subject #004 | Subject #005 | Subject #007 | Subject #009 | Subject #011 | Protein FSR vs Fibrosis Score ($r^2$) |
| | Fibrosis Score | 3 | 0 | 2 | 1 | 4 | 0 | |
| Cofilin-1 | P23528 | | | 1.68% | | 7.14% | | |
| Collagen alpha-1(I) chain | P02452 | 0.63% | 0.20% | 0.39% | 0.23% | 0.56% | 0.32% | 0.79 |
| Collagen alpha-1(III) chain | P02461 | 0.59% | 0.25% | 0.24% | 0.18% | 0.42% | 0.33% | 0.38 |
| Collagen alpha-1(IV) chain | P02462 | | | 0.09% | | 0.93% | | |
| Collagen alpha-1(VI) chain | P12109 | 1.30% | 0.82% | 0.95% | 0.75% | 1.14% | 0.88% | 0.66 |
| Collagen alpha-1(XIV) chain | Q05707 | | | | | 2.19% | | |
| Collagen alpha-2(I) chain | P08123 | 0.66% | 0.41% | 0.49% | 0.24% | 0.56% | 0.42% | 0.48 |
| Collagen alpha-3(VI) chain | P12111 | 1.00% | 1.28% | 0.66% | 0.52% | 0.94% | 1.02% | 0.04 |
| Complement C3 | P01024 | | ≥10.47% | | 3.04% | | | |
| Complement C4-A | P0C0L4 | | 7.83% | | | | | |
| Corticosteroid 11-beta-dehydrogenase isozyme 1 | P28845 | | 7.09% | | | | | |
| Creatine kinase M-type | P06732 | | | | | | 0.85% | |
| Creatine kinase S-type, mitochondrial | P17540 | | | | | | 2.43% | |
| Cullin-associated NEDD8-dissociated protein 1 | Q86VP6 | | 3.20% | 3.22% | | | | |
| Cystathionine gamma-lyase | P32929 | ≥6.98% | 6.38% | 4.10% | ≥4.11% | | 5.28% | |
| Cytochrome b5 | P00167 | ≥6.98% | 4.83% | 4.23% | 3.37% | 10.23% | 5.40% | |
| Cytochrome b-c1 complex subunit 2, mitochondrial | P22695 | | | | | | 4.04% | |
| Cytochrome c oxidase subunit 2 | P00403 | 5.82% | 3.90% | 2.84% | ≥4.11% | 7.70% | 3.82% | |
| Cytochrome c oxidase subunit 5A, mitochondrial | P20674 | | 2.50% | 2.15% | 1.88% | | 2.59% | |
| Cytochrome P450 1A2 | P05177 | | 6.02% | | 1.88% | | | |
| Cytochrome P450 2A6 | P11509 | | ≥10.47% | | | | | |
| Cytochrome P450 2C8 | P10632 | | ≥10.47% | ≥11.51% | | | | |
| Cytochrome P450 2C9 | P11712 | ≥6.98% | 8.33% | 4.98% | 3.51% | | 7.87% | |
| Cytochrome P450 2E1 | P05181 | | | | 3.46% | | | |
| Cytochrome P450 3A4 | P08684 | | | 7.08% | | | | |
| Cytochrome P450 3A5 | P20815 | | 6.66% | | 2.53% | ≥10.47% | 7.08% | |

**Supplementary Table 2**

| | | Fractional synthesis rate - % new per day (k) | | | | | | |
|---|---|---|---|---|---|---|---|---|
| | | **Subject #003** | **Subject #004** | **Subject #005** | **Subject #007** | **Subject #009** | **Subject #011** | **Protein FSR vs Fibrosis Score ($r^2$)** |
| | **Fibrosis Score** | **3** | **0** | **2** | **1** | **4** | **0** | |
| Cytoplasmic aconitate hydratase | P21399 | | 4.29% | 3.76% | 2.97% | | | |
| Cytosol aminopeptidase | P28838 | | 5.07% | | | | 6.42% | |
| Cytosolic 10-formyltetrahydrofolate dehydrogenase | O75891 | | 3.48% | 4.70% | ≥4.11% | | 4.13% | |
| Cytosolic non-specific dipeptidase | Q96KP4 | 3.75% | 3.85% | 1.86% | | | 7.86% | |
| D-beta-hydroxybutyrate dehydrogenase, mitochondrial | Q02338 | ≥6.98% | 3.01% | 4.26% | | | 3.52% | |
| D-dopachrome decarboxylase | P30046 | | 4.21% | 3.26% | 3.02% | 7.61% | 4.45% | |
| Delta(3,5)-Delta(2,4)-dienoyl-CoA isomerase, mitochondrial | Q13011 | 6.46% | 3.14% | | 2.48% | 6.05% | 4.04% | |
| Delta-1-pyrroline-5-carboxylate dehydrogenase, mitochondrial | P30038 | ≥6.98% | 3.66% | 3.55% | 3.23% | 3.11% | 4.29% | |
| Delta-aminolevulinic acid dehydratase | P13716 | | | 2.33% | | | | |
| Desmin | P17661 | | | | | 4.36% | 4.60% | |
| Dihydropyrimidinase | Q14117 | | | 4.85% | ≥4.11% | | 5.75% | |
| Dimethylaniline monooxygenase [N-oxide-forming] 3 | P31513 | | 5.42% | 4.00% | 2.70% | | | |
| Dimethylglycine dehydrogenase, mitochondrial | Q9UI17 | | 3.06% | 4.94% | | | | |
| Dolichyl-diphosphooligosaccharide--protein glycosyltransferase subunit 1 | P04843 | 6.82% | 3.23% | | 2.16% | | | |
| Dolichyl-diphosphooligosaccharide--protein glycosyltransferase subunit 2 | P04844 | | 4.90% | 3.96% | 2.51% | | 3.89% | |
| Elastin | P15502 | | | | | 0.37% | | |
| Electron transfer flavoprotein subunit alpha, mitochondrial | P13804 | 6.93% | 3.11% | 3.08% | 2.54% | 6.87% | 3.27% | 0.71 |
| Electron transfer flavoprotein subunit beta | P38117 | 3.37% | 3.01% | 2.79% | 3.41% | | 4.30% | |
| Electron transfer flavoprotein-ubiquinone oxidoreductase, mitochondrial | Q16134 | | 5.26% | 4.53% | | | 6.95% | |

| Supplementary Table 2 | | Fractional synthesis rate - % new per day (k) | | | | | | |
|---|---|---|---|---|---|---|---|---|
| | | Subject #003 | Subject #004 | Subject #005 | Subject #007 | Subject #009 | Subject #011 | Protein FSR vs Fibrosis Score ($r^2$) |
| | Fibrosis Score | 3 | 0 | 2 | 1 | 4 | 0 | |
| Elongation factor 1-alpha 1 | P68104 | ≥6.98% | 4.39% | 4.09% | 2.09% | 6.76% | 3.81% | |
| Elongation factor 2 | P13639 | | 8.68% | 6.94% | | | | |
| Endoplasmin | P14625 | ≥6.98% | 6.01% | 5.32% | 3.78% | ≥10.47% | 7.14% | |
| Enoyl-CoA hydratase domain-containing protein 2, mitochondrial | Q86YB7 | | 3.47% | 4.35% | ≥4.11% | | 7.76% | |
| Enoyl-CoA hydratase, mitochondrial | P30084 | 6.06% | 2.71% | 2.71% | 2.29% | 5.88% | 3.20% | 0.67 |
| Epoxide hydrolase 1 | P07099 | ≥6.98% | 4.61% | 3.75% | 3.11% | 9.09% | 5.18% | |
| Epoxide hydrolase 2 | P34913 | | 5.10% | 3.73% | 2.94% | | 6.35% | |
| Ester hydrolase C11orf54 | Q9H0W9 | | 3.61% | 5.69% | | | | |
| Estradiol 17-beta-dehydrogenase 11 | Q8NBQ5 | | | 4.96% | | | | |
| Fatty acid synthase | P49327 | ≥6.98% | 9.13% | ≥11.51% | 2.82% | | 8.19% | |
| Fatty acid-binding protein, liver | P07148 | ≥6.98% | 6.04% | 3.23% | 2.74% | 8.22% | 4.26% | |
| Ferritin light chain | P02792 | ≥6.98% | 5.53% | 7.45% | 3.25% | ≥10.47% | 5.02% | |
| Fibronectin | P02751 | ≥6.98% | 4.26% | | | 7.76% | 7.77% | |
| Filamin-A | P21333 | | | | ≥4.11% | 3.51% | 7.70% | |
| Filamin-B | O75369 | | | 4.67% | | | | |
| Filamin-C | Q14315 | | | | | | 1.46% | |
| Flavin reductase (NADPH) | P30043 | | 3.48% | 3.94% | 2.14% | | 5.03% | |
| Formimidoyltransferase-cyclodeaminase | O95954 | ≥6.98% | 4.42% | 3.93% | 2.94% | 9.32% | 4.36% | |
| Fructose-1,6-bisphosphatase 1 | P09467 | ≥6.98% | 5.22% | 3.69% | 3.62% | 7.44% | 5.01% | |
| Fructose-bisphosphate aldolase A | P04075 | | | | | | 2.60% | |
| Fructose-bisphosphate aldolase B | P05062 | ≥6.98% | 6.71% | 5.38% | 3.31% | ≥10.47% | 6.47% | |
| Fumarate hydratase, mitochondrial | P07954 | | 3.20% | 3.03% | 2.60% | | 3.40% | |
| Fumarylacetoacetase | P16930 | 6.95% | 3.79% | 2.80% | 2.22% | 5.44% | 3.31% | 0.43 |
| Galactokinase | P51570 | | 3.17% | 2.73% | | | 3.41% | |
| Galectin-1 | P09382 | | | | | 4.05% | 4.50% | |
| Gamma-glutamyltransferase 5 | P36269 | ≥6.98% | | 4.37% | | 4.26% | | |
| Gelsolin | P06396 | | 3.93% | 5.21% | | | | |
| Glucose-6-phosphate isomerase | P06744 | 4.49% | 3.35% | 3.02% | 2.54% | 5.84% | 3.35% | 0.62 |

**Supplementary Table 2**

| | | Fractional synthesis rate - % new per day (k) | | | | | | |
|---|---|---|---|---|---|---|---|---|
| | | **Subject #003** | **Subject #004** | **Subject #005** | **Subject #007** | **Subject #009** | **Subject #011** | **Protein FSR vs Fibrosis Score ($r^2$)** |
| | **Fibrosis Score** | **3** | **0** | **2** | **1** | **4** | **0** | |
| Glutamate dehydrogenase 1, mitochondrial | P00367 | 6.24% | 2.62% | 2.42% | 2.62% | | 2.71% | |
| Glutathione S-transferase A1 | P08263 | ≥6.98% | 3.99% | 2.68% | 2.45% | 9.25% | 4.08% | |
| Glutathione S-transferase kappa 1 | Q9Y2Q3 | | 5.17% | | | | 6.51% | |
| Glutathione S-transferase Mu 1 | P09488 | ≥6.98% | | 3.71% | 1.93% | | 5.01% | |
| Glutathione S-transferase omega-1 | P78417 | | | 2.21% | | | | |
| Glyceraldehyde-3-phosphate dehydrogenase | P04406 | ≥6.98% | 4.12% | 3.49% | 2.80% | 4.67% | 3.33% | |
| Glycerol-3-phosphate dehydrogenase [NAD+], cytoplasmic | P21695 | | 4.19% | 3.11% | | | | |
| Glycine amidinotransferase, mitochondrial | P50440 | ≥6.98% | 5.47% | 5.72% | 3.03% | ≥10.47% | 5.26% | |
| Glycine dehydrogenase [decarboxylating], mitochondrial | P23378 | | | 0.87% | | | | |
| Glycogen debranching enzyme | P35573 | | | 1.11% | | | 2.11% | |
| Glycogen phosphorylase, brain form | P11216 | | | | | | 2.26% | |
| Glycogen phosphorylase, liver form | P06737 | 4.28% | 2.89% | 1.80% | 1.66% | | 1.85% | |
| Glycogen phosphorylase, muscle form | P11217 | | | | | | 1.48% | |
| Glyoxylate reductase/hydroxypyruvate reductase | Q9UBQ7 | 6.29% | 3.83% | 3.10% | 2.39% | 6.48% | 3.83% | 0.55 |
| Heat shock 70 kDa protein 1A/1B | P08107 | | 6.01% | 5.90% | | 5.89% | 4.30% | |
| Heat shock cognate 71 kDa protein | P11142 | | 7.48% | | 4.10% | | | |
| Heat shock protein beta-1 | P04792 | ≥6.98% | 5.36% | 5.05% | 2.93% | 6.61% | 4.20% | |
| Heat shock-related 70 kDa protein 2 | P54652 | | | 4.50% | 3.82% | | 8.35% | |
| Hemoglobin subunit alpha | P69905 | 1.10% | 0.49% | 0.65% | 0.83% | 2.36% | 0.60% | 0.72 |
| Hemoglobin subunit beta | P68871 | 1.04% | 0.46% | 0.34% | 0.89% | 2.09% | 0.74% | 0.55 |
| Hemoglobin subunit delta | P02042 | 1.52% | 1.03% | 1.49% | 1.40% | 3.24% | 1.14% | 0.70 |
| Hemoglobin subunit gamma-1 | P69891 | | | 0.85% | | | | |
| Heterogeneous nuclear ribonucleoprotein K | P61978 | ≥6.98% | 6.79% | 11.01% | | ≥10.47% | | |
| Histone H2A type 1-A | Q96QV6 | | 2.01% | 1.40% | 1.31% | 1.64% | 1.61% | |
| Histone H2A type 1-B/E | P04908 | 2.40% | 1.42% | 0.55% | 0.97% | 1.27% | 1.09% | 0.09 |
| Histone H2B type 1-A | Q96A08 | | | | | 1.36% | | |

**Supplementary Table 2**

| | | Fractional synthesis rate - % new per day (k) | | | | | | |
|---|---|---|---|---|---|---|---|---|
| | | Subject #003 | Subject #004 | Subject #005 | Subject #007 | Subject #009 | Subject #011 | Protein FSR vs Fibrosis Score ($r^2$) |
| | Fibrosis Score | 3 | 0 | 2 | 1 | 4 | 0 | |
| Histone H2B type 1-B | P33778 | 2.42% | 1.44% | 0.98% | 1.15% | 1.08% | 1.27% | 0.03 |
| Histone H3.1t | Q16695 | 1.07% | 0.95% | 0.33% | 0.45% | 0.57% | 0.50% | 0.00 |
| Histone H3.3 | P84243 | 1.20% | 0.61% | 0.81% | 0.42% | 0.54% | 0.74% | 0.07 |
| Histone H4 | P62805 | 1.78% | 0.97% | 0.75% | 0.75% | 1.12% | 1.00% | 0.22 |
| HLA class I histocompatibility antigen, A-1 alpha chain | P30443 | ≥6.98% | | | | | | |
| HLA class I histocompatibility antigen, A-25 alpha chain | P18462 | ≥6.98% | | | | | | |
| Homogentisate 1,2-dioxygenase | Q93099 | | | 2.54% | ≥4.11% | | 6.67% | |
| Hydroxyacid oxidase 1 | Q9UJM8 | ≥6.98% | 6.28% | 7.10% | ≥4.11% | | 6.26% | |
| Hydroxyacyl-coenzyme A dehydrogenase, mitochondrial | Q16836 | 6.72% | 2.60% | 2.56% | 2.29% | 4.46% | 2.71% | 0.48 |
| Hydroxymethylglutaryl-CoA synthase, cytoplasmic | Q01581 | | | 4.94% | ≥4.11% | | 6.29% | |
| Hydroxymethylglutaryl-CoA synthase, mitochondrial | P54868 | ≥6.98% | 4.84% | 4.96% | 3.30% | ≥10.47% | 4.99% | |
| Ig alpha-1 chain C region | P01876 | | | | | 5.99% | | |
| Ig gamma-1 chain C region | P01857 | 3.37% | 3.91% | 1.00% | 1.78% | 3.00% | 3.05% | 0.01 |
| Ig gamma-2 chain C region | P01859 | | | | | 2.46% | | |
| Ig heavy chain V-III region BRO | P01766 | | | | | 4.10% | | |
| Ig kappa chain C region | P01834 | 3.02% | 3.36% | 1.63% | 1.20% | 2.96% | 1.97% | 0.06 |
| Ig lambda-2 chain C regions | P0CG05 | | | | | 3.91% | | |
| Immunoglobulin lambda-like polypeptide 5 | B9A064 | | 3.81% | | | 4.38% | | |
| Isochorismatase domain-containing protein 2, mitochondrial | Q96AB3 | 4.56% | 2.37% | 2.22% | 1.05% | | 3.05% | |
| Isocitrate dehydrogenase [NADP] cytoplasmic | O75874 | ≥6.98% | 3.75% | 3.62% | 2.90% | | 3.49% | |
| Isocitrate dehydrogenase [NADP], mitochondrial | P48735 | 5.02% | 1.97% | 2.85% | | 4.28% | 2.76% | |
| Keratin, type I cytoskeletal 10 | P13645 | | | | 0.23% | | | |

| Supplementary Table 2 | | Fractional synthesis rate - % new per day (k) | | | | | | |
|---|---|---|---|---|---|---|---|---|
| | | Subject #003 | Subject #004 | Subject #005 | Subject #007 | Subject #009 | Subject #011 | Protein FSR vs Fibrosis Score ($r^2$) |
| | Fibrosis Score | 3 | 0 | 2 | 1 | 4 | 0 | |
| Keratin, type I cytoskeletal 18 | P05783 | ≥6.98% | 4.08% | 3.69% | 2.86% | 6.19% | 4.79% | |
| Keratin, type II cytoskeletal 1 | P04264 | | | | 0.06% | | | |
| Keratin, type II cytoskeletal 2 epidermal | P35908 | | | | 0.40% | | | |
| Keratin, type II cytoskeletal 7 | P08729 | | | | | ≥10.47% | | |
| Keratin, type II cytoskeletal 8 | P05787 | ≥6.98% | 5.10% | 3.44% | 3.45% | 8.04% | 6.37% | |
| Ketohexokinase | P50053 | | | -0.18% | | | | |
| Lambda-crystallin homolog | Q9Y2S2 | | | 2.20% | | | | |
| Leukotriene-B(4) omega-hydroxylase 1 | P78329 | | 6.89% | | | | | |
| Liver carboxylesterase 1 | P23141 | ≥6.98% | 4.36% | 4.05% | 3.20% | ≥10.47% | 5.31% | |
| L-lactate dehydrogenase A-like 6B | Q9BYZ2 | | 4.45% | 3.82% | 2.76% | | 2.96% | |
| Long-chain-fatty-acid--CoA ligase 1 | P33121 | 3.59% | 5.19% | 5.45% | 2.46% | | 4.56% | |
| Lumican | P51884 | | | | | 3.88% | 1.57% | |
| L-xylulose reductase | Q7Z4W1 | ≥6.98% | 3.51% | 3.73% | 2.79% | 6.37% | 4.39% | |
| Malate dehydrogenase, cytoplasmic | P40925 | | 3.66% | 2.88% | 1.58% | | 3.59% | |
| Malate dehydrogenase, mitochondrial | P40926 | 5.57% | 2.64% | 3.16% | 2.14% | 4.79% | 2.62% | 0.71 |
| Maleylacetoacetate isomerase | O43708 | | 2.86% | 2.34% | | | 2.95% | |
| Medium-chain specific acyl-CoA dehydrogenase, mitochondrial | P11310 | ≥6.98% | 4.45% | 7.49% | 3.91% | | 7.40% | |
| Methylmalonate-semialdehyde dehydrogenase [acylating], mitochondrial | Q02252 | | 3.81% | 3.06% | 2.40% | | 4.30% | |
| Microfibril-associated glycoprotein 4 | P55083 | | | | | 0.86% | | |
| Microsomal glutathione S-transferase 1 | P10620 | 4.83% | 3.14% | 2.53% | 2.22% | 5.07% | 3.38% | 0.49 |
| Microsomal triglyceride transfer protein large subunit | P55157 | ≥6.98% | 5.81% | 5.06% | ≥4.11% | | ≥10.96% | |
| Mimecan | P20774 | | | | | 0.80% | | |
| Mitochondrial carrier homolog 2 | Q9Y6C9 | | 3.07% | 3.46% | 2.32% | | 3.56% | |
| Moesin | P26038 | | | | | ≥10.47% | | |
| Myoglobin | P02144 | | | | | | 0.89% | |
| Myomesin-2 | P54296 | | | | | | 1.12% | |

| Supplementary Table 2 | | Fractional synthesis rate - % new per day (k) | | | | | | |
|---|---|---|---|---|---|---|---|---|
| | | Subject #003 | Subject #004 | Subject #005 | Subject #007 | Subject #009 | Subject #011 | Protein FSR vs Fibrosis Score ($r^2$) |
| | Fibrosis Score | 3 | 0 | 2 | 1 | 4 | 0 | |
| Myosin light chain 1/3, skeletal muscle isoform | P05976 | | | | | | 0.84% | |
| Myosin light chain 3 | P08590 | | | | | | 0.43% | |
| Myosin light chain 6B | P14649 | | | | | 6.71% | 3.24% | |
| Myosin light polypeptide 6 | P60660 | | 6.40% | 6.32% | 2.41% | 5.24% | 6.37% | |
| Myosin regulatory light chain 2, skeletal muscle isoform | Q96A32 | | | | | | 1.14% | |
| Myosin regulatory light chain 2, ventricular/cardiac muscle isoform | P10916 | | | | | | 0.96% | |
| Myosin-1 | P12882 | | | | | | 1.06% | |
| Myosin-10 | P35580 | | | | | 6.05% | | |
| Myosin-11 | P35749 | | | | | 3.44% | | |
| Myosin-13 | Q9UKX3 | | | | | | 0.70% | |
| Myosin-2 | Q9UKX2 | | | | | | 1.40% | |
| Myosin-6 | P13533 | | -0.95% | | 0.09% | | 1.01% | |
| Myosin-7 | P12883 | | | | | | 1.11% | |
| Myosin-9 | P35579 | ≥6.98% | 7.64% | 7.83% | 3.29% | 5.84% | 6.57% | |
| Myosin-binding protein C, slow-type | Q00872 | | | | | | 2.69% | |
| Myozenin-1 | Q9NP98 | | | | | | 1.59% | |
| NAD kinase domain-containing protein 1 | Q4G0N4 | | | 0.44% | | | | |
| NAD(P) transhydrogenase, mitochondrial | Q13423 | | 1.86% | 1.97% | | | | |
| NADH dehydrogenase [ubiquinone] 1 alpha subcomplex subunit 13 | Q9P0J0 | | | | 2.48% | | | |
| NADPH--cytochrome P450 reductase | P16435 | | ≥10.47% | | | | | |
| Neutral alpha-glucosidase AB | Q14697 | | | 3.28% | | | | |
| Nicotinate phosphoribosyltransferase | Q6XQN6 | | | 2.94% | | | | |
| Nucleophosmin | P06748 | | 3.09% | 2.46% | | | | |
| Nucleoside diphosphate kinase A | P15531 | | 4.25% | | | 6.15% | | |
| Omega-amidase NIT2 | Q9NQR4 | | | | 2.29% | | | |
| Ornithine carbamoyltransferase, mitochondrial | P00480 | | 3.53% | 2.40% | 3.26% | | 3.93% | |

**Supplementary Table 2**

| | | Fractional synthesis rate - % new per day (k) | | | | | | |
|---|---|---|---|---|---|---|---|---|
| | | Subject #003 | Subject #004 | Subject #005 | Subject #007 | Subject #009 | Subject #011 | Protein FSR vs Fibrosis Score ($r^2$) |
| | Fibrosis Score | 3 | 0 | 2 | 1 | 4 | 0 | |
| Peptidyl-prolyl cis-trans isomerase A | P62937 | | | ≥11.51% | | 9.94% | 7.84% | |
| Peroxiredoxin-1 | Q06830 | ≥6.98% | 4.94% | 3.59% | | | 6.64% | |
| Peroxiredoxin-2 | P32119 | 4.12% | 2.77% | 2.69% | 2.02% | | 3.34% | |
| Peroxiredoxin-4 | Q13162 | ≥6.98% | 6.41% | 4.53% | 3.20% | | | |
| Peroxiredoxin-5, mitochondrial | P30044 | | | 3.19% | | | | |
| Peroxiredoxin-6 | P30041 | ≥6.98% | 4.47% | 3.39% | 2.26% | 6.42% | 3.83% | |
| Peroxisomal acyl-coenzyme A oxidase 2 | Q99424 | | | | 0.41% | | | |
| Peroxisomal bifunctional enzyme | Q08426 | ≥6.98% | 5.07% | 5.67% | 3.55% | ≥10.47% | 6.28% | |
| Peroxisomal membrane protein 2 | Q9NR77 | | | | ≥4.11% | | | |
| Peroxisomal multifunctional enzyme type 2 | P51659 | ≥6.98% | 5.49% | 6.04% | ≥4.11% | | 5.50% | |
| Peroxisomal sarcosine oxidase | Q9P0Z9 | 3.11% | 3.48% | 4.44% | 3.50% | | 4.55% | |
| Peroxisomal trans-2-enoyl-CoA reductase | Q9BY49 | | 2.54% | 3.47% | | | | |
| Phenazine biosynthesis-like domain-containing protein | P30039 | | 2.17% | 2.59% | | | | |
| Phosphate carrier protein, mitochondrial | Q00325 | 6.07% | 3.88% | 3.08% | 2.38% | 5.10% | 3.07% | 0.47 |
| Phosphatidylethanolamine-binding protein 1 | P30086 | ≥6.98% | 4.24% | 3.60% | 2.89% | 8.03% | 4.33% | |
| Phosphoenolpyruvate carboxykinase [GTP], mitochondrial | Q16822 | 5.56% | 3.89% | 3.46% | 2.44% | 6.81% | 4.07% | 0.56 |
| Phosphoenolpyruvate carboxykinase, cytosolic [GTP] | P35558 | | 4.60% | 4.93% | 3.09% | | | |
| Phosphoglucomutase-1 | P36871 | ≥6.98% | 3.56% | 2.88% | 2.87% | | 3.79% | |
| Phosphoglycerate kinase 1 | P00558 | ≥6.98% | 3.95% | 2.28% | 2.06% | 4.72% | 2.45% | |
| Phosphoglycerate mutase 1 | P18669 | ≥6.98% | 4.01% | 1.93% | 2.26% | 5.82% | 2.38% | |
| Polypyrimidine tract-binding protein 1 | P26599 | | 3.95% | | | | | |
| Prelamin-A/C | P02545 | 0.17% | 2.09% | 1.31% | 1.19% | 5.03% | 1.56% | 0.17 |
| Profilin-1 | P07737 | | 9.83% | 8.10% | 3.84% | ≥10.47% | 9.82% | |
| Prohibitin | P35232 | ≥6.98% | 3.06% | 3.64% | 3.59% | ≥10.47% | 4.14% | |
| Prohibitin-2 | Q99623 | 3.77% | 2.80% | 2.69% | 2.27% | 0.93% | 3.33% | 0.21 |
| Prolargin | P51888 | | | | | 2.24% | | |

| Supplementary Table 2 | | Fractional synthesis rate - % new per day (k) | | | | | | |
|---|---|---|---|---|---|---|---|---|
| | | Subject #003 | Subject #004 | Subject #005 | Subject #007 | Subject #009 | Subject #011 | Protein FSR vs Fibrosis Score ($r^2$) |
| | Fibrosis Score | 3 | 0 | 2 | 1 | 4 | 0 | |
| Proline dehydrogenase 1, mitochondrial | O43272 | | | | | 1.09% | | |
| Prostaglandin reductase 1 | Q14914 | 5.73% | 3.80% | 3.11% | 1.98% | 7.03% | | |
| Proteasome activator complex subunit 1 | Q06323 | | 6.21% | | | | | |
| Proteasome activator complex subunit 2 | Q9UL46 | | 5.60% | 4.36% | | | 5.90% | |
| Protein CutA | O60888 | | | 0.40% | | | | |
| Protein disulfide-isomerase | P07237 | ≥6.98% | 5.72% | 5.25% | 3.58% | ≥10.47% | 6.94% | |
| Protein disulfide-isomerase A3 | P30101 | | 5.77% | 10.50% | 3.31% | | 7.49% | |
| Protein disulfide-isomerase A4 | P13667 | | 6.18% | 4.47% | | | 4.38% | |
| Protein disulfide-isomerase A6 | Q15084 | ≥6.98% | 5.00% | 4.88% | 2.67% | ≥10.47% | 5.97% | |
| Protein DJ-1 | Q99497 | | 4.29% | 4.33% | | | 3.83% | |
| Protein NipSnap homolog 1 | Q9BPW8 | | 3.09% | 3.66% | 3.17% | | 4.72% | |
| Protein NYNRIN | Q9P2P1 | 5.79% | | 2.68% | | | | |
| Protein-glutamine gamma-glutamyltransferase 2 | P21980 | ≥6.98% | 6.00% | 4.16% | 3.89% | 7.26% | 5.49% | |
| Pterin-4-alpha-carbinolamine dehydratase | P61457 | | | 3.37% | | | | |
| Putative beta-actin-like protein 3 | Q9BYX7 | 4.39% | 2.66% | 2.61% | 1.67% | 2.74% | 1.59% | 0.35 |
| Putative inactive carboxylesterase 4 | Q9UKY3 | ≥6.98% | 5.21% | 4.89% | 3.65% | ≥10.47% | 5.59% | |
| Pyruvate carboxylase, mitochondrial | P11498 | 5.56% | 3.41% | 3.31% | 2.32% | | 3.08% | |
| Pyruvate kinase isozymes R/L | P30613 | | 5.03% | 4.67% | | | 5.30% | |
| Quinone oxidoreductase | Q08257 | | 3.56% | 4.16% | | | 4.65% | |
| Rab GDP dissociation inhibitor alpha | P31150 | ≥6.98% | | 4.28% | | | 5.14% | |
| Rab GDP dissociation inhibitor beta | P50395 | | 6.27% | 5.35% | | | 6.87% | |
| Ras GTPase-activating-like protein IQGAP2 | Q13576 | | | 4.52% | | | | |
| Receptor expression-enhancing protein 6 | Q96HR9 | | 7.45% | 4.57% | 3.45% | | | |
| Retinal dehydrogenase 1 | P00352 | ≥6.98% | 4.14% | 4.00% | 3.28% | 8.40% | 4.51% | |
| Retinol dehydrogenase 16 | O75452 | | 4.54% | 3.18% | | | | |
| Ribonuclease UK114 | P52758 | ≥6.98% | 3.89% | 3.19% | 2.63% | 5.88% | 4.14% | |
| Ribose-5-phosphate isomerase | P49247 | | | 0.44% | | | | |

| Supplementary Table 2 | | Fractional synthesis rate - % new per day (k) | | | | | | |
|---|---|---|---|---|---|---|---|---|
| | | Subject #003 | Subject #004 | Subject #005 | Subject #007 | Subject #009 | Subject #011 | Protein FSR vs Fibrosis Score ($r^2$) |
| | Fibrosis Score | 3 | 0 | 2 | 1 | 4 | 0 | |
| Sarcoplasmic/endoplasmic reticulum calcium ATPase 1 | O14983 | | | | | | 1.89% | |
| Sarcoplasmic/endoplasmic reticulum calcium ATPase 2 | P16615 | | | | | | 2.78% | |
| Sarcosine dehydrogenase, mitochondrial | Q9UL12 | | | 3.45% | | 3.63% | | |
| Selenium-binding protein 1 | Q13228 | ≥6.98% | 4.34% | 3.40% | 2.24% | 5.22% | 4.11% | |
| Serine hydroxymethyltransferase, cytosolic | P34896 | | 4.80% | 3.94% | | | 5.18% | |
| Serine--pyruvate aminotransferase | P21549 | ≥6.98% | 5.56% | 5.11% | 3.03% | 9.82% | 5.73% | |
| Serum albumin | P02768 | 4.16% | 2.50% | 2.62% | 1.86% | 1.94% | 2.73% | 0.02 |
| Short/branched chain specific acyl-CoA dehydrogenase, mitochondrial | P45954 | | 4.83% | 5.51% | 3.68% | | 6.13% | |
| Short-chain specific acyl-CoA dehydrogenase, mitochondrial | P16219 | | 3.20% | 2.56% | 2.22% | | 2.99% | |
| Sideroflexin-1 | Q9H9B4 | | 2.82% | 2.46% | | | | |
| Sorbitol dehydrogenase | Q00796 | | 4.17% | 2.99% | 2.97% | 9.30% | 4.82% | |
| Spectrin alpha chain, brain | Q13813 | | 5.51% | 5.25% | ≥4.11% | | 4.93% | |
| Stress-70 protein, mitochondrial | P38646 | ≥6.98% | 7.71% | 8.24% | 3.08% | | 9.97% | |
| Succinate dehydrogenase [ubiquinone] flavoprotein subunit, mitochondrial | P31040 | 4.29% | 3.25% | 3.76% | 2.97% | 8.99% | 4.24% | 0.55 |
| Succinate-semialdehyde dehydrogenase, mitochondrial | P51649 | | 2.22% | 2.62% | | | 2.32% | |
| Sulfotransferase 1A1 | P50225 | | | 3.51% | 2.65% | 5.41% | 4.62% | |
| Superoxide dismutase [Cu-Zn] | P00441 | | | 7.36% | | | | |
| Superoxide dismutase [Mn], mitochondrial | P04179 | ≥6.98% | 3.04% | 3.49% | 2.59% | 8.61% | 4.59% | |
| Talin-1 | Q9Y490 | | | 0.32% | | | | |
| Thioredoxin-dependent peroxide reductase, mitochondrial | P30048 | ≥6.98% | 4.07% | 5.03% | ≥4.11% | 6.07% | 4.38% | |
| Thiosulfate sulfurtransferase | Q16762 | ≥6.98% | 4.33% | 3.03% | 3.24% | | 5.12% | |
| Thymidine phosphorylase | P19971 | ≥6.98% | 4.67% | | 2.97% | 5.01% | 5.26% | |

| Supplementary Table 2 | | Fractional synthesis rate - % new per day (k) | | | | | | |
|---|---|---|---|---|---|---|---|---|
| | | Subject #003 | Subject #004 | Subject #005 | Subject #007 | Subject #009 | Subject #011 | Protein FSR vs Fibrosis Score ($r^2$) |
| | Fibrosis Score | 3 | 0 | 2 | 1 | 4 | 0 | |
| Titin | Q8WZ42 | | | | | | 1.57% | |
| Transforming growth factor-beta-induced protein ig-h3 | Q15582 | | | | | ≥10.47% | | |
| Transgelin | Q01995 | | | | | 4.51% | | |
| Transgelin-2 | P37802 | | | | | 5.63% | | |
| Transketolase | P29401 | | 3.05% | | | 2.92% | 2.39% | |
| Tricarboxylate transport protein, mitochondrial | P53007 | | | | | | 3.83% | |
| Trifunctional enzyme subunit alpha, mitochondrial | P40939 | 5.68% | 2.71% | 2.33% | 2.42% | 4.92% | 3.02% | 0.56 |
| Trifunctional enzyme subunit beta, mitochondrial | P55084 | | 2.95% | 2.80% | 2.73% | | 3.42% | |
| Triosephosphate isomerase | P60174 | 6.02% | 3.22% | 2.65% | 2.20% | 4.00% | 2.92% | 0.34 |
| Tripeptidyl-peptidase 1 | O14773 | | 3.53% | 1.84% | 2.11% | 5.79% | | |
| Tropomyosin alpha-1 chain | P09493 | | | | 2.48% | 3.19% | 1.25% | |
| Tropomyosin alpha-3 chain | P06753 | | | | | | 1.68% | |
| Tropomyosin alpha-4 chain | P67936 | | | | | 3.54% | | |
| Tropomyosin beta chain | P07951 | | | | | | 1.11% | |
| Troponin C, slow skeletal and cardiac muscles | P63316 | | | | | | 1.57% | |
| Tubulin alpha-1A chain | Q71U36 | 6.60% | 3.83% | 2.89% | 2.49% | 4.92% | 4.20% | 0.26 |
| Tubulin alpha-1B chain | P68363 | 4.29% | 2.88% | 2.61% | 2.28% | 4.01% | 2.29% | 0.66 |
| Tubulin alpha-4A chain | P68366 | ≥6.98% | 4.00% | | ≥4.11% | | 4.27% | |
| Tubulin beta chain | P07437 | | 4.91% | 5.73% | | | | |
| Tubulin beta-2A chain | Q13885 | ≥6.98% | 6.07% | 2.31% | 1.85% | 3.19% | 3.01% | |
| Tubulin beta-3 chain | Q13509 | | | | | | 3.44% | |
| Tubulin beta-4A chain | P04350 | | 4.84% | | | | | |
| Ubiquitin-60S ribosomal protein L40 | P62987 | ≥6.98% | ≥10.47% | ≥11.51% | ≥4.11% | ≥10.47% | ≥10.96% | |
| UDP-glucose 6-dehydrogenase | O60701 | | 5.13% | 3.93% | | | | |
| UDP-glucuronosyltransferase 1-1 | P22309 | | 4.74% | | | | | |
| UDP-glucuronosyltransferase 1-10 | Q9HAW8 | | 3.39% | | | | 2.46% | |

**Supplementary Table 2**

| | Fibrosis Score | Subject #003 | Subject #004 | Subject #005 | Subject #007 | Subject #009 | Subject #011 | Protein FSR vs Fibrosis Score ($r^2$) |
|---|---|---|---|---|---|---|---|---|
| | | Fractional synthesis rate - % new per day (k) | | | | | | |
| | | 3 | 0 | 2 | 1 | 4 | 0 | |
| UDP-glucuronosyltransferase 1-4 | P22310 | | 7.10% | | | | | |
| UDP-glucuronosyltransferase 2A1 | Q9Y4X1 | | | 4.67% | 2.37% | | 3.92% | |
| UDP-glucuronosyltransferase 2B15 | P54855 | | | 11.42% | | | | |
| UDP-glucuronosyltransferase 2B4 | P06133 | | | 2.96% | | | | |
| UDP-glucuronosyltransferase 2B7 | P16662 | ≥6.98% | 5.32% | 5.51% | | | ≥10.96% | |
| Urocanate hydratase | Q96N76 | | | 3.82% | | | | |
| UTP--glucose-1-phosphate uridylyltransferase | Q16851 | ≥6.98% | 4.30% | 4.47% | 3.56% | ≥10.47% | 5.22% | |
| Very long-chain acyl-CoA synthetase | O14975 | ≥6.98% | 6.18% | 5.76% | 4.10% | | 7.06% | |
| Very long-chain specific acyl-CoA dehydrogenase, mitochondrial | P49748 | | | 6.47% | | | 5.33% | |
| Vimentin | P08670 | ≥6.98% | 4.62% | 3.70% | ≥4.11% | 3.75% | 4.08% | |
| Vinculin | P18206 | | 1.97% | | | 2.02% | 1.95% | |
| Voltage-dependent anion-selective channel protein 1 | P21796 | | 3.08% | | | | | |
