# Supplementary material for: Turnover Rates of Hepatic Collagen and Circulating Collagen-Associated Proteins in Humans with Chronic Liver Disease
Source: PLoS One. 2015 Apr 24;10(4):e0123311. doi: 10.1371/journal.pone.0123311 (PMC4409311; doi:10.1371/journal.pone.0123311)
Supplement: S3 Table — (PDF) [file pone.0123311.s004.pdf]

Supplementary Table 3 **Total fraction new at end of labeleing period - % new (F)**

Plasma Proteins	Uniprot Accession	Subject #003	Subject #004	Subject #005	Subject #007	Subject #008	Subject #009	Subject #010	Subject #011
Actin, aortic smooth muscle	P62736							91.02%	
Afamin	P43652	102.66%	75.37%	71.85%	87.82%	100.64%	79.62%	90.51%	72.39%
Alpha-1-antichymotrypsin	P01011	107.96%	80.75%	77.32%	92.35%	102.84%	80.31%	94.27%	76.24%
Alpha-1B-glycoprotein	P04217	99.86%	80.83%	77.31%	88.39%	96.55%	80.66%	91.59%	77.76%
Alpha-2-antiplasmin	P08697	107.22%	87.68%	90.33%	93.52%	100.12%	86.59%	95.97%	85.21%
Alpha-2-HS-glycoprotein	P02765	104.24%	74.60%	75.08%	87.44%	103.38%	79.05%	90.05%	87.29%
Alpha-2-macroglobulin	P01023	91.26%	46.79%	45.45%	80.47%	90.72%	65.99%	61.44%	41.06%
Angiotensinogen	P01019	115.19%	92.78%	95.51%	100.91%	108.68%	85.99%	103.38%	97.05%
Antithrombin-III	P01008	103.57%	83.97%	84.96%	92.26%	100.47%	86.51%	95.83%	87.67%
Apolipoprotein A-II	P02652	114.48%	78.72%	85.94%		102.82%		97.03%	80.62%
Apolipoprotein A-IV	P06727	84.09%	79.08%	71.64%	69.36%	80.02%	85.90%	76.51%	80.14%
Apolipoprotein B-100	P04114	104.04%	86.13%	85.66%	91.09%	100.43%	85.49%	95.46%	87.63%
Apolipoprotein E	P02649	94.66%	93.38%	90.06%	49.88%	99.08%	94.20%	94.96%	91.51%
Apolipoprotein(a)	P08519		68.42%					80.21%	
Beta-2-glycoprotein 1	P02749	96.32%	80.17%	84.18%	85.11%	98.22%	74.03%	87.00%	84.38%
Beta-2-microglobulin	P61769						72.17%		
Biotinidase	P43251			73.91%		99.98%	80.31%	78.93%	73.18%
Carbonic anhydrase 1	P00915								12.13%
Carboxypeptidase B2	Q96IY4		72.38%			88.93%			
Carboxypeptidase N catalytic chain	P15169		84.67%	82.20%		111.55%		92.37%	72.93%
Carboxypeptidase N subunit 2	P22792		92.99%					101.20%	91.67%
Ceruloplasmin	P00450	102.34%	66.21%	62.85%	85.28%	96.93%	68.49%	82.24%	62.06%
Cholinesterase	P06276					92.43%			49.46%
Clusterin	P10909	105.31%	94.90%	91.64%	96.04%	104.94%	95.31%	104.31%	99.68%
Coagulation factor X	P00742		91.77%	81.33%		101.94%		100.57%	89.17%
Coagulation factor XII	P00748	106.45%	76.07%	78.02%	88.29%	95.88%	78.60%	90.71%	72.33%
Complement C1q subcomponent subunit A	P02745	92.76%	69.27%	70.21%		90.62%	81.71%		
Complement C1q subcomponent subunit B	P02746				72.76%	87.42%	82.14%		
Complement C1q subcomponent subunit C	P02747	93.91%	67.65%	62.73%	74.43%	82.17%	83.37%	79.52%	59.54%

Supplementary Table 3

Total fraction new at end of labeleing period - % new (F)

Plasma Proteins	Uniprot Accession	Subject #003	Subject #004	Subject #005	Subject #007	Subject #008	Subject #009	Subject #010	Subject #011
Complement C1r subcomponent	P00736	96.64%	84.38%	88.82%	86.00%	94.20%	90.72%	86.04%	90.98%
Complement C1r subcomponent-like protein	Q9NZP8	87.89%	74.07%	74.11%	79.46%	85.66%	77.34%	80.81%	76.04%
Complement C1s subcomponent	P09871	96.90%	85.28%	92.71%	90.28%	95.24%	93.76%	88.14%	93.26%
Complement C2	P06681	109.74%	88.00%	89.18%	92.48%	100.98%	97.09%	96.77%	88.85%
Complement C3	P01024	106.01%	86.32%				84.33%	93.05%	
Complement C4-A	P0C0L4	104.53%	87.71%	87.39%	92.30%	100.24%	85.19%	96.84%	85.44%
Complement C4-B	P0C0L5	108.88%	86.90%	92.72%		104.58%	71.52%		
Complement C5	P01031	100.81%	81.46%	78.18%	88.73%	101.19%	80.16%	92.94%	80.90%
Complement component C6	P13671	115.37%	91.73%	36.13%	84.43%	105.90%	89.04%	98.33%	74.40%
Complement component C7	P10643	94.35%	74.23%	68.12%	83.34%	97.06%	87.18%	83.36%	67.38%
Complement component C8 alpha chain	P07357	109.28%	82.98%	83.06%	93.93%	101.37%	84.60%	99.73%	79.35%
Complement component C8 beta chain	P07358	96.66%	75.09%	83.43%	79.45%	91.96%	71.80%	89.54%	80.08%
Complement component C8 gamma chain	P07360	105.66%	79.08%	78.23%	89.75%	100.38%	83.65%	88.68%	80.64%
Complement component C9	P02748	111.25%	84.14%	88.01%	89.81%	102.75%	87.07%	93.26%	83.24%
Complement factor B	P00751	107.47%	83.66%	90.87%	92.00%	104.43%	90.69%	95.87%	84.65%
Complement factor H	P08603	103.72%	73.17%	71.04%	87.43%	102.10%	76.49%	87.38%	70.74%
Complement factor I	P05156	112.87%	92.69%	92.74%	99.98%	108.84%	95.75%	106.20%	91.77%
Corticosteroid-binding globulin	P08185	104.29%	72.67%			99.83%	75.61%	87.59%	70.13%
Extracellular matrix protein 1	Q16610				79.77%	87.13%	91.23%	95.35%	
Fibrinogen alpha chain	P02671	100.43%	65.27%	75.72%	89.71%	103.11%	74.66%	88.73%	72.32%
Fibrinogen beta chain	P02675	88.95%		64.53%	86.93%	99.22%	66.09%	78.99%	62.43%
Fibrinogen gamma chain	P02679			67.64%	88.61%	101.21%	73.79%	80.61%	63.63%
Fibronectin	P02751	97.76%	84.82%	79.59%	87.95%	96.94%	79.11%	89.97%	83.65%
Ficolin-3	O75636	99.53%	72.57%	89.48%		97.06%	68.65%	79.95%	70.06%
Gelsolin	P06396	98.52%	79.71%	79.20%	84.57%	100.18%	90.00%	92.19%	80.52%
Glutathione peroxidase 3	P22352			52.92%			69.13%		
Haptoglobin	P00738			82.09%					
Haptoglobin-related protein	P00739		91.03%	83.29%					
Hemoglobin subunit alpha	P69905	35.62%					39.18%		7.85%

Supplementary Table 3 — **Total fraction new at end of labeleing period - % new (F)**

Plasma Proteins	Uniprot Accession	Subject #003	Subject #004	Subject #005	Subject #007	Subject #008	Subject #009	Subject #010	Subject #011
Hemoglobin subunit beta	P68871	32.43%	6.96%	12.26%			34.57%		7.57%
Hemoglobin subunit delta	P02042						23.36%		
Hemoglobin subunit gamma-1	P69891								15.80%
Hemopexin	P02790	91.29%	61.51%	49.69%	80.18%	92.98%	39.45%	70.05%	56.93%
Heparin cofactor 2	P05546		69.70%			70.65%		76.17%	55.27%
Histidine-rich glycoprotein	P04196	115.80%	93.59%	87.13%	95.66%	110.78%	95.18%	106.10%	79.05%
Ig gamma-3 chain C region	P01860						55.98%		
Ig kappa chain C region	P01834	45.46%					64.64%		
Ig mu chain C region	P01871						52.60%		
Immunoglobulin lambda-like polypeptide 5	B9A064						55.33%		
Insulin-like growth factor-binding protein complex acid labile subunit	P35858	89.92%	74.89%	80.70%	89.78%		72.58%	84.88%	
Inter-alpha-trypsin inhibitor heavy chain H1	P19827	106.37%	83.79%	83.60%	89.05%	100.82%	72.24%	95.93%	81.25%
Inter-alpha-trypsin inhibitor heavy chain H2	P19823	101.97%	82.22%	84.66%	86.38%	102.36%	76.62%	91.77%	83.21%
Inter-alpha-trypsin inhibitor heavy chain H3	Q06033	104.84%	90.66%	90.46%	91.44%	97.76%	87.76%	100.97%	88.31%
Inter-alpha-trypsin inhibitor heavy chain H4	Q14624	101.81%	82.31%	83.64%	86.66%	96.66%	81.14%	90.28%	79.84%
Kallistatin	P29622	94.22%	81.85%	78.50%	83.23%	89.35%	80.27%	66.39%	80.04%
Kininogen-1	P01042	107.19%	83.50%	77.84%	91.31%	102.50%	85.88%	93.86%	78.15%
Leucine-rich alpha-2-glycoprotein	P02750	107.22%	80.63%	79.80%	92.24%	101.62%	82.25%	94.50%	81.56%
Low affinity immunoglobulin gamma Fc region receptor III-A	P08637						67.90%		
Lumican	P51884	57.56%	28.45%	29.49%	49.46%	49.16%	47.95%	40.90%	20.04%
N-acetylmuramoyl-L-alanine amidase	Q96PD5	104.68%	77.93%	82.17%	83.98%	97.11%	76.64%	88.41%	84.62%
Peptidase inhibitor 16	Q6UXB8			91.72%	80.48%				

Supplementary Table 3 — **Total fraction new at end of labeleing period - % new (F)**

Plasma Proteins	Uniprot Accession	Subject #003	Subject #004	Subject #005	Subject #007	Subject #008	Subject #009	Subject #010	Subject #011
Phosphatidylinositol-glycan-specific phospholipase D	P80108								95.87%
Pigment epithelium-derived factor	P36955	107.15%	88.45%	92.40%	93.32%	103.10%	101.11%	100.25%	91.71%
Plasma kallikrein	P03952	110.88%	70.22%	74.98%	97.04%	101.99%	72.29%	88.05%	69.30%
Plasma protease C1 inhibitor	P05155	102.86%	82.25%	85.93%	85.87%	108.62%	90.26%	98.21%	92.39%
Plasminogen	P00747	94.49%	80.28%	79.34%	83.89%	92.68%	78.50%	86.08%	78.91%
Plasminogen-related protein B	Q02325								55.41%
Platelet basic protein	P02775	108.55%	77.58%		90.12%	91.26%	85.59%	91.89%	75.30%
Platelet factor 4	P02776		72.27%						73.18%
Platelet factor 4 variant	P10720	90.68%							
Properdin	P27918					88.02%	88.33%		57.74%
Protein AMBP	P02760	98.55%	93.71%	87.77%	100.59%	98.01%	83.70%	96.02%	89.74%
Protein Z-dependent protease inhibitor	Q9UK55	105.10%				98.93%		96.32%	92.81%
Prothrombin	P00734	104.78%	82.85%	81.55%	91.11%	101.19%	79.94%	95.28%	79.73%
Retinol-binding protein 4	P02753	102.37%	92.39%	95.22%	99.97%	104.29%	97.26%	99.82%	99.88%
Selenoprotein P	P49908		97.61%	93.89%		102.90%		102.70%	93.87%
Serotransferrin	P02787	100.55%	62.12%	57.53%	85.15%	96.95%	68.44%	82.47%	61.04%
Serum amyloid P-component	P02743	106.43%	86.24%	88.63%	96.51%	99.00%	80.44%	93.45%	73.53%
Serum paraoxonase/arylesterase 1	P27169			71.65%		103.39%	70.00%	89.06%	72.02%
Sex hormone-binding globulin	P04278	102.05%	69.46%	71.40%	85.71%	96.81%	85.93%	84.83%	68.97%
Tetranectin	P05452	86.23%	73.44%	67.78%	88.69%	101.55%	89.05%	87.95%	67.41%
Thrombospondin-1	P07996	108.67%	75.47%						70.49%
Thyroxine-binding globulin	P05543	100.65%	68.63%		40.67%	104.01%	77.23%	90.71%	71.02%
Vitamin D-binding protein	P02774	110.36%	86.51%	91.67%	90.30%	104.14%	85.63%	96.19%	87.00%
Vitronectin	P04004	100.32%	80.93%	79.08%	84.68%	86.22%	84.04%	74.80%	75.04%
von Willebrand factor	P04275	97.43%	74.88%		82.33%	92.34%	83.70%		69.80%
Zinc-alpha-2-glycoprotein	P25311	106.78%		91.76%	88.43%		97.01%		

Supplementary Table 3

Fractional synthesis rate - % new per day (k)

**Plasma Proteins**	**Uniprot Accession**	**Subject #003**	**Subject #004**	**Subject #005**	**Subject #007**	**Subject #008**	**Subject #009**	**Subject #010**	**Subject #011**
Actin, aortic smooth muscle	P62736							≥7.94%	
Afamin	P43652	≥6.98%	6.37%	6.34%	3.76%	≥4.9%	7.23%	≥7.94%	6.13%
Alpha-1-antichymotrypsin	P01011	≥6.98%	7.49%	7.42%	≥4.11%	≥4.9%	7.39%	≥7.94%	6.84%
Alpha-1B-glycoprotein	P04217	≥6.98%	7.51%	7.42%	3.85%	≥4.9%	7.47%	≥7.94%	7.16%
Alpha-2-antiplasmin	P08697	≥6.98%	9.52%	≥11.51%	≥4.11%	≥4.9%	9.13%	≥7.94%	9.10%
Alpha-2-HS-glycoprotein	P02765	≥6.98%	6.23%	6.95%	3.70%	≥4.9%	7.11%	≥7.94%	9.82%
Alpha-2-macroglobulin	P01023	≥6.98%	2.87%	3.03%	2.92%	≥4.9%	4.90%	3.29%	2.52%
Angiotensinogen	P01019	≥6.98%	≥10.47%	≥11.51%	≥4.11%	≥4.9%	8.93%	≥7.94%	≥10.96%
Antithrombin-III	P01008	≥6.98%	8.32%	9.47%	≥4.11%	≥4.9%	9.10%	≥7.94%	9.97%
Apolipoprotein A-II	P02652	≥6.98%	7.03%	9.81%		≥4.9%		≥7.94%	7.81%
Apolipoprotein A-IV	P06727	5.57%	7.11%	6.30%	2.11%	3.43%	8.90%	5.00%	7.70%
Apolipoprotein B-100	P04114	≥6.98%	8.98%	9.71%	≥4.11%	≥4.9%	8.77%	≥7.94%	9.95%
Apolipoprotein E	P02649	≥6.98%	≥10.47%	≥11.51%	1.23%	≥4.9%	≥10.47%	≥7.94%	≥10.96%
Apolipoprotein(a)	P08519		5.24%					5.59%	
Beta-2-glycoprotein 1	P02749	≥6.98%	7.35%	9.22%	3.40%	≥4.9%	6.13%	7.04%	8.84%
Beta-2-microglobulin	P61769						5.81%		
Biotinidase	P43251			6.72%		≥4.9%	7.39%	5.37%	6.27%
Carbonic anhydrase 1	P00915								0.62%
Carboxypeptidase B2	Q96IY4		5.85%			4.68%			
Carboxypeptidase N catalytic chain	P15169		8.53%	8.63%		≥4.9%		≥7.94%	6.22%
Carboxypeptidase N subunit 2	P22792		≥10.47%					≥7.94%	≥10.96%
Ceruloplasmin	P00450	≥6.98%	4.93%	4.95%	3.42%	≥4.9%	5.25%	5.96%	4.62%
Cholinesterase	P06276					≥4.9%			3.25%
Clusterin	P10909	≥6.98%	≥10.47%	≥11.51%	≥4.11%	≥4.9%	≥10.47%	≥7.94%	≥10.96%
Coagulation factor X	P00742		≥10.47%	8.39%		≥4.9%		≥7.94%	10.58%
Coagulation factor XII	P00748	≥6.98%	6.50%	7.58%	3.83%	≥4.9%	7.01%	≥7.94%	6.12%
Complement C1q subcomponent subunit A	P02745	≥6.98%	5.36%	6.06%		≥4.9%	7.72%		
Complement C1q subcomponent subunit B	P02746				2.32%	4.41%	7.83%		
Complement C1q subcomponent subunit C	P02747	≥6.98%	5.13%	4.93%	2.44%	3.67%	8.15%	5.47%	4.31%

Supplementary Table 3

Fractional synthesis rate - % new per day (k)

Plasma Proteins	Uniprot Accession	Subject #003	Subject #004	Subject #005	Subject #007	Subject #008	Subject #009	Subject #010	Subject #011
Complement C1r subcomponent	P00736	≥6.98%	8.44%	10.95%	3.51%	≥4.9%	≥10.47%	6.79%	≥10.96%
Complement C1r subcomponent-like protein	Q9NZP8	6.40%	6.14%	6.76%	2.83%	4.13%	6.75%	5.69%	6.80%
Complement C1s subcomponent	P09871	≥6.98%	8.71%	≥11.51%	≥4.11%	≥4.9%	≥10.47%	7.35%	≥10.96%
Complement C2	P06681	≥6.98%	9.64%	11.12%	≥4.11%	≥4.9%	≥10.47%	≥7.94%	10.44%
Complement C3	P01024	≥6.98%	9.04%				8.42%	≥7.94%	
Complement C4-A	P0C0L4	≥6.98%	9.53%	10.35%	≥4.11%	≥4.9%	8.68%	≥7.94%	9.17%
Complement C4-B	P0C0L5	≥6.98%	9.24%	≥11.51%		≥4.9%	5.71%		
Complement C5	P01031	≥6.98%	7.66%	7.61%	3.90%	≥4.9%	7.35%	≥7.94%	7.88%
Complement component C6	P13671	≥6.98%	≥10.47%	2.24%	3.32%	≥4.9%	10.05%	≥7.94%	6.49%
Complement component C7	P10643	≥6.98%	6.16%	5.72%	3.20%	≥4.9%	9.34%	6.18%	5.33%
Complement component C8 alpha chain	P07357	≥6.98%	8.05%	8.88%	≥4.11%	≥4.9%	8.50%	≥7.94%	7.51%
Complement component C8 beta chain	P07358	≥6.98%	6.32%	8.99%	2.83%	≥4.9%	5.75%	7.78%	7.68%
Complement component C8 gamma chain	P07360	≥6.98%	7.11%	7.62%	4.07%	≥4.9%	8.23%	7.51%	7.82%
Complement component C9	P02748	≥6.98%	8.37%	10.61%	4.08%	≥4.9%	9.30%	≥7.94%	8.51%
Complement factor B	P00751	≥6.98%	8.23%	≥11.51%	≥4.11%	≥4.9%	≥10.47%	≥7.94%	8.92%
Complement factor H	P08603	≥6.98%	5.98%	6.20%	3.70%	≥4.9%	6.58%	7.14%	5.85%
Complement factor I	P05156	≥6.98%	≥10.47%	≥11.51%	≥4.11%	≥4.9%	≥10.47%	≥7.94%	≥10.96%
Corticosteroid-binding globulin	P08185	≥6.98%	5.90%			≥4.9%	6.41%	7.20%	5.75%
Extracellular matrix protein 1	Q16610				2.85%	4.36%	≥10.47%	≥7.94%	
Fibrinogen alpha chain	P02671	≥6.98%	4.81%	7.08%	4.06%	≥4.9%	6.24%	7.53%	6.12%
Fibrinogen beta chain	P02675	6.68%		5.18%	3.63%	≥4.9%	4.92%	5.38%	4.66%
Fibrinogen gamma chain	P02679			5.64%	3.88%	≥4.9%	6.09%	5.66%	4.82%
Fibronectin	P02751	≥6.98%	8.57%	7.95%	3.78%	≥4.9%	7.12%	7.93%	8.62%
Ficolin-3	O75636	≥6.98%	5.88%	11.26%		≥4.9%	5.27%	5.54%	5.74%
Gelsolin	P06396	≥6.98%	7.25%	7.85%	3.34%	≥4.9%	≥10.47%	≥7.94%	7.79%
Glutathione peroxidase 3	P22352			3.77%			5.34%		
Haptoglobin	P00738			8.60%					
Haptoglobin-related protein	P00739		≥10.47%	8.94%					
Hemoglobin subunit alpha	P69905	1.33%					2.26%		0.39%

**Supplementary Table 3**		**Fractional synthesis rate - % new per day (k)**							
**Plasma Proteins**	**Uniprot Accession**	**Subject #003**	**Subject #004**	**Subject #005**	**Subject #007**	**Subject #008**	**Subject #009**	**Subject #010**	**Subject #011**
Hemoglobin subunit beta	P68871	1.19%	0.33%	0.65%			1.93%		0.37%
Hemoglobin subunit delta	P02042						1.21%		
Hemoglobin subunit gamma-1	P69891								0.82%
Hemopexin	P02790	≥6.98%	4.34%	3.43%	2.89%	≥4.9%	2.28%	4.16%	4.01%
Heparin cofactor 2	P05546		5.43%			2.61%		4.95%	3.83%
Histidine-rich glycoprotein	P04196	≥6.98%	≥10.47%	10.25%	≥4.11%	≥4.9%	≥10.47%	≥7.94%	7.44%
Ig gamma-3 chain C region	P01860						3.73%		
Ig kappa chain C region	P01834	1.84%					4.73%		
Ig mu chain C region	P01871						3.39%		
Immunoglobulin lambda-like polypeptide 5	B9A064						3.66%		
Insulin-like growth factor-binding protein complex acid labile subunit	P35858	6.95%	6.28%	8.23%	4.07%		5.88%	6.51%	
Inter-alpha-trypsin inhibitor heavy chain H1	P19827	≥6.98%	8.27%	9.04%	3.95%	≥4.9%	5.82%	≥7.94%	7.97%
Inter-alpha-trypsin inhibitor heavy chain H2	P19823	≥6.98%	7.85%	9.37%	3.56%	≥4.9%	6.61%	≥7.94%	8.50%
Inter-alpha-trypsin inhibitor heavy chain H3	Q06033	≥6.98%	≥10.47%	≥11.51%	≥4.11%	≥4.9%	9.55%	≥7.94%	10.22%
Inter-alpha-trypsin inhibitor heavy chain H4	Q14624	≥6.98%	7.87%	9.05%	3.60%	≥4.9%	7.58%	≥7.94%	7.63%
Kallistatin	P29622	≥6.98%	7.76%	7.69%	3.19%	4.77%	7.38%	3.76%	7.67%
Kininogen-1	P01042	≥6.98%	8.19%	7.53%	≥4.11%	≥4.9%	8.90%	≥7.94%	7.24%
Leucine-rich alpha-2-glycoprotein	P02750	≥6.98%	7.46%	8.00%	≥4.11%	≥4.9%	7.86%	≥7.94%	8.05%
Low affinity immunoglobulin gamma Fc region receptor III-A	P08637						5.17%		
Lumican	P51884	2.60%	1.52%	1.75%	1.22%	1.44%	2.97%	1.81%	1.06%
N-acetylmuramoyl-L-alanine amidase	Q96PD5	≥6.98%	6.87%	8.62%	3.27%	≥4.9%	6.61%	7.43%	8.91%
Peptidase inhibitor 16	Q6UXB8			≥11.51%	2.92%				

Supplementary Table 3

Plasma Proteins	Uniprot Accession	Fractional synthesis rate - % new per day (k) Subject #003	Subject #004	Subject #005	Subject #007	Subject #008	Subject #009	Subject #010	Subject #011
Phosphatidylinositol-glycan-specific phospholipase D	P80108								≥10.96%
Pigment epithelium-derived factor	P36955	≥6.98%	9.81%	≥11.51%	≥4.11%	≥4.9%	≥10.47%	≥7.94%	≥10.96%
Plasma kallikrein	P03952	≥6.98%	5.51%	6.93%	≥4.11%	≥4.9%	5.83%	7.33%	5.62%
Plasma protease C1 inhibitor	P05155	≥6.98%	7.86%	9.81%	3.49%	≥4.9%	≥10.47%	≥7.94%	≥10.96%
Plasminogen	P00747	≥6.98%	7.38%	7.88%	3.26%	≥4.9%	6.99%	6.80%	7.41%
Plasminogen-related protein B	Q02325								3.85%
Platelet basic protein	P02775	≥6.98%	6.80%		≥4.11%	≥4.9%	8.81%	≥7.94%	6.66%
Platelet factor 4	P02776		5.83%						6.27%
Platelet factor 4 variant	P10720	≥6.98%							
Properdin	P27918					4.51%	9.76%		4.10%
Protein AMBP	P02760	≥6.98%	≥10.47%	10.51%	≥4.11%	≥4.9%	8.25%	≥7.94%	10.84%
Protein Z-dependent protease inhibitor	Q9UK55	≥6.98%				≥4.9%		≥7.94%	≥10.96%
Prothrombin	P00734	≥6.98%	8.01%	8.45%	≥4.11%	≥4.9%	7.30%	≥7.94%	7.60%
Retinol-binding protein 4	P02753	≥6.98%	≥10.47%	≥11.51%	≥4.11%	≥4.9%	≥10.47%	≥7.94%	≥10.96%
Selenoprotein P	P49908		≥10.47%	≥11.51%		≥4.9%		≥7.94%	≥10.96%
Serotransferrin	P02787	≥6.98%	4.41%	4.28%	3.41%	≥4.9%	5.24%	6.00%	4.49%
Serum amyloid P-component	P02743	≥6.98%	9.02%	10.87%	≥4.11%	≥4.9%	7.42%	≥7.94%	6.33%
Serum paraoxonase/arylesterase 1	P27169			6.30%		≥4.9%	5.47%	7.63%	6.07%
Sex hormone-binding globulin	P04278	≥6.98%	5.39%	6.26%	3.47%	≥4.9%	8.91%	6.50%	5.57%
Tetranectin	P05452	6.01%	6.03%	5.66%	3.89%	≥4.9%	10.06%	7.30%	5.34%
Thrombospondin-1	P07996	≥6.98%	6.39%						5.81%
Thyroxine-binding globulin	P05543	≥6.98%	5.27%		0.93%	≥4.9%	6.73%	≥7.94%	5.90%
Vitamin D-binding protein	P02774	≥6.98%	9.11%	≥11.51%	≥4.11%	≥4.9%	8.82%	≥7.94%	9.72%
Vitronectin	P04004	≥6.98%	7.53%	7.82%	3.35%	4.22%	8.34%	4.75%	6.61%
von Willebrand factor	P04275	≥6.98%	6.28%		3.10%	≥4.9%	8.25%		5.70%
Zinc-alpha-2-glycoprotein	P25311	≥6.98%		≥11.51%	3.85%		≥10.47%		
